# Supplementary material for: Influence of nitrogen fertilizer rate and variety on tef [Eragrostis tef (Zucc.) Trotter] nutritional composition and sensory quality of a staple bread (Injera)
Source: PLoS One. 2024 Jan 2;19(1):e0295491. doi: 10.1371/journal.pone.0295491 (PMC10760880; doi:10.1371/journal.pone.0295491)
Supplement: Attachment — Submitted filename: PONE-D-22-09743_reviewer_marked.pdf [file pone.0295491.s002.pdf]

PLOS ONE

Influence of Nitrogen Fertilizer Rate and Variety on Tef [Eragrostis tef (Zucc.) Trotter] Nutritional Composition and Sensory Quality of a Staple bread (Injera)

--Manuscript Draft--

**Manuscript Number:**	PONE-D-22-09743
**Article Type:**	Research Article
**Full Title:**	Influence of Nitrogen Fertilizer Rate and Variety on Tef [Eragrostis tef (Zucc.) Trotter] Nutritional Composition and Sensory Quality of a Staple bread (Injera)
**Short Title:**	Influence of Nitrogen Fertilizer Rate and Variety on Tef Nutritional and Sensory Quality of a Injera
**Corresponding Author:**	Hayelom Berhe Dagnaw, Msc<br>Ethiopian Institute of Agricultural Research<br>Addis Ababa, ETHIOPIA
**Keywords:**	Tef, fertilizer rates, Nutrition, Injera, sensory quality, and color CIEL*a*b values
**Abstract:**	In Ethiopia, tef is one of the major staple crops used as a basic raw material for food development such as stable bread called injera. Studies reported that, unbalanced N fertilizer reduces the overall deliciousness of grains. Now a day there is some variation in the quality of injera; this may bring with the increase of chemical fertilizers (N).Therefore, an afield experiment was conducted to assess the effects of N fertilizer rates (0, 30, 60, 90, and 120 kg N ha-1) on grain nutrition and sensory quality of injera of three tef varieties (Kora, Boset, and Asgori) in main cropping season in RCBD with three replications using the same dose of DAP. Recommended parameters were determined using standard methods. Sensory quality and color of injera were determined using panelists and injera eye software respectively. The result showed that; only protein content was increased with N rates, while carbohydrate is decreased significantly at (P<0.05). Varieties with N rate did not show increasing or decreasing trends in mineral content. Using panelists, Kora at the control plot (K0) had better color, flavor, texture, and taste values of injera, while slightly decreased with N rates. Grand means of varieties and N rates did not have a significant difference, while Asgori has a difference of color and taste; injera from white tef had a better acceptance as compared with red color. Injera eye software indicated that, the color of injera was significantly affected by varieties. On average means of varieties, Kora had a higher (55.74) L* value followed by Boset (54.71), with lower L* of Asgori (51.26), had lower injera quality. Injera from the Asgori variety had a maximum red color. Kora and Boset had higher yellow color on the control plot, but for Asgori it increased with the N rate.
**Order of Authors:**	Hayelom Berhe Dagnaw, Msc
	Ashagrie Zewdu Woldegiorgis, PhD
	Kebebew Asefa Abebe, PhD
**Additional Information:**	
**Question**	**Response**
**Financial Disclosure**<br><br>Enter a financial disclosure statement that describes the sources of funding for the work included in this submission. Review the submission guidelines for detailed requirements. View published research articles from *PLOS ONE* for specific examples.<br><br>This statement is required for submission	The first author of this research article is Hayelom Berhe Dagnaw. I am working at Ethiopian Institute of Agricultural Research, Addis Ababa, Ethiopia. I graduated my M.Sc. degree in food Science and Nutrition from Addis Ababa University, Ethiopia.<br>On the other hand, the second author (supervisor) of this research article was Ashagrie Zewdu Woldegiorgis (Ph.D.) with the position of associate professor. He works at Addis Ababa University, center for Food Siena and Nutrition.<br>The third author (supervisor) of this research article was Kebebew Asefa (Ph.D.), plant breeder, with the position of professor (senior researcher). He works at Ethiopian Institute of Agricultural Research, Addis Ababa, Ethiopia. The funders of this research article were Ethiopian Institute of Agricultural Research (EIAR) and Addis Ababa University (AAU). The URL of each funder website (EIAR) and AAU are,

Powered by Editorial Manager® and ProduXion Manager® from Aries Systems Corporation

and **will appear in the published article** if the submission is accepted. Please make sure it is accurate.<br><br>**Unfunded studies**<br>Enter: *The author(s) received no specific funding for this work.*<br><br>**Funded studies**<br>Enter a statement with the following details:<br>• Initials of the authors who received each award<br>• Grant numbers awarded to each author<br>• The full name of each funder<br>• URL of each funder website<br>• Did the sponsors or funders play any role in the study design, data collection and analysis, decision to publish, or preparation of the manuscript?<br>• **NO** - Include this sentence at the end of your statement: *The funders had no role in study design, data collection and analysis, decision to publish, or preparation of the manuscript.*<br>• **YES** - Specify the role(s) played.<br><br>* typeset	www.eiar.gov.et, and www.aau.edu.et, respectively. The funders had no role in study design, data collection and analysis, decision to publish, or preparation of the manuscript.
**Competing Interests**<br><br>Use the instructions below to enter a competing interest statement for this submission. On behalf of all authors, disclose any competing interests that could be perceived to bias this work—acknowledging all financial support and any other relevant financial or non-financial competing interests.<br><br>This statement is required for submission and **will appear in the published article** if the submission is accepted. Please make sure it is accurate and that any funding sources listed in your Funding Information later in the submission form are also declared in your Financial Disclosure statement.<br><br>View published research articles from *PLOS ONE* for specific examples.	The authors have declared that no competing interests exist

Powered by Editorial Manager® and ProduXion Manager® from Aries Systems Corporation

**NO authors have competing interests**<br><br>Enter: *The authors have declared that no competing interests exist.*<br><br>**Authors with competing interests**<br><br>Enter competing interest details beginning with this statement:<br><br>*I have read the journal's policy and the authors of this manuscript have the following competing interests: [insert competing interests here]*<br><br>* typeset	
**Ethics Statement**<br><br>Enter an ethics statement for this submission. This statement is required if the study involved:<br><br>• Human participants<br>• Human specimens or tissue<br>• Vertebrate animals or cephalopods<br>• Vertebrate embryos or tissues<br>• Field research<br><br>Write "N/A" if the submission does not require an ethics statement.<br><br>General guidance is provided below. Consult the submission guidelines for detailed instructions. **Make sure that all information entered here is included in the Methods section of the manuscript.**	there was no institution or relevant body was not as granted per permission

Powered by Editorial Manager® and ProduXion Manager® from Aries Systems Corporation

**Format for specific study types**<br><br>**Human Subject Research (involving human participants and/or tissue)**<br>• Give the name of the institutional review board or ethics committee that approved the study<br>• Include the approval number and/or a statement indicating approval of this research<br>• Indicate the form of consent obtained (written/oral) or the reason that consent was not obtained (e.g. the data were analyzed anonymously)<br><br>**Animal Research (involving vertebrate animals, embryos or tissues)**<br>• Provide the name of the Institutional Animal Care and Use Committee (IACUC) or other relevant ethics board that reviewed the study protocol, and indicate whether they approved this research or granted a formal waiver of ethical approval<br>• Include an approval number if one was obtained<br>• If the study involved *non-human primates*, add *additional details* about animal welfare and steps taken to ameliorate suffering<br>• If anesthesia, euthanasia, or any kind of animal sacrifice is part of the study, include briefly which substances and/or methods were applied<br><br>**Field Research**<br><br>Include the following details if this study involves the collection of plant, animal, or other materials from a natural setting:<br>• Field permit number<br>• Name of the institution or relevant body that granted permission	
**Data Availability**<br><br>Authors are required to make all data underlying the findings described fully available, without restriction, and from the time of publication. PLOS allows rare exceptions to address legal and ethical concerns. See the PLOS Data Policy and FAQ for detailed information.	Yes - all data are fully available without restriction

A Data Availability Statement describing where the data can be found is required at submission. Your answers to this question constitute the Data Availability Statement and **will be published in the article**, if accepted.<br><br>**Important:** Stating 'data available on request from the author' is not sufficient. If your data are only available upon request, select 'No' for the first question and explain your exceptional situation in the text box.<br><br>Do the authors confirm that all data underlying the findings described in their manuscript are fully available without restriction?	
**Describe where the data may be found in full sentences. If you are copying our sample text, replace any instances of XXX with the appropriate details.**<br><br>• If the data are **held or will be held in a public repository**, include URLs, accession numbers or DOIs. If this information will only be available after acceptance, indicate this by ticking the box below. For example: *All XXX files are available from the XXX database (accession number(s) XXX, XXX.).*<br>• If the data are all contained **within the manuscript and/or Supporting Information files**, enter the following: *All relevant data are within the manuscript and its Supporting Information files.*<br>• If neither of these applies but you are able to provide **details of access elsewhere**, with or without limitations, please do so. For example:<br><br>*Data cannot be shared publicly because of [XXX]. Data are available from the XXX Institutional Data Access / Ethics Committee (contact via XXX) for researchers who meet the criteria for access to confidential data.*<br><br>*The data underlying the results presented in the study are available from (include the name of the third party*	All relevant data are within the manuscript and its Supporting Information files

Powered by Editorial Manager® and ProduXion Manager® from Aries Systems Corporation

and contact information or URL).<br>• This text is appropriate if the data are owned by a third party and authors do not have permission to share the data.<br><br>* typeset	
Additional data availability information:	Tick here if the URLs/accession numbers/DOIs will be available only after acceptance of the manuscript for publication so that we can ensure their inclusion before publication.; Tick here if your circumstances are not covered by the questions above and you need the journal’s help to make your data available.

Powered by Editorial Manager® and ProduXion Manager® from Aries Systems Corporation

Cover Letter Click here to access/download;Manuscript;Supportive information.docx

Supportive documents

S1 Table 1: Soil physico-chemical properties of the experimental field based on pre-planting soil sample (0-15 cm depth) analysis

S2 Table 2: Nutritional composition of tef grain as influenced by varieties and N fertilizer rates

S3 Table 3: Means of phytic acid and tannin content of tef grain as influenced by different N fertilizer rates and varieties

S4 Table 4: Mineral contents of tef grain as influenced by the interaction of tef varieties with different N fertilizers rates

S5 Table 5: Sensory acceptability test result of injera of tef varieties as influenced by five different N fertilizer rates

S6 Table 6: Holes/ eyes and color values (CIE-l*ab) of injera of three tef varieties as influenced by N rates using *injera* eye software

Manuscript

Click here to access/download;Manuscript;Manuscript (Hayelom Berhe Dagnaw).docx

Influence of Nitrogen Fertilizer Rate and Variety on Tef *[Eragrostis tef (Zucc.) Trotter]* Nutritional Composition and Sensory Quality of a Staple bread *(Injera)*

Hayelom Berhe Dagnaw [1] Ashagrie Zewdu Woldegiorgis[2] Kebebew Assefa[3]

1-Ethiopian Institute of Agricultural Research, Department of Food Science and Nutrition, Addis Ababa, Ethiopia

2 -Center for Food Science and Nutrition, Faculty of Natural and Computational Science, Addis Ababa University, Addis Ababa, Ethiopia

3- Ethiopian Institute of Agricultural Research, Plant breeder, Addis Ababa, Ethiopia

Correspondence should be addressed to Hayelom Berhe Dagnaw; hayelom.berhe8r@gmail.com

Abstract

In Ethiopia, tef is one of the major staple crops used as a basic raw material for food development such as stable bread called injera. Studies reported that, unbalanced N fertilizer reduces the overall deliciousness of grains. Now a day there is some variation in the quality of injera; this may bring with the ~~increase of chemical fertilizers (N)~~.Therefore, an afield experiment was conducted to assess the effects of N fertilizer rates (0, 30, 60, 90, and 120 kg N ha^{-1}) on grain nutrition and sensory quality of injera of three tef varieties (*Kora*, *Boset*, *and Asgori*) in main cropping season in RCBD with three replications using the same dose of DAP. Recommended parameters were determined using standard methods. Sensory quality and color of injera were determined using panelists and injera eye software respectively. The result showed that; only protein content was increased with N rates, while carbohydrate is decreased significantly at (P<0.05). Varieties with N rate did not show increasing or decreasing trends in mineral content. Using panelists, Kora at the control plot (K0) had better color, flavor, texture, and taste values of injera, while slightly decreased with N rates. Grand means of varieties and N rates did not have a significant difference, while Asgori has a difference of color and taste; injera from white tef had a better acceptance as compared with red color. Injera eye software indicated that, the color of injera was significantly affected by varieties. On average means of varieties, Kora had a higher (55.74) L* value followed by Boset (54.71), with lower L* of Asgori (51.26), had lower injera quality. Injera from the Asgori variety had a maximum red color. Kora and Boset had higher yellow color on the control plot, but for Asgori it increased with the N rate.

Keywords: Tef, fertilizer rates, Nutrition, Injera, sensory quality, and color CIEL*a*b values

Introduction

Tef *(Eragrostis tef (Zucc) Trotter)* is grass species that originated from Ethiopia **[1, 2, 3, 4]**. Tef is cultivated the same as wheat and barley cultivated in Ethiopia, and maturity period and geographical locations have a significant variation on tef cultivars. Tef is grownup during the main growing season and is mainly cultivated as a mono-crop, but infrequently under multiple cropping systems and very resistant to various environmental conditions such as drought and waterlogging **[5, 6]**.

Tef is an indigenous and highly nutritious cereal crop used as a raw material for product development and it is a staple food that plays a vital role in the country's food and nutrition security that accounts for about 15% of calories **[7, 8, 9].**

In Ethiopia, the productivity of tef is below the potential and this is due to the reason of yield-limiting factor of poor soil fertility and other factors, currently the average national productivity of tef is1.664 t/ha **[10]**, which is very low as compared to other cereal crops **[11]**. *Mwangi* **[11]**, reported that the use of inorganic fertilizer is critical to increasing yield. Application of N fertilizer is considered essential to improve yield productions of cereal crops such as tef [**12**]. But a study using N fertilizer rates with twelve rice cultivars **[13]**, revealed that grain yield reduced as N rates increased because of excessive vegetative growth and increased lodging and pest damage.

The most important constraint in tef production is its inherent low productivity. This has, among others, been due to the inability of farmers to use the required quantities of mineral nutrients and the use of unbalanced chemical fertilizer applications **[14].** The application of optimum fertilizer rates for specific soil types greatly contributes to yield enhancement. Organized studies should be conducted under varying conditions and in various regions to determine the fertilizer requirements of tef for optimizing yield. Moreover, to understand optimum yields of the crop, appropriate fertilizer rates have to be used since this may vary according to the soil type and weather conditions of the study area. One of the alternative ways for the additions of yield production is the use of appropriate fertilizers which should be a source of all indispensable nutrients. Therefore, plant nutrition depends on the availability and uptake of macro and micronutrients contained in the soil **[15]**.

Nitrogen is a major mineral element used in agricultural practices. But studies show that an application of different chemical fertilizer rates on different crop types may have an effect on their yield production **[16].** *Schuphan* ***[17, 18]***, reported that excess N application diminishes taste and flavor and reduces the biological values of plant protein, such as potatoes. GU **[13]**, stated that, as levels of the N fertilizer system are increased, it significantly decreased the overall palatability of rice notwithstanding of the cultivars. Protein contents increased with N fertilizers and the palatability of food may also be significantly affected. This may be high N rates results in the decline of grain quality and the tastiness was significantly and negatively correlated with high protein content. In dissimilarity protein content at high N rates, the lower concentrations of amylose improve the cooking and eating qualities **[13]**.

However, many studies have been performed on tef which deal about the interaction of organic or inorganic chemical fertilizers with soil and their effects on grain yield and grain nutritional profiles of cereal crops, but theses studied was not focused on sensory qualities of cereal-based food products such as injera. However, there is some variation in the quality of injera from the last decades. The reason behind this is still controversial. This may probably due to the overuse of chemical fertilizers for increasing yield production. Therefore, this study mainly focused on the Influence of Nitrogen Fertilizer Rate and Variety on Tef Nutritional Composition and Sensory Quality of a Staple bread (Injera).

Material and Methods

Study area

The study was carried out during the 2017 cropping seasons at Debre Zeit Agricultural Research Center (DZARC) from August-November during the main cropping season of 2017. DZARC is located at 47 km to the southeast of Addis Ababa. The experimental site is characterized by heavy black soil which is the dominant soil of the study area with high water retention capacity by its nature. The place is located at 8°44' N latitude and 38°58' E longitude at an altitude of 1860 meters above sea level and receives an annual average rainfall of about 832.

Soil sample characterization

Pre-plant soil samples (0-15 cm) were taken and analyzed for total nitrogen (N) by Kjeldahl method **[19]**, available phosphorous (P) by **[20]**, organic matter content by **[21]** and soil pH was determined using a potentiometer method. Electrical conductivity was measured using a conductivity meter.

Experimental design

The experimental design was a randomized complete block design (RCBD) in a factorial combination of N fertilizer with three replications. The fertilizer treatments were five N levels (0, 30, 60, 90, and 120 kg ha^{-1}) with the same dose of Triple Super Phosphate (TSP) (10 kg ha^{-1}), as a source of P which is the recommended one for Vertisol of DZARC. The source of N is urea. A full dose of P and half of N was applied at planting while the remaining N dose was applied 30 days later when the plant is in the active vegetative growth stage. The experimental plot size was 5 m x 3 m with a total area of 47 m x 37 m. The distance between plots and blocks was 1 and 1.5 m, respectively. An improved three tef variety of Kora (DZ-Cr-438RIL133B, white color), Boset (DZ-Cr-409 RIL50d, white color), and Asgori (Dz-01-99, red color) were used. All fertilizers were applied as row-side bands. The seeds amounting to 37.5 g per plot based on the recommended seeding rate of 15 kg ha-1 were sown by hand drilling in the rows.

Grain flour preparation

For the preparation of flour, first, the grain was cleaned and sieved using 1 mm sieve size and the flour was prepared using a milling machine having the model number of EN5501, cyclone sample mill, USA-AID with 0.5 mm of sieve size.

Injera Preparations

Using tef flour and water as ingredients, injera was prepared according to Boka and Yetnberk **[22, 23].** Therefore, 200 g tef flour was mixed with 180 ml water and starter culture, which was prepared from each sample for individual dough making, and then the mixture with starter culture, was kneaded and mixed well for 5 minutes. The dough was fermented for 24 to 48 hours for 28 to 30 ^{o}c. After the dough was fermented, 100 ml of water was added and the batter was fermented for 2 - 3 hrs. @ 28-30 ^{0}c and waited till foam and bubbles were formed. After the fermentation has occurred and the foams and bubbles were formed, about 500 ml of the batter was poured in a curricular manner; on 50 cm diameter hot clay griddle *mitad* and back cover for about 2 minutes. Then Injera was removed by lifting it off the hot griddle and sliding over a straw mat and the final leavened, flat round Ethiopian bread-like product, Injera was obtained.

Image analysis

The color of the injera for scored values for the required parameters was determined after 24 hours the injera were baked. To brighten the sample two parallel fluorescent lamps were used. To obtained a uniform light intensity; the samples were located at 10 cm overhead at the angle 45o of the sample plane. The room was switched off in order to protect light reflection from the

interior walls before receiving the images of injera. A camera with a model of the Samsung Galaxy J5 prime camera 13 pixels with a resolution of 720 x 1280 pixels was used. The camera was located vertically at a distance of 45 cm from the injera sample and images were captured on the camera setting and no flash. For each formula, a total of two injera were captured. Finally, the images were transferred to a computer hard disk and opened with injera eye software. Colors (CIE L *a*b) of injera were measure on the digital image of the sample visualized on the monitor using injera eye software **[24]** pointing the cursor on the surface area of the sample and clicking on it. Two surface points from each injera sample were taken by cutting using a cutter having closely related maximum size and standard deviations of injera samples. Therefore, the number of holes (holes of injera and filtered eyes) and Color of injera (lightness (L*, scale 0 to 100), a* (redness), and b* (blue-yellow) color) were determined using injera eye software.

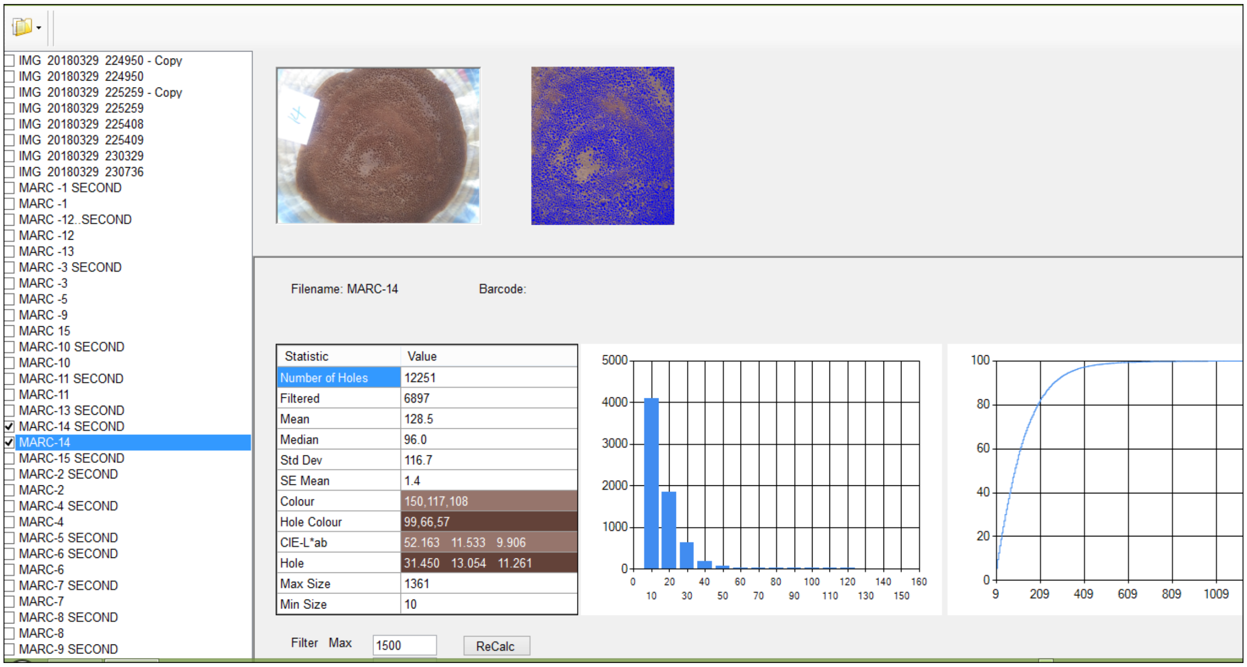

Figure 1: Image analysis of injera using injera software **[24].**

Laboratory Analysis

The proximate composition was determined according to the official method **AOAC, [25].** Carbohydrate content was determined by subtracting the sum percentage of moisture, ash, crude protein, crude fat, and crude fiber from 100%.

The energy value was obtained based on the values of crude protein, crude fat, and total carbohydrate which were multiplied with factors of 4, 9, and 4 respectively and reported in kilocalories per 100g, [(Energy (kcal/100 g) = (4* %Protein) + (4*%CHO) + (9*%Fat)].

Mineral contents of Iron (Fe), calcium (Ca), Zinc (Zn), and Phosphorus (P) were determined using the AAS method and phosphomolybdate blue method for phosphorus content, respectively, AACC **[26].** The ant-nutritional factor of phytic acid and Tannin content was determined using the methods of **[27, 28].** The total and filtered number of eyes and color of injera was determined using injera eyes software **[24].**

Statistical Analysis

All the parameters were measured in triplicates and the results are reported as mean ± standard error (S.E). And, the relationship between treatments and injera sensory qualities was analyzed by SPSS 20.0 window evaluation version program using Duncan's multiple range post-hoc test using two-way ANOVA, and significance was established at $\alpha \leq 0.05$.

Result and discussion

Soil physicochemical properties of the experimental site

The physiochemical properties of composite soil sample before planting are presented in (Table 1). Soil electrical conductivity (EC) and pH values were analyzed using a 1:1 soil-water ratio using the paste saturation method. The result affects nutrient availability and toxicity. *Tilahun,* **[29]** reported from the study of *Forth and Ellis, (1997),* which is in line with the present findings, the average pH value of (6.5) (Table 1) indicated that the experimental soil type is classified as slightly acidic soil. From the result, available P showed in low a range which is below the threshold levels of in tropical soils **[30, 31]**. As cited by *Mirutse*, **[32]** the total percentage of N is 0.124% and the response of tef to N indicated that the available form of N could be inadequate and it is likely to be stored in organic matter and clay minerals. The total mean percentage of organic carbon (1.41%) content of the experimental site was comparable to soils in the semiarid regions. Due to the lower number of organic materials applied to the soil and complete removal of the biomass from the field, most cultivated soils of Ethiopia are poor in organic matter **[33]**.

Table 1: Soil physico-chemical properties of the experimental field based on pre-planting soil sample (0-15 cm depth) analysis

S.S (0-15cm) depth	EC (ds/cm) 1:1	P^H (H_2O) 1:1	%OC	%OM	%TN	Ava. P (ppm)
Block -1	0.181	6.83	1.51	2.61	0.130	7.212
Block -2	0.226	6.37	1.39	2.40	0.120	7.205
Block-3	0.215	6.31	1.41	2.43	0.122	6.928
Mean ± SEM	0.207±0.170	6.50 ± 0.093	1.44 ± 0.037	2.48 ± 0.066	0.124 ± 0.003	7.12 ± 0.094

EC = electrical conductivity, pH= power of hydrogen ion, OC-= organic carbon, Om = organic matter, TN = total nitrogen, Ava. P = Available phosphorus, S. S=Soil sample and SEM=standard errors of means.

Nutritional Compositions of Tef Grain as Influenced by Nitrogen Fertilizer Rates and Varieties

Moisture content

The result indicated that, interactions of N rates and varieties were not significantly affected the moisture contents (Table 2). The moisture content of tef grain recorded from the various fertilizer treatments was in the ranges reported by Bultosa G. (2007) as, 9-11%. Hence, the moisture contents for brown seeded Asgori variety (DZ-01-99) and the white seeded tef varieties (DZ-01-196) ranged from 12.14 to 13.00% and 11.99-12.23%, respectively **[34].** And the moisture content was not significantly affected by both the average means of varieties and N rates. On the other hand, for N rate (over all means of tef varieties), the moisture content at 60kg ha^{-1} have significant variation as compared with 90 and 120 kg ha^{-1} N rate (Table 2).

Ash contents of tef grain

Ash in tef grain is an indication of inorganic elements that are present in food as minerals. Both varieties and N rates did not show significant effects on ash content and it was ranged between 1.99 to 3.16% and this is in line with values reported ~~by~~ **[35].** On the other hand, **[35]** obtained the maximum ash content of 3.16% for the Asgori variety, whereas the present result showed an ash content of 2.42% for this same variety. This suggested that the applications of N fertilizer could not alter the contents of inorganic elements. And, for N rate (over all means of tef varieties) was not significantly affected the ash contents (Table 2

Protein contents of grain

The result showed that protein content was highly significantly ($P<0.01$) affected by the grand means of N fertilizer rates, but not by means of varieties (Table 1). Means of N rates indicated that protein content was ranged from 9.51% -11.14%, but not much deviated from reported by

[35], 8.7% to 11%. The increase in protein content with the N rate is not unexpected since N forms a major constituent of proteins. When we see the N rates (over all means of tef varieties), the protein content were increase linearly with N rates (Table). This indicated that N rates have significantly effects on the crude protein contents of tef varieties.

Table 2: **Nutritional composition of tef grain as influenced by varieties and N fertilizer rates**

**Treatments**	**Parameters**						
**VWR**	**%Moisture**	**%Ash**	**%Protein**	**%Crude fat**	**% fiber**	**%Carbo hydrate**	**Energy (Kcal)**
K0	9.88 ± [illegible]033^{a}	2.48 ± 0.025 ^{a}	9.97 ± 0.587^{a}	2.08 ± 0.050^{a}	2.86 ± 0.025^{a}	75.59 ± 0.659^{a}	361.00 ± 0.343^{a}
K30	9.83 ±0.088 ^{a}	2.50 ± 0.191^{a}	9.50 ± 0.469^{a}	2.16 ± 0.028^{a}	3.46 ± 0.032^{a}	76.00 ±0.512^{a}	361.47 ± 0.681^{a}
K60	10.13 ± 0.058^{a}	2.38 ± 0.134^{a}	10.61 ± 0.242^{a}	2.13 ± 0.062^{a}	2.60 ± 0.045^{a}	74.75 ± 0.424 ^{a}	360.60 ± 0.337^{a}
K90	9.97 ± 0.033^{a}	2.41 ± 0.017^{a}	10.39 ± 0819^{a}	2.24 ± 0.026^{a}	3.07 ± 0.104^{a}	74.99 ± 0.791^{a}	361.65 ± 0.471^{a}
K120	9.73 ±0.122 ^{a}	2.53 ± 0.184^{a}	11.7 ± 0.094^{a}	2.20 ± 0.029^{a}	3.03 ± 0.110^{a}	73.77 ± 0.416^{a}	361.98 ± 0.0503 ^{a}
**G.MK**	**9.909 ± 0.107**	**2.46 ± 0.112**	**10.434 ± 0.654**	**2.162 ± 0.048**	**3.004 ± 0.145**	**75.02 ± 0.689**	**361.34 ± 0.537**
B0	9.87 ± 0.067 ^{a}	2.58 ± 0.210 ^{a}	9.57 ± 0.029 ^{a}	2.55 ± 0.362^{a}	3.40 ± 0.054^{a}	75.42 ± 0.605^{a}	362.94 ± 0.746^{a}
B30	10.28 ± 0.033^{a}	2.29 ± 0.038^{a}	10.35 ± 0.319^{a}	2.15 ± 0.050^{a}	2.50 ± 0.025^{a}	74.93 ± 0.371^{a}	360.46 ± 0.274^{a}
B60	9.91 ± 0.200^{a}	2.32 ± 0.092^{a}	10.57 ± 0.600^{a}	2.09 ± 0.013^{a}	2.48 ± 0.012^{a}	75.10 ± 0.698^{a}	361.56 ± 0.801^{a}
B90	9.70 ± 0.058^{a}	2.40 ± 0.159^{a}	11.51 ± 0.492^{a}	2.14 ± 0.009 ^{a}	2.74 ± 0.011^{a}	74.25 ± 0.701^{a}	362.27 ± 0.830^{a}
B120	9.81 ± 0.029 ^{a}	2.23 ± 0.092^{a}	11.21 ± 0.100^{a}	2.10 ±0.048^{a}	2.70 ± 0.102^{a}	74.59 ± 0.245^{a}	362.66 ± 0.279^{a}
**G.MB**	**9.91 ± 0.177**	**2.37 ± 0.129**	**10.40 ± 0.506**	**2.16 ± 0.167**	**2.76 ± 0.103**	**74.83 ± 0.518**	**362.08 ± 0.740**
A0	9.99 ±0.088 ^{a}	2.30 ± 0.015 ^{a}	8.99 ± 0.159^{a}	2.24 ± 0.015^{a}	3.73 ± 0.114^{a}	76.47 ± 0.416^{a}	362.00 ± 0.920^{a}
A30	10.00 ± 0.088^{a}	2.39 ± 0.029^{a}	10.58 ± 0.537^{a}	2.21 ± 0.053^{a}	2.92 ± 0.101^{a}	74.82 ± 0.654^{a}	361.46 ± 0.429^{a}
A60	10.07 ± 0.219^{a}	2.45 ± 0.152^{a}	10.79 ± 0.182^{a}	2.17 ± 0.015^{a}	3.35 ± 0.113^{a}	74.51 ± 0.157^{a}	360.71 ± 0.466^{a}
A90	9.60 ± 0.088^{a}	2.41 ± 0.113^{a}	10.98 ± 0.065^{a}	2.16 ± 0.026^{a}	2.84 ± 0.054^{a}	74.85 ± 0.193^{a}	362.74 ± 0.649^{a}
1230	9.86 ± 0.120^{a}	2.52 ± 0.120^{a}	10.42 ± 0.229^{a}	2.18 ± 0.037^{a}	2.72 ± 0.049^{a}	75.02 ± 0.519^{a}	361.39 ± 0.545^{a}
**G.MA**	**9.904 ± 0.151**	**2.42 ± 0.096**	**10.67 ±0.485**	**2.19 ± 0.033**	**3.11 ± 0.115**	**75.14 ± 0.568**	**361.66 ± 0.849**
**Means of varieties (over all N rates)**							
Kora	9.91 ±0.056^{a}	2.46 ± 0.053^{a}	10.45 ± 0.183^{a}	2.16 ± 0.045^{a}	3.00 ± 0.149^{a}	75.02 ± 0.227^{a}	361.34 ± 0.328^{a}
Boset	9.92 ±0.056^{a}	2.36 ± 0.053^{a}	10.64 ± 0.183^{a}	2.22 ± 0.045^{a}	2.76 ± 0.149^{a}	74.86 ± 0.227^{a}	361.98 ± 0.328^{a}
Asgori	9.91 ± 0.056^{a}	2.42 ± 0.053^{a}	10.35 ± 0.183^{a}	2.19 ± 0.045^{a}	3.11 ± 0.149^{a}	75.13 ± 0.227^{a}	361.66 ± 0.328^{a}
**G.MV**	**9.91 ± 0.076**	**2.41 ± 0.108**	**10.48 ± 0.529**	**2.19 ± 0.107**	**2.96 ± 0.307**	**75.00 ± 0.219**	**361.66 ± 0.807**
**N rates (Over all means of tef varieties)**							
0	9.98 ± 0.138ab	2.46 ± 0.127^{a}	9.51 ± 0391^{c}	2.29 ± 0.219^{a}	2.41 ± 0.012^{a}	75.83 ± 0.568^{a}	361.98 ± 0.783^{a}
30	10.02 ± 0.115ab	2.40 ± 0.112^{a}	10.14 ± 0448^{bc}	2.17 ± 0.041^{a}	2.22 ± 0.001^{a}	75.25 ± 0.560^{ab}	361.13 ± 0.514^{a}
60	10.19 ± 0.186a	2.38 ± 0.118^{a}	10.66 ± 0.341^{ab}	2.13 ± 0.38^{a}	2.37 ± 0.011^{a}	74.79 ± 0.442^{b}	360.96 ± 0.892^{a}
90	9.79 ± 0.084b	2.41 ± 0.098^{a}	10.96 ± 0.555^{a}	2.18 ± 0.03^{4a}	2.53 ± 0.018^{a}	74.70 ± 0.572^{b}	362.22 ± 0.638^{a}
120	9.89 ± 0.110b	2.43 ± 0.129^{a}	11.14 ± 0.398^{a}	2.18 ± 0.036^{a}	2.38 ± 0032^{a}	74.46 ± 0.423^{b}	362.01 ± 0.607^{a}

**G.MR**	**9.97 ± 0.107**	**2.41 ± 0.113**	**10.48 ± 0.544**	**2.19 ± 0.104a**	**2.61 ± 0.51a**	**75.00 ± 0.568**	**361.66 0.731**

Values are the means of triplicate experiments. Results are reported as mean ± S.EM, VWR = Varieties of tef with N Rates (kg N ha^{-1}), GMK, GMB, G.MA and G.MR = grand means of Kora, Boset and Asgori tef varieties, and N rates, respectively. Kora (K0, K30, K60, K90, K120), Boset (B0, B30, B60, B90, B120), and Asgori (A0, A30, A60, A90, 120) combining with 0, 30, 60, 90 and 120 N rates (kg N ha^{-1}) for the treatment number of 1 to 15., and S. EM = Standards of errors of means. Means in the same column and within the same treatment category followed by different letter are significantly different as judged by LSD at $p < 0.05$**. Note: G.M of Kora, Boset and Asgori varieties are considered as means of varieties (over all N rates).**

Crude fat content

The analysis of variance indicated that; crude fat content the grain was not affected by tef varieties and N fertilizer rates (Table 2); and the ranged was found between 2.16 to 2.22 % and this was in line with **[35] (**2.0 to 3.0 %. The fat accumulations of tef varieties were uniform and have not shown significant differences and of a constant ratio the endosperm and embryo growth by giving optimal conditions to physiological process as described for corn as affected by organic fertilizers **[36].** And, from the present result the N rates (over all means of tef varieties) and means of varieties (over all N rates) have not significant effects on the oil contents of tef varieties.

Crude fiber

The crude fiber content of the grains was not significantly affected by varieties and N levels (Table 2), as well as the N rates (over all means of tef varieties). And the range of crude fiber content was found between 2.76 to 3.11%, which is in line with the report ~~of~~ **[37].** Both N rates (over all means of tef varieties) and means of varieties (over all N rates) (Table 2) did not significant affect the fiber contents tef varieties.

Total C[illegible]bohydrate and energy content

Means of N rates had highly significant ($P<0.05$) effects on carbohydrate (CHO) content, but not by varieties and N rates (Table 2). On the other hand, N rates (over all means of tef varieties), 0 kg ha-1 (the control plot) has a maximum CHO as compared with the remains, and it is decreased as level of N rates increases. Carbohydrate content was decreased with increasing N levels and this was inversely related to the protein contents. But neither the different N rates nor the N rates (over all means of tef varieties) and means of varieties exerted a significant effects on the energy and carbohydrate content of tef grains.

Effects of Nitrogen Fertilizer Rates and Varieties on the Anti-nutritional Factor of Tef

Phytic acid content

The interaction N rates with varieties were highly significant at ($P<0.001$) on phytic acid content of tef grain. Phytic acid from control plots was comparable with those receiving different rates of N fertilizer (Table 3). This is because phytate is a primary P storage in the plant, and; a higher phytic acid is expected with fertilization. According to **[37]**, the phytate content for tef varieties ranged from 682 to 1374 mg 100 g-1 on a dry weight basis, and this not in line with present findings (98.2 to114.3 mg 100 g^{-1}) and this may due to the reason whether a methodology or P may be forming a complex with soil and this can be reducing the phytate contents. On the other

hand, means of varieties (over all N rates) and N rates (over all means of tef varieties) did not significantly affect the phytic acid contents of tef grains (Table 3).

Table 3: Means of phytic acid and tannin content of tef grain as influenced by different N fertilizer rates and varieties

**Treatments**	**Parameters (mg 100 g $^{-1}$)**	
**VWR**	**Phytic acid**	**Tannin**
G0	103.7 ± $^{1.49b}$	31.46 ± 1.021^{a}
K30	114.3 ± 6.659^{ab}	29.25 ± 0.865^{a}
K60	101.1 ± 0.495 ^{b}	28.49 ± 0.803^{a}
K90	98.2 ± 0.192^{b}	27.88 ± 0.154^{a}
K120	108.8 ± 3.668^{ab}	28.12 ± 0.828^{a}
**G.MK**	**105.22 ± 2.255**	**29.04** ± 1.08
B0	104.3 ± 2.045^{b}	16.86 ±0.109 ^{a}
B30	98.4 ± 0.785^{b}	20.17 ± 0.901^{a}
B60	123.3 ± 10.991^{a}	24.22 ± 0.683^{a}
B90	108.0 ± 3.078^{ab}	21.63 ± 0.913^{a}
B120	109.1 ± 4.009^{ab}	18.05 ± 0.798^{a}
**G.MB**	**108.62 ± 4.458**	**20.17 ± 1.002**
A0	114.0 ± 7.961^{ab}	22.10 ± 0.867^{a}
A30	100.7 ± 0.959^{b}	13.87 ± 0.147^{a}
A60	114.6 ± 7.008^{ab}	16.29 ± 0.128^{a}
A90	103.1 ± 4.049 ^{b}	15.83 ± 0.106^{a}
A120	108.6 ± 4.146^{ab}	16.34 ± 0.108^{a}
**G.MA**	108.20 ± 4.245	16.87 ± 0.149
**Means of varieties (over all N rates)**		
Kora	105.22 ± 1.05^{a}	29.04 ± 1.044^{a}
Boset	108.61 ± 1.505^{a}	20.19 ± 1.044^{b}
Asgori	108.19 ± 1.505^{a}	16.89 ± 1.044^{c}
**G.MV**	**107.34 ± 1.501**	**22. 04 ± 0.904**
**N rates (Over all means of tef varieties)**		
0	109.10 ± 0.588^{a}	15.50 ± 0.865^{a}
30	99.75 ± 0.459^{a}	13.73 ± 0.943^{a}
60	112.73 ± 6.412^{a}	14.73 ± 1.007^{a}
90	108.88 ± 5.001^{a}	12.90 ± 0.982^{a}
120	106.27 ± 4.102^{a}	14.41 ± 0.932^{a}
**G.MR**	**107.35 ± 3.941**	**14.25 ± 0.920**

Values are the means of triplicate experiments. Results are reported as mean ± SEM, VWR = varieties of tef with N rates (kg N ha^{-1}), **GMK, GMB, KMA, , G.MV, G.MR** = grand means of **Kora (K), Boset (B) and Asgori (A)** tef varieties, and N rates respectively. And, (K0, K30, K60, K90, K120), (B0, B30, B60, B90, B120), and (A0, A30, A60, A90, 120) combining with 0, 30, 60, 90 and 120 N rates (kg N ha^{-1}) for the treatment number of 1 to 15, and S. EM = Standards of errors of means. Means in the same column and within the same treatment category followed by different letter are significantly different as judged by LSD at $p < 0.05$**. Note: G.M of Kora, Boset and Asgori tef varieties are considered as means of tef varieties (over all N rates).**

Tannin contents of tef grain

The tannin content of tef flour was highly significantly (P<0.001) affected only by varieties (Table 3). From the means of varieties, both white seeds of Kora and Boset had higher (29.04) and (20.19) mg 100 g^{-1} tannin contents respectively. On the other hand, brown seeded Asgori variety had 16.89 mg CE 100 g-1 tannin contents and this is almost similar to a report of **[37]** and *Gebreab* (unpublished M.sc thesis); **[38]**; and this may be due probably to genetic differences of varieties. Tannin content is significantly by N rates (Over all means of tef varieties) (Table 3).

Effects of Nitrogen Fertilizer Rates on Mineral Contents of Tef Grain

Iron (Fe)

The mineral content of Iron (Fe) was significantly (P<0.05) affected by varieties and the interaction with N fertilizer rates (Table 4). The range of Fe contents for both white seeded (Kora and Boset) tef varieties were found between 25.85 to 34.58 mg 100 g-1, respectively; but for Asgori was found between 23.33 to 37.39 mg 100 g-1. The present result is in line with **[37]** that the ranges of Fe content of white tef varieties were found between 9.5-37.7, while for red tef variety was exists between 11.6 - > 150 mg 100 g-1; but not in line with the report of *Abebe* **[39]**, showing red tef variety had a higher Fe content than white tef varieties. These high contents of Iron of tef varieties may also bring from soil contamination **[39]**. In addition to this, as compared with other cereal crops, tef has higher iron contents; this may due to the surface areas of tef itself. The mineral content of Iron is significantly affected by means of varieties (over all N rates), but not by N rates (over all means of tef varieties) (Table 4).

Table 4: Mineral contents of tef grain as influenced by the interaction of tef varieties with different N fertilizers rates

**Treatments**	**Parameters**		
**VWR**	**P(ppm)**	**Fe (mg 100 g^{-1})**	**Ca (mg 100 g^{-1})**
K0	216.7 ± 0.087^{cde}	27.63 ± 0.826^{defg}	57.67 ± 0.729^{de}
K30	216.9 ± 0.197^{bcde}	27.41 ± 0.496^{defg}	72.83 ± 0.883^{bc}
K60	217.5 ± 0.144^{ab}	25.85 ± 0.796^{efg}	68.14 ± 0.942^{abcd}
K90	216.9 ± 0.108^{bcde}	29.12 ± 0.906^{cde}	73.89 ± 0.971^{ab}
K120	216.6 ± 0.232^{de}	28.02 ± 0.883^{def}	68.60 ± 0.189^{abcd}
**G.MA**	**216.91 ± 0.229**	**27.606 ± 0.881**	**68.226 ± 0.192**
B0	216.6 ± 0.275^{e}	28.49 ± 0.463^{cd}	74.85 ± 0.978^{a}
B30	217.4 ± 0.101^{ab}	28.11 ± 0.623^{def}	61.67 ± 0.924^{de}
B60	217.8 ± 0.475^{a}	32.57 ± 0.704^{bc}	63.38 ± 0.298^{bcde}
B90	216.4 ± 0.117^{e}	34.58 ± 0.719^{ab}	65.13 ± 0.689^{abcde}
B120	216.7 ± 0.060^{cde}	26.96 ± 0.691^{defg}	64.63 ± $0.0.783^{abcde}$
**G.MA**	**216.92 ± 0.402**	**30.14 ± 0.807**	**65.93 ± 0.829**

A0	217.2 ± 0.271^{abc}	23.33 ± 0.161^{g}	62.85 ± 0.089^{cde}
A30	217.1± 0.202^{bcd}	30.25 ± 0.708^{cd}	62.46 ± 0.109 ^{cde}
A60	217.2 ± 0.434^{abcd}	24.22 ± 0.184^{fg}	57.09 ± 0.029^{e}
A90	216.7 ± 0.266^{cde}	36.49 ± 0.807^{ab}	58.84 ± 0.106^{de}
A120	217.3 ± 0197^{abc}	37.39 ± 0.781^{a}	58.49 ± 0.149^{de}
**G.MA**	**217.10 ± 0.270**	**30.334 ± 0.709**	**59.95 ± 0.245**
**Means of varieties (over all N rates)**			
Kora	216.91 ± 0.101^{a}	22.60 ±0.638 ^{b}	68.22 ± 1.70^{a}
Boset	216.92 ± 0.101^{a}	30.14 ± 0.638^{a}	65.93 ± 1.70^{a}
Asgori	217.10 ± 0.101^{a}	30.38 ± 0.638^{a}	59.95 ± 1.70^{b}
**G.MV**	**216.98 ± 0.507**	**27.71 ± 0.381**	**64.70 ± 1.87**
**N rates (Over all means of tef varieties)**			
0	216.78 ± 0.293^{bc}	28.08 ± 0.128^{a}	58.07 ± 0.801^{a}
30	217.14 ± 0.200^{ab}	28.19 ± 0.208^{a}	60.84 ± 0.708^{a}
60	216.78 ± 0.362^{ab}	31.01 ± 0.807^{a}	59.07 ± 0.184^{a}
90	217.14 ± 0.204^{c}	30.83 ± 0.609^{a}	60.05 ± 0.419^{a}
120	216.78 ± 0.244^{bc}	31.08 ± 0.809^{a}	61.73 ± 0.147^{a}
**G.MR**	**217.14 ± 0.307**	**29.838 ± 0.709**	**59.95 ± 0.909**

Values are the means of triplicate experiments. Results are reported as mean ± SEM, VWR = varieties of tef with N rates (kg N ha^{-1}), **G.MK, G.MB, G.MA, G.MV, G.MR** = grand means of **Kora (K), Boset (B) and Asgori (A)** tef varieties, and N rates respectively. And, **(K0, K30, K60, K90, K120), (B0, B30, B60, B90, B120), and (A0, A30, A60, A90, 120)** combining with 0, 30, 60, 90 and 120 N rates (kg N ha^{-1}) for the treatment number of 1 to 15. And S. EM = Standards of errors of means, P =Phosphorus, Fe= Iron and Ca= Calcium and; means in the same column and within the same treatment category followed by different letter are significantly different as judged by LSD at $p < 0.05$**. Note: G.M of Kora, Boset and Asgori tef varieties are considered as means of tef varieties (over all N rates).**

Calcium (Ca)

Calcium (Ca) content was significantly ($P<0.05$) affected by varieties and the interaction effects (Table 4). Both Kora and Boset white tef varieties have a better calcium contents as compared with Red Asgori tef varieties (Table 4). The maximum (73.89, 74.85, and 62.85) mg 100 g^{-1} Ca content were observed at plots with 90 (Kora), 0 (Boset and Asgori) kg N ha-1, while the minimum (57.67, 61.67, and 57.09) mg 100 g-1 were recorded at 0, 30 and 60 kg N ha-1 of Kora, Boset and Asgori, respectively. The ranges of Ca content for both white tef varieties of Kora and Boset were found between 57. 67 to 74.85; while for Asgori was found between 57.09 to 62.85 mg 100 g-1, respectively and this result also in agreements with **[37].** Regardless of N fertilizer rates, the maximum Ca content was observed from Kora (68.22) tef variety followed by Boset (65.93), but the minimum (59.95) mg 100 g-1 was recorded from Asgori. This was not in line again with the findings of Abebe et al **[39]**, that red tef has higher Ca content than white tef varieties. From the current result, N rates (over all means of tef varieties) did not significantly affect the mineral content of Calcium (Table 4).

Phosphorus content of tef grain

The analysis of variance indicated that the P content of tef grain was significantly ($P<0.05$) affected by N rates, but not by varieties. As reported by *Kebede* **[40]** showing that, red tef grain has higher P content, but this is not in line with the present result, that P contents of the two white-seeded and one brown seeded Asgori varieties had almost the same P mineral content (Table 4). Regardless of tef varieties, the maximum P content was recorded at 60 kg N ha^{-1} followed by 30 kg N ha-1. This implies that P and N fertilizer interactions have a significant effect on the concentrations of P of tef flour. In addition to this, N rates (over all means of tef varieties) have slightly significant effects on phosphorus content.

Effects of Nitrogen Fertilizer Rates and Varieties on Sensory Quality of Injera

The analysis of sensory qualities and acceptability of tef injera made from three tef varieties as influenced by different N fertilizer rates are presented in (Table 5). The sensory evaluation scored values for all sensory attributes were determined after 24 hours the injera was baking using 15 semi-trained panelists.

Sensory evaluations of Injera

Color

The analysis of variance indicated that, the color of injera was significantly ($P < 0.05$) affected by tef varieties and N fertilizer rates. The color of injera was slightly decreased with the increase of the first three N fertilizer rates. However, Kora had a maximum (8.27) color value of injera at the control plot (K0) and rated as like-very much. The grand means of a variety of Kora (G.MK) (6.63), Boset (G. MB) (6.53), and Asgori (G.MA) (6.04) showed that the color of baked injera was slightly affected by tef varieties. Average means of TN fertilizer rates indicated that, the color of injera becomes maximum at the control plot (without N fertilizer) and decreased with the first four N rates (0 to 90 kg N ha^{-1}), but slightly increased at 120 kg N ha^{-1} and this may be due probably to N fixation and this negatively affects the availability of N for plant use.

The response of panelists' values indicated the sensory evaluations of injera were not significantly different. Injera appearance tells is the eye on the surface of the injera is a honeycomb-like structure of the top surface formed when CO_2 gas bubbled **[41].** Injera with uniformly distributed eyes on its top surface with no blind spot on its surface is indicative of good injera and color of injera affected by consumer preferences **[42].**

Flavor

Only the interaction effect of varieties with N fertilizer rates significantly ($P<0.05$) affected the flavor of injera but not have increasing or decreasing trends, and this is not in line with **[13]**

which reported that increasing N fertilizer application affects the flavor of a food product. Kora (7.1) and Boset (6.07) have a better color of injera as comparing with Asgori (5.73) and rated as neither like nor a dislike, like-slightly, and like moderately, respectively. This may be due to the reason that injera from red tef is already acumen by the societies and they may not care during their evaluations. Average overall N rates and tef varieties indicated no significant differences in the flavor of injera (Table 5). Grand means of Kora (G.MK) 6.13, Boset (G. MB) 6.05 and Asgori (G.MA) 6.04 had rated values of like – slightly, and means of N rate showed that application of all N rates except 90 kg N ha-1(neither like nor dislike) had the same effects on the flavor of injera and rated as like-slightly.

Texture

Texture indicated that the quality of food products and is determined by touch and refers to the degree of fluffiness, roughness, smoothness, hardness, or softness. Injera which is soft and easily pliable to wrap and hold the sauce *(wot)* becomes most preferable by consumers **[42]**. From the response of panelists and the analysis of variance, the texture of injera was not significant ($P<0.05$) affected by tef varieties and N rates (Table 5). However, the interaction of both white seeded varieties of (Kora and Boset) and brown seeded Asgori variety had the same effects on the texture of injera and rated as like slightly, and neither like nor dislike, respectively. *Gebrehiwot* **[43]**, reported that injera from only tef flour has no variation in texture values and this was not in line with our present study.

Taste

From the response of the panelists, the interaction of varieties and N rates had the same effects on injera taste value and had greater than 6 (like-slightly), except injera from K90 and A90, both had 5.87 taste values and rated as neither like nor dislike. The sensory evaluations of taste were ranged from 5.87 to 7.27 (K0), neither like nor dislike to like-moderately (Table 5), and this is not in line with findings of **[41],** who reported that taste value of injera from tef flour was rated as excellent. And injera from only tef varieties was not significant for taste values as compared with injera o E. curvula grain **[43],** and this was not in line with our present study and this implies that applications of N fertilizer have significant effects on taste values of baked injera.

Mean values of varieties, taste for injera was significant ($P<0.05$); that both white seeded varieties of Kora (6.55) and Boset (6.61) have the same taste values; but not Asgori variety.

This difference may be due to the reason that less attitude in which the societies gave for injera prepared from red tef (Table 5).

Folding capacity (FC)

The folding capacity of injera was not significantly affected by varieties and N rates (Table 5). However, the maximum (7.00) value was recorded from K0 (like-moderately) and the minimum (5.80) was observed at K90 and B60 (neither like nor dislike), while all the rest of baked injera from the three varieties with the application of N fertilizer rates was rated as like-slightly.

Eye size (ES) and eye distribution (ED) of injera

Injera is traditional, a chief food in Ethiopia, and is large pancake-like bread prepared from cereal crops such as tef. Injera is characterized by having 'eyes' (honeycomb-like holes) on its upper surface, which are produced due to the generating's and escaping of carbon dioxide **[44]** during the baking and fermentation process; which contributed to eye productions and distribution on the surface of baked injera.

In the analysis of variance from the response of panelists, the eye size (ES) of baked injera was significantly ($P<0.05$) affected by N fertilizer rates but not by varieties (Table 5). From this baked injera with different N fertilizer rates, except K60, B60, B90, and A120 (neither like nor dislike) and K0, K30, and A30 (like-moderately), the remaining baked injera was rated as like-slightly. This indicated that, the maximum grain yield (plants use the optimum N fertilizer) also has a significant connection with the changes in eye size. This may due probably that optimal N fertilizer has negative important effects on carbon dioxide formations, which have an essential role in the eye size of the injera.

On the other hand, eye distributions (ED) of injera were significantly ($P<0.05$) affected by varieties and N rates. The ED of injera rated values of neither like nor dislike responses were recorded at B0 (5.87), B90 (5.67), A60 (5.87), and A90 (5.73), and like moderately was obtained at K30 (7.33). The average mean of varieties showed that, ED of injera had significant differences between Kora (6.63), and Boset (6.00) varieties, while Asgori (6.15) had almost the same ED as Kora. The performance of ES and ED mainly depends on the formation and outflows of carbon dioxide gas **[44]**; thus, from this point of view, it is not clear to hypothesize and develop a correlation that applications of N fertilizer and productions of carbon dioxide gas significantly affects the ES and ED of injera.

Top and bottom surfaces (TBS) of injera

The appearances of the top and bottom surface of baked injera were not significantly ($p < 0.05$) affected by varieties and N rates (Table 5). From the response of panelists, all baked injera was rated as like-slightly except injera from B0 and B120 (like-moderately). The study by yetnberk et al. (2005) indicated that, injera made from tef flour had a white top and bottom surface; which is in line with the present result and N rates did not improve or reduced the whiteness of the top and bottom surface of injera baked from the three tef varieties.

Table 5: Sensory acceptability test result of injera of tef varieties as influenced by five different N fertilizer rates

Treatment	Sensory attributes								
VWR	Color	Flavor	Texture	Taste	FC	ES	ED	TBS	OACC
K0	8.27±0.182^{a}	7.13±0.336^{a}	6.87±0.401^{a}	7.27±0.358^{a}	7.00±0.390^{a}	7.27±0.419^{a}	6.73±0.511^{ab}	6.87±0.506^{a}	7.80±0.355^{a}
K30	7.07±0.300^{abc}	5.93±0.442^{ab}	5.93±0.408^{ab}	6.13±0.559^{ab}	6.07± 0.581^{a}	7.27±0.419^{a}	7.33±0.287^{a}	6.60±0.486^{a}	7.47±0.350^{ab}
K60	5.73±0.530^{c}	6.00±0.447^{ab}	6.13±0.363^{ab}	6.80±0.223^{ab}	6.27± 0.565^{a}	5.87±0.487^{abc}	6.27±0.371^{ab}	6.67±0.333^{a}	6.87±0.335^{abc}
K90	5.87±0.559^{bc}	5.53±0.487^{b}	5.93±0.442^{ab}	5.87±0.363^{c}	5.93 ±0.371^{a}	6.33±0.532^{abc}	6.47±0.363^{ab}	6.87±0.363^{a}	6.87±0.456^{abc}
K120	6.20±0.460^{bc}	6.07±0.483^{ab}	6.07± 0.521^{ab}	6.67±0.398^{ab}	6.33 ± 0.410^{a}	6.67±0.252^{abc}	6.33±0.303^{ab}	6.87±0.256^{a}	7.20±0.312^{abc}
**G.MK**	**6.63 ± 0.217^{a}**	**6.13 ± 0.202^{a}**	**6.19 ± 0.191^{a}**	**6.55 ± 0.191^{a}**	**6.32 ± 0.210^{a}**	**6.68 ± 0.198^{a}**	**6.63 ± 0.170^{a}**	**6.77 ±0.175^{a}**	**7.24± 0.168^{a}**
B0	6.60±0.375^{bc}	6.07±0.383^{ab}	6.20±0.428^{ab}	6.80±0.500^{ab}	6.80 ±0.327^{a}	6.60±0.335^{abc}	5.87±0.435^{b}	7.07±0.330^{a}	6.87±0.236^{abc}
B30	6.33±0.454^{bc}	6.20 ± 0.428^{ab}	6.80± 0.312^{ab}	6.20 ±0.518^{ab}	6.20 ±0.449^{a}	6.47±0.456^{abc}	6.33±0.398^{ab}	6.47±0.446^{a}	6.93±0.316^{abc}
B60	6.47± 0.487^{bc}	6.47±0.401^{ab}	5.87± 0.456^{ab}	6.47±0.336^{ab}	5.80 ±0.393^{a}	5.60 ±0.349^{c}	6.00±0.458^{ab}	6.73±0.371^{a}	6.60±0.349^{abc}
B90	5.93± 0.521^{bc}	5.40±0.533^{c}	6.07±0.419^{ab}	6.73±0.300^{ab}	6.27 ±0.396 ^{a}	5.87±0.477^{abc}	5.67±0.433^{b}	6.40±0.445^{a}	6.80±0.327^{abc}
B120	7.33± 0.485^{ab}	6.13± 0.524^{ab}	6.47± 0.413^{ab}	6.87±0.376^{ab}	6.60 ±0.363^{a}	6.60±0.400^{abc}	6.40±0.423^{ab}	7.40±0.349^{a}	7.07±0.521^{abc}
**G. MB**	**6.53 ± 0.210^{a}**	**6.05 ± 0.203^{a}**	**6.28 ± 0.188^{a}**	**6.61 ± 0.82^{a}**	**6.33 ±0.173^{a}**	**6.23 ± 0.184^{a}**	**6.00 ± 0.190^{b}**	**6.81 ± 0.176^{a}**	**6.85±0.158^{ab}**
A0	6.00±0.447^{bc}	5.73±0.345^{ab}	5.40±0.400^{b}	6.00±0.276^{ab}	6.20 ±0.355^{a}	6.20 ±0.380^{abc}	6.13±0.413^{ab}	6.13±0.401^{a}	6.40±0.445^{bc}
A30	5.53± 0.551^{c}	6.27±0.419^{ab}	5.87±0.446^{ab}	6.27±0.442^{ab}	6.80 ±0.355^{a}	7.13±0.350^{ab}	6.73±0.300^{ab}	6.93±0.345^{a}	7.20±0.296^{abc}
A60	6.20±0.460^{bc}	6.00±0.390^{ab}	5.87± 0.307$^{a\ b}$	6.00±0.324^{ab}	6.07 ± 0.452^{a}	6.27±0.384^{abc}	5.87±0.435^{b}	6.00±0.516^{a}	6.60±0.375^{abc}
A90	6.13±0.524^{bc}	6.27±0.358^{ab}	5.80±0.460^{ab}	5.87±0.350^{c}	6.60 ± 0.434^{a}	6.40±0.412^{abc}	5.73±0.530^{b}	6.07±0.539^{a}	6.13±0.542^{c}
A120	6.33±0.607^{bc}	5.93±0.581^{ab}	5.80±0.449^{ab}	6.07±0.473^{ab}	6.40 ± 0.476^{a}	5.73±0.605^{abc}	6.27±0.483^{ab}	6.27±0.463^{a}	6.60±0.375^{abc}
**G.MA**	**6.04 ± 0.229^{b}**	**6.04 ± 0.165^{a}**	**5.75 ± 0.182^{a}**	**6.04 ± 0.182^{b}**	**6.41 ± 0.184^{a}**	**6.35 ± 0.197^{a}**	**6.15 ±0.195^{ab}**	**6.28 ± 0.203^{a}**	**6.59 ±0.1854^{b}**
**N Rates (Overall tef varieties)**									
0	6.96 ±0.2461^{a}	6.31±0.220^{a}	6.16 ±0.248^{a}	6.69 ± 0.33^{a}	6.67 ±0.208^{a}	6.89±0.224^{ab}	6.24±0.262^{ab}	6.69±0.244^{a}	7.02±0.256^{a}
30	6.31±0.268^{ab}	6.13±0.243^{a}	6.20±0.230^{a}	6.20±0.287^{a}	6.36±0.270^{a}	6.95±0.328^{a}	6.80±0.198^{a}	6.67±0.244^{a}	7.20±0.171^{a}
60	6.13±0.270^{ab}	6.16±0.236^{a}	5.96 ±0.215^{a}	6.42±0.176^{a}	6.04±0.270^{a}	5.91±0.235^{c}	6.04±0.240^{b}	6.47±0.239^{a}	6.69±0.217^{a}
90	5.98 ± 0.302^{b}	5.73± 0.268^{a}	5.93±0.249^{a}	6.16±0.201^{a}	6.27±0.237^{a}	6.20±0.271b^{c}	5.87±0.257^{b}	6.44±0.218^{a}	6.60±0.258^{a}
120	6.62 ± 0.304^{ab}	6.04± 0.260^{a}	5.80 ± 0.259^{a}	6.53 ± 0.241^{a}	6.44 ± 0.237^{a}	6.33±0.258^{ab}	6.33±0.231^{ab}	6.84±0.218^{a}	6.96±0.222^{a}
**G.M**	6.40±0.127	6.08±0.110	5.75±0.105	6.40± 0.103	6.36±0.109	6.42±0.112	6.26±0.108	6.62 ± 0.108	6.89 ± 0.099

Values are the means of triplicate experiments. Results were reported as **mean ± SEM**, **VWR** = varieties of tef with N rates (kg N ha^{-1}), **GMK, GMB, KMA** = grand means of Kora, Boset and Asgori tef varieties respectively, **FC** = folding capacity, **ES** = eye size, **ED** = eye distribution, **TBS** = top and bottom surface, **OACC** = over all acceptability of injera, **SEM** = standard error of mean and 0**, 30, 60, 90 and 120** are N rates for the treatment number of 1 to 15. Means in the same column and within the same treatment category followed by different letter are significantly different as judged by LSD at $p < 0.05$**. Note: G.M of Kora, Boset and Asgori varieties are considered as means of varieties (over all N rates**

Overall acceptability of injera quality

Overall acceptability is the results of the combinations of sensory evaluations by panelists of a product. The overall acceptability of injera was significantly (P<0.05) affected by varieties but not N rates. The maximum and minimum values of acceptability of injera were recorded at K0 (7.80) and A90 (6.13), respectively. From the response of panelists, almost all baked injera except K0, K30, K120, B120, and A30 (like-moderately) were rated as like-slightly (Table 5).

Means of varieties showed that, injera from Kora had a maximum (7.24) value of OAIQ and rated as like-moderately, and had the same OAIQ with Boset (6.85). And injera from Kora (7.24) and Asgori (6.59) had significant differences and this minor difference in acceptability may become from the societies perception and comprehension of their appreciations to ward red tef injera (Asgori) as compared with white tef injera (Kora and Boset) preference. *Gebrehiwo*t **[43]** reported that, overall acceptability of injera prepared from tef was accepted 100% and this is not agreed with the present.

Considering all sensory attributes into account, there was a statistical difference among the injera prepared from the three varieties as influenced with N rates and all treatments scored a mean rating above 6 (like-slightly) (Table 5) and this is indicative of the goodness of injera.

Total number of holes and filtered eyes of injera

The color or physical appearance and number of holes of baked injera from three tef varieties as influenced with different N fertilizer rates are presented in (Table 6).

Number of holes of injera

A number of holes of injera are the total number of holes or eyes that were present on the surface of injera and highly significantly (P<0.01) affected by N rates and varieties but not increased or decreased trends (Table 6). Obtaining a higher number of holes on the surface of injera may due to the higher amount of carbon dioxide or gas bubbles in the fermented batter. Thus, the number of holes is not in line with responses of the panelists (Table 5) on the eye size and distributions that the maximum eye size and distribution were obtained from K0 and K30 respectively which are not in agreement with a number of holes results using injera eye software. Therefore, as compared to both results from panelists and the injera eye software, there was a significant difference among the parameters of the eye size and eye distribution (from panelists) and the number of holes from the injera eye software technology. Being subjective sensory evaluation can vary from observer to observer, using instruments is better. Grand means of Kora (G.MK),

Boset (G. MB), and Asgori (G.MA) of the three tef varieties respectively indicated that the number of holes of injera was significantly affected. The maximum (19609.20) number of holes was recorded from Boset (G. MB) followed by Kora (G.MK) (17187.00) and Asgori (G.MA) (17764.50) varieties, respectively. Regardless of varieties, the average means N rate implies that, applications of 60 kg N ha-1 produced the maximum (21409.83) number of holes.

Filtered eyes of injera

The filtered eyes of injera were highly significant ($P < 0.01$) affected by N rates (Table 6). For Kora tef variety, all N rates have almost the same filtered eyes on the surface of injera, except the minimum was obtained from K0 (7227.50) and the maximum at K30 (12476.50). This implies that the lower number of holes from the control plot (without N fertilizer) had the lower filtered eyes on the surface of injera i.e., the higher the number of holes of injera, the higher the filtered eyes. Boset variety had the maximum (15927.50) filtered eyes of injera at B60, while the minimum (6348.50) was recorded from B90 followed by B0 (8028.00). The ranges of filtered eyes of injera for Asgori tef variety were found between 7166.50 (A30) to 13807.00 (A60) respectively.

Grand means of varieties were significant on the filtered eyes of injera. Thus, Asgori had better-filtered eyes (11295.80) followed by both Kora (10715.60) and Boset (10369.00), respectively. This may show that carbon dioxide is produced during the fermentation process and plays a fundamental role in the formation of cellular structure leavened bread of Asgori **[44]**. Means of N rates indicated that, maximum (13217.83) filtered eyes on the surface of injera were obtained at 60 kg N ha-1, while the minimum (8741.17) at 0 kg N ha-1. Based on this, N fertilizer rate had positive significant effects on the productions of eyes on the surface of injera and the optimum N rate for this could be 60 g N ha-1, but it is not clear that the relation of N rates affected the formations of carbon dioxide or gas bubbles, it may the factor for eye formations and it needs further investigations.

Colors (CIE L *a*b*) values of injera

Lightness (L) color of injera

CIE-l*a*b measures the lightness, redness, and yellowness color of injera in the respective order of L, a, and b. The lightness (L-value) determines the better quality of injera quality. The more the lightness (L* value), the lower the redness (a), and yellowness (b) value that has the better quality of injera. The present result indicated that, the lightness color of injera was highly

significant (P<0.01) affected by N rates and varieties (Table 6). All L*values reported in this result were in the same range reported by **[45].**

Interactions of Kora and Boset varieties with N rates had the same L* values of injera, but injera from Boset was slightly increased with N rates and B90 (56.62) was maximum, but Asgori had a minimum L* value from the control plot 47.59 (A0). The control plot and the maximum N rate (120 kg N ha-1) had the same effects on the L*value, indicated that as the N fertilizer becomes optimum, and it may not be available for plant use. Average means of tef varieties indicated that, Kora had a maximum (55.74) L*value followed by Boset (54.71), and the least value was recorded from Asgori variety; while means of N rate implies that, applications of 30 kg N ha^{-1} could produce better L*value (Table 6).

Redness (a*) color of injera

The analysis of variance showed that the red color of injera was highly significant (P<0.01) affected by varieties and significantly (P<0.05) affected by N rates and the interaction with varieties (Table 6). For both white seeded Kora and Boset varieties, N rates have the same effects on the redness of injera, therefore, this magnifies that the redness color of injera much depends on the grain color, the genetic values.

From grand means of Kora (G.MK), Boset (G. MB) and Asgori (G.MA) tef varieties the maximum (11.01) redness of injera was observed at Asgori followed by Kora (1.146) and the least (0.949) was a recorded form Boset variety. Therefore, the higher the values of redness color, the lower the acceptance of injera. In accordance with this, the red color of injera from Asgori maybe existed due to genetic differences that the grain color of Asgori is red. This may the reason that injera prepared from red tef grain mostly gets a lower chance to accept by the consumers. While, the lower redness color of injera from white seeded varieties indicted that, the injera had a better acceptance as compared with the Asgori variety.

Table 6: Holes/ eyes and color values (CIE-l*ab) of injera of three tef varieties as influenced by N rates using *injera* eye software

**Treatment**	**Parameters**				
	**Holes or eyes of injera**		**Color of injera**		
**VWR**	**Number of holes**	**Filtered eyes**	**Lightness L***	**Redness a***	**Yellowness b***
K0	14934.00±264.000^{ef}	7227.50±234.500^{fg}	55.20±0.173^{bc}	1.90±0.036^{c}	5.14±0.621^{e}
K30	18350.00±1119.000^{bcd}	12476.50±430.500^{c}	54.64±0.404^{bcd}	0.84±0.091^{d}	3.64±0.270^{f}
K60	16031.50±586.500^{de}	9919.00±215.000^{e}	56.82±0.197^{ab}	1.03±0.086^{d}	3.67±0.872^{f}
K90	18300.00±305.000^{bcd}	12131.50±84.500^{c}	57.02±0.013^{ab}	1.21±0.091^{d}	3.72±0.268^{f}
K120	18321.50±982.500^{bcd}	11823.50±416.500^{cd}	55.03±1.213bc	0.76±0.188^{d}	4.51±0.049^{ef}
**G.MK**	17187.40±537.769^{b}	10715.60± 659.992^{b}	55.74 ± 0.488^{a}	1.146±0.140^{b}	4.94±0.729^{b}
B0	20028.50±533.500^{b}	8028.00±423.000^{f}	53.42±0.140^{de}	1.11±0.288^{d}	6.23±0.200^{d}
B30	18930.50±1126.500^{bc}	10885.00±57.000^{de}	54.33±0.725^{cde}	1.06±0.289^{d}	4.93±0.189^{e}
B60	27679.00±1026.000^{a}	15927.50±199.500^{a}	55.21±0.195^{bc}	0.75±0.001^{d}	3.90±0.003^{ef}
B90	11172.00±1622.000^{g}	6348.50±555.500^{g}	56.62±0.151^{ab}	0.73±0.186^{d}	0.85±0.067^{g}
B120	20236.00±166.000^{b}	10656.00±66.000^{de}	53.97±0.032^{cde}	1.09±0.108^{d}	6.16±0.342^{d}
**G.MB**	19609.20 ± 1780.150^{a}	10369.00±1089.599^{b}	54.71 ±0.784^{b}	0.949 ±0.089^{c}	4.41±0.662^{c}
A0	20545.50±266.500^{b}	10968.00±785.000^{de}	47.59±2.073^{h}	11.15±0.147^{a}	7.37±0.648^{c}
A30	12907.00±495.000^{fg}	7166.50±203.500^{fg}	53.41±0.183^{cdf}	10.98±0.085^{ab}	9.95±0.266^{ab}
A60	20519.00±57.000^{b}	13807.00±252.000^{b}	52.14±0.184^{efg}	10.63±0.085^{b}	9.84±0.266^{ab}
A90	16934.00±87.000^{cde}	12767.00±76.000^{c}	52.51±0.000^{defg}	11.22±0.000^{a}	10.99±0.000^{a}
A120	17917.00±802.000^{bcd}	11770.50±373.500^{cd}	50.66±0.197^{g}	11.05±0.012^{ab}	10.36±0.010^{a}
**G.MA**	17764.50± 979.849^{b}	11295.80 ± 770.582^{a}	51.26 ±0.748^{c}	11.01 ±0.074^{a}	9.704±0.426^{a}
**N Rates (Overall means of tef varieties)**					
0	18502.67±1144.903^{b}	8741.17±757.572^{d}	51.40±1.489^{d}	4.72±2.041^{a}	7.58±0.587^{a}
0	16729.17±1287.067^{bc}	10176.00±1002.732^{c}	54.13±0.320^{bc}	4.29±2.116^{b}	6.17±1.223^{b}
60	21409.83±2166.755^{a}	13217.83±1117.176^{a}	54.72±0.874^{b}	4.14±2.056^{b}	5.81±1.299^{ab}
90	15468.67±1445.832^{c}	10415.67±1299.646^{c}	55.45±1.250^{ab}	4.39±2.163^{b}	5.19±1.909^{ab}
120	18824.83±560.057^{b}	11416.67±281.266^{b}	53.22±0.891^{c}	4.30±2.137^{b}	7.01±1.105^{a}
**G.MR**	**18187.034 ±508.81**	**10793.468 ± 295.087**	**53.784 ± 0608**	**4.368 ± 1.088**	**6.352 ± 1.285**

Values are means of replicate experiments. Results were reported as **mean ± SEM**, **VWR** = varieties (tef) with N rates (kg N ha^{-1}), **G. MK, G. MB, K.MA** = grand means of Kora, Boset and Asgori respectively, **SEM** = standard error of mean, K = Kora, **B**= Boset and **A =** Asgori tef varieties and **0, 30, 60, 90 and 120** are N rates for the treatment number of **1 to 5 for each variety**. Means in the same column and within the same treatment category followed by different letters are significantly different as judged by LSD at P<0.05. **NOTE: G.M of Kora, Boset and Asgori are considered also as grand means of varieties (overall N rates).**

Yellowness (b*) color of injera

The yellowness color of injera was highly significantly (P<0.01) affected by varieties and N rates including the interaction effects (Table 6). The ranges of yellowness of injera were found between 0.85 (B90) to 11.99 (A90), respectively. Both Kora and Boset varieties have maximum yellowness color of injera at control plots of K0 (5.14) and B0 (6.23), respectively. This may imply as the applications of N rate becomes maximum, its availability could be reduced and this may also due to the fixation, leaching process, and the control plot and maximum N rate had the

same yellowness color of injera. While Asgori tef variety had the maximum (11.99 and 10.36) yellowness of injera recorded at A90 and A120 respectively. This yellowness color of injera indicated that injera prepared from Asgori tef variety had the least acceptance as compared with Kora and Boset varieties. This idea fits with the consumer's preference they give superiority for injera of white color from white tef varieties.

The rating means of tef varieties indicated that Asgori tef variety had the maximum (9.74) yellowness color of injera followed by Boset (4.41) and Kora (4.41) tef varieties (Table 6). On the other hand, means of N rates indicates that 0 (control plot) had slightly maximum redness color and 0 and 120 kg ha-1 had better yellowness color of injera (Table 6).

Conclusions

The study pointed out that N rate had almost negligible effects on grain nutritional compositions, grand means of N fertilizer indicated that, protein content was increased while carbohydrate was decreased. Tannin content was only significantly affected by tef varieties and this may due probably to genetic differences among tef varieties. The subjective analysis of injera indicated that, sensory qualities did not show any significant effects. From tef varieties, Kora at the control plot (without N fertilizers) had a better color, flavor, texture, and taste values of injera, but slightly decreased with N rates. Regardless of the N rates, the mean varieties of color, flavor, texture, and tats values had no significant difference, except injera from Asgori which had a significant difference in color and taste values. This may be the reason that injera prepared from red tef variety have less acceptance by the society. Thus, the society had lower awareness, that injera prepared from white tef had better acceptance as compared with red tef. Grand means of N rates had no significant differences on flavor, texture, and taste including color quality of injera. Analysis result using injera eye software showed that, N rates and varieties interaction effects did not show significant changes in the color of injera. In general, a difference in the color of injera was happened due to various differences. Based on this it could not be concluded that N rates alone had significant effects on sensory quality and color of injera, rather further study could be needed based on the recommended blended mineral fertilizers associated with soil type and microbial interactions and fermentation kinetics of the dough.

Supportive documents

S1 Table 1: Soil physico-chemical properties of the experimental field based on pre-planting soil sample (0-15 cm depth) analysis

S2 Table 2: Nutritional composition of tef grain as influenced by varieties and N fertilizer rates

S3 Table 3: Means of phytic acid and tannin content of tef grain as influenced by different N fertilizer rates and varieties

S4 Table 4: Mineral contents of tef grain as influenced by the interaction of tef varieties with different N fertilizers rates

S5 Table 5: Sensory acceptability test result of injera of tef varieties as influenced by five different N fertilizer rates

S6 Table 6: Holes/ eyes and color values (CIE-l*ab) of injera of three tef varieties as influenced by N rates using *injera* eye software

Acknowledgment

We would like to give great gratitude and appreciation to the Ethiopian Institute of Agricultural Research (EIAR) and Addis Ababa University (AAU) for supported this research financially. We would like also to acknowledge Debre Zeit Agricultural Research Center, tef research staff, EIAR research laboratory, AAU laboratory, and Melkassa Agricultural Research Center, food science and nutrition research staff for their valuable assistance during this research accomplishment. Our gratitude extends to Mr. Glen Fox Cereal Grain Science Food security, Queensland Alliance for Agricultural and Food Innovation (AQQFI), the University of Queensland, for his consent to use injera eye software via Melkassa agricultural research center, food science, and nutrition research team.

Author Contributions

Conceptualization: Hayelom Berhe Dagnaw.

Data curation: Hayelom Berhe Dagnaw.

Formal analysis: Hayelom Berhe Dagnaw.

Investigation: Hayelom Berhe Dagnaw.

Methodology: Hayelom Berhe Dagnaw, Ashagrie Zewdu, and Kebebew Assefa Abebe

Supervision: Ashagrie Zewdu, and Kebebew Assefa Abebe.

Validation: Ashagrie Zewdu, and Kebebew Assefa Abebe.

Writing – original draft: Hayelom Berhe Dagnaw.

Writing – review & editing: Ashagrie Zewdu, and Kebebew Assefa Abebe.

References

[1]. Bekele, E. & Lester, R. Biochemical assessment of the relationships of Eragrostis tef (Zucc.) Trotter with some wild Eragrostis species (Gramineae). *Annals of Botany, 1981; 48(5), 717-725.*

[2]. Akhtar, T.The cytology of Eragrostis with special reference to E. tef and its relatives. The University of London, 1986.

[3]. Costanza, S. H., Dewet, J.M. & Harlan, J.R. Literature review and numerical taxonomy of Eragrostis tef (T'ef). *Economic Botany,1979; 33: 413-424.*

[4]. Kebede, H.,Johnson, R. & Ferries, D. Photosynthetic response of Eragrostis tef to temperature. *Physiology Plant, 1989; 77, 262-266.*

[5]. Ketema, S. Tef. Eragrostis tef (Zucc.) Trotter. Promoting the conservation and use of underutilized and neglected crops. Institute of plant genetics and crop plant research, Gatersleben/ International plant genetic resources institute, Rome, Italy, 1997; 7-10.

[6]. Assefa, K., Aliye, S., Belay, G., Metaferia, G., Tefera, H., & Sorrells, E,. Quncho: the first popular tef variety in Ethiopia. *International Journal of Agricultural Sustainability, 2011; 9(1), 25-34.*

[7]. Bultosa, G., Hall, A. & Taylor, J. Physico- chemical characterization of Grain Tef [Eragrostis tef (Zucc.) Trotter] starch. *Starch‐ Stärke, 2002; 54(10): 461-468.*

[8]. Gamboa, P.A. Teff "Survey on the nutritional and health aspects of teff (Eragrostis Tef)". *Instituto Tecnológico de Costa Rica, Sede Central.2008;*

[9]. Fufa, B., Behute, B.,& Berhe, T. Strengthening the tef value chain in Ethiopia. Ethiopian Agricultural Transformation Agency, Addis Ababa, Ethiopia. 2011;

[10]. Central statistical agency (CSA). Agricultural sample survey, 2016/17 (2009 E.C.), Vol. I. *Report on area and production of major crops, (Private Peasant Holdings, Meher Season), Statistical Bulletin 584, Addis Ababa, Ethiopia.* 2017; 2016/17 (2009 E.C.),

[11]. Mwangi, W.M., (1997). Low use of fertilizers and low productivity in sub-Saharan Africa. *Nutrient Cycling in Agroecosystems. 1997; 47: 135-147,*

[12]. Mamo, T.,Erksso,T., MEsfin, A. & Selamyihun, K. Review of soil fertility studies conducted on tef: Experience of the Alemaya University of Agriculture. *Alemaya University of Agriculture, Ethiopia, Mimeo. 1996;*

[13]. Gu, J., Chen, J., Chen, L., Wang, Z., Zhang, H. & Yang, J. Grain quality changes and responses to nitrogen fertilizer of japonica rice cultivars released in the Yangtze river basin from the 1950s to 2000s. *The Crop Journal, 2015; 3(4): 285-297.*

[14]. Nyalemegbe, K.., Hotsonyame, G.., Ofori, F. & Osakpa,T. Comparative study of the efficacy of actyva compound fertilizer (N23 P10 K5 3S 2MgO 0.3Zn) on maize cultivation in the coastal savanna and the humid forest ecologies of Ghana. *International Research Journal of Agricultural Science and Soil Science, 2012; 2(1), 008-016.*

[15]. Torres-olives,V., Rodriguez-Martinezt,.A., Valdez-Aguilar, L.A & Alia Te jacal, I. Role of nitrogen and nutrients in crop nutrition. *Journal of agricultural science and Technology, 2014; 4, 29-37*

[16]. Knorr, D. & Vogtmann, H. Quantity and quality determination of ecologically grown foods. 1983;

[17]. Schuphan, W. Effects of the application of inorganic and organic manures on the market quality and on the biological value of agricultural products. *Qualitas Plantarm et Materiae and Vegetabiles. xxi, 1972; 4, 381-398.*

[18]. Schuphan, W. Nutritional value of crops as influenced by organic and inorganic fertilizer treatments. quaitas plantarum - p1. *Foods and Human Nutrition xxiii, 1974; 4 . 333-358,*

[19]. Bremner, J. Determination of nitrogen in soil by the Kjeldhal method. *The Journal of Agricultural Science, 1960; 55(1): 11-33*

[20]. Olsen, S.R. Estimation of available phosphorus in soils by extraction with sodium bicarbonate. *The United States Department of Agriculture; Washington.1954;*

[21]. Walkley, A. & Black, I.A. An examination of the Degtjareff method for determining soil organic matter, and a proposed modification of the chromic acid titration method. Soil science, 1934; 37(1): 29-38.

[22]. Boka, B., Zewdu, A., Desie, G. Antioxidant properties of Ethiopian traditional bread (Injera) as affected by processing techniques and tef grain (Eragrostis tef (Zucc.)) varieties. *Canadian Chemical Transactions, 2013; 1(1).*

[23]. Yetneberk, S., Henriette L, D. K., Lloyd W. R., and John R. N. T. Effects of sorghum cultivar on injera quality, *Cereal Chemistry. 2004; 81(3):314–321*

[24]. Fox G, Nugusu Y, Nida H, Tedessa T, McLean G, and Jordan D. Evaluation of variation in Ethiopian sorghum injera quality with new imaging techniques. *Cereal Chem.2020; 97:362– 372. https ://doi.org/10.1002/cche.10252*

[25]. Associations of Analytical Chemistry (AOAC). Approved Official Method of Analysis of AOAC International, 2000; 2: 1.

[26]. American Associations of Cereal Chemistry (AACC). Approved Methods of the American Association of Cereal Chemists. AACC, 2000;

[27]. Vaintraub, I. A. &. Lapteva, N.A. Calorimetric determination of phytate in unpurified extracts of seeds and the products of their processing. *Analytical Biochemistry . 1988; I, 175: 227-230.*

[28]. Price, M.L., Scoyoc, S.V. & Butler, L.G. A critical evaluation of the vanillin reaction as an assay for tannin in sorghum grain. *Journal of Agricultural and Food Chemistry, 1978; 26 (5): 1214–1218*

[29]. Tilahun, G. Soil fertility status as influenced by different land uses in maybar areas of south wello zone, north Ethiopia. Haramaya University, unpublished document.2007;

[30]. Sánchez, P.A., Couto, W., & Buol, S.W. The fertility capability soil classification system: interpretation, applicability, and modification. *Geoderma, 1982; 27(4): p. 283-309.*

[31]. Abebe, M. The challenges and future prospects of soil chemistry in Ethiopia. Food and Agricultural Organizations.1996;

[**32].** Mirutse F., Haile. M., Kebede F.,Tsegay A., and Yamoah C. Response of Teff [Eragrostis (teff) Trotter] to Phosphorus and Nitrogen on Vertisol at North Ethiopia. *Journal of the drylands. 2009; 2(1): 8-14, 2009*

[33]. Gebreselassie, Y. Selected chemical and physical characteristics of soils of Adet research center and its testing sites in North-western Ethiopia. *Ethiopian Journal of Natural Resources. 2002;*

[34]. Kebede, L., Worku, S., Bultosa, G., & Yetnberk, S. Effect of extrusion operating conditions on the physical and sensory properties of tef (Eragrostis tef [Zucc.] Trotter) flour extrudates. *Ethiopian Journal of Applied Science and Technology,2010; 1(1), 27-38.*

[35]. Bultosa, G. Physicochemical characteristics of grain and flour in 13 tef [Eragrostis tef (Zucc.) Trotter] grain varieties. *Journal of. Applied Science Research, 2007; 3(12): 2042-2051.*

[36]. Simon,E., Simon, D., Miron, L. & Enache, G. The influence of organic fertilizers on the quality of the main harvest concerning the ecologically cropped corn. Lucrări Ştiinţifice , seria Agronomie, 2010; 33(6): 658-673.

[37]. Baye, K. Teff: Nutrient composition and health benefits. *International Food Policy Research Institute,2014; 67.*

[38]. Gebreab, T. Blending of teff (eragrostis teff) varieties with moringa (moringa oleifera) leaf powder to improve the nutritional content of injera. Haramaya university, haramaya, unpublished MSc thesis. 2015;

[39]. Abebe, Y., Hambidge, M., Stoecker, B., Bailey, K. & Gibson, R. Phytate, zinc, iron, and calcium content of selected raw and prepared foods consumed in rural Sidama, Southern Ethiopia, and implications for bioavailability. *Journal of Food Composition and Analysis, 2007; 20(3-4): 161-168.*

[40]. Kebede, Z. Levels of essential elements in three teffs [Eragrostis Tef (Zucc.) Trotter] Varieties. *Addis Ababa University.2009;*

[41]. Yetneberk, S., Rooney, L., & Talor, J. Improving the quality of sorghum injera by decortication and compositing with tef. Journal of the Science of Food and Agriculture, 2005; 85, 1252-1258.

[42]. Gebrekidan, B. & Gebrehiwot. B. Sorghum injera preparations and quality parameters in proceedings of the international symposium on sorghum grain quality. *International Crops Research Institute for the Semi-Arid Tropics) 1982; 21-31 October.*

[43]. Gebrehiwot, H.M., Shimels, H.A., Kirkman, K.P., Laing, M.D., & Manbaudhi, T. Nutritional and sensory evaluation of injera prepared from tef and Eragrostis curvula (Schrad.) Nees. flours with sorghum blends. *Front.Plant Science, 2016; 7., 1059*

[44]. Bloksma, A. Rheology of the breadmaking process. *Cereal Foods World,1990; 35, 228-236*

[45]. Yam, K.L. &Papadakis, S.E. A simple digital imaging method for measuring and analyzing the color of food surfaces. *Journal of food engineering, 2004; 61(1): 137-142.*

Click here to access/download;Figure;Cover letter.docx

Cover letter

Hayelom Berhe Dagnaw
Ethiopian Institute of Agricultural Research
Addis Ababa, Ethiopia

01-April 2022

Dear

We wish to submit an original research article entitled "Influence of Nitrogen Fertilizer Rate and Variety on Tef [Eragrostis tef (Zucc.) Trotter] Nutritional Composition and Sensory Quality of a Staple bread (Injera)" for consideration by [**PlOSONE**].

We confirm that this work is original and has not been published elsewhere, nor is it currently under consideration for publication elsewhere.

In this paper, we report on effects of different nitrogen fertilizer levels on the nutritional composition, sensory attributes of injera prepared and colours of injera from three different tef varieties in Ethiopia. This is significant because applications of chemical fertilizer are increased and some literature indicated that, excess chemical fertilizer have a significant effect on palatability and colour of food items.

We believe that this manuscript is appropriate for publication by PLOS ONE Journal of Food Sciences because today the agricultural practices associated with different chemical fertilizers become the big issue on the qualities of food items. Therefore, this manuscript also deals with such research results, that will be meet with the aims of the journal and we believe that this manuscript is appropriate for publication under this journal.

In Ethiopia tef is staple crop. And now a day the injera, Ethiopian traditional leavened bread's quality becomes a big issue. From the cultural aspects, the society complains that the sensory attributes and colour of injera are significantly varied. And some literatures reported that excess N fertilizer have effects on palatability of food items. Therefore, this paper mainly proofs that excess N fertilizes with tef varieties interaction on the sensory qualities and the colour of injera; and the study indicated that, did not have much significant effects, rather than, this paper reports that only nitrogen fertilizer did not have a significant effect on the sensorial attributes and the colour of injera, but this may be happened due to the interactions of soil microbial and fermentation kinetics of the dough. Therefore, from my point of view, this research paper is directly related with the food science aspects, and is important to publish under the PLOS ONE Journal.

We have no conflicts of interest to disclose.
Please address all correspondence concerning this manuscript to me at hayelom210@gmail.com
Thank you for your consideration of this manuscript.

Sincerely
Hayelom Berhe Dagnaw
