# Supplementary material for: Influence of nitrogen fertilizer rate and variety on tef [Eragrostis tef (Zucc.) Trotter] nutritional composition and sensory quality of a staple bread (Injera)
Source: PLoS One. 2024 Jan 2;19(1):e0295491. doi: 10.1371/journal.pone.0295491 (PMC10760880; doi:10.1371/journal.pone.0295491)
Supplement: Attachment — Submitted filename: Reviewer Recommendation and Comments for manuscript number PONE-D-22-09743.docx [file pone.0295491.s001.docx]

**Reviewer Recommendation and comments and Comments for manuscript number PONE-D-22-09743**

**Recommendation: Major Revision**

The survey reports an interesting topic that points out the influence of nitrogen fertilizer rate and variety on Tef [*Eragrostis tef (Zucc.) Trotter*] nutritional composition and sensory quality of a staple bread (injera). Despite that the study didn’t detected significant improvements with increasing N rates; however, scientifically, reporting these findings is very important and crucial. The manuscript presents a huge gap in its standard English; accordingly, I suggest passing it to a native English speaker for an extended editing. The Financial Disclosure and Ethics Statement should be adjusted according to the journal’s guidelines.

The Abstract part should be re-written due to an extensive English language issue. The majority of the keywords fit. The Introduction part is badly written in standard English; accordingly, it should be reformulated in a better language style. The Materials and methods part shows numerous needed adjustments in terms of sentences standard English formulation. Moreover, most methods especially related to laboratory analysis lack explanation, therefore they should be added. The Results and discussion part needs major adjustments mainly related to the scientific analysis of obtained findings. However, the statistical approach adopted is correctly performed. On the other hand, I suggest a major revision or re-writing this whole part as it shows uncountable linguistic mistakes and the need for a wide range of sentences reformulation. Regarding the Conclusions part, it summarizes the study’s findings and suggests further research. Only minor adjustments are required related to sentences reformulation.

Based on the above and below detailed explanation, recommendations and suggestions, I think that the manuscript shows a certain merit to be published in “PLoS One” once all suggestions and recommendations are fully taken into consideration and well addressed.

**Financial Disclosure**

1) Kindly remove the unnecessary information in the disclosure’s statement (“The first… Adidas Ababa, Ethiopia”).

**Ethics statement**

1) Kindly remove the current statement written and replace it by “N/A”.

**Abstract**

1) Lines 12–30: The Abstract part should be re-written due to an extensive English language issue. The majority of the keywords fit.
2) Line 13: Kindly remove the comma “,” after “that”.
3) Line 14: Kindly adjust as follow: “Nowadays”.
4) Lines 14–15: “Nowadays… (N)”: The sentence is badly written in standard English; accordingly, kindly reformulate it.
5) Line 17: Kindly adjust as follow: “of injera from three…”
6) Lines 15–18: “Therefore… DAP”: The sentence is long; accordingly, kindly reformulate in order to make it more concise, clearer and more aiming.
7) Line 20: Kindly adjust as follow: “were determined by panellists”.
8) Lines 20–21: Kindly adjust the sentence as follow: “Results showed that only protein…”
9) Line 21: Kindly remove “was” and “is”.
10) Lines 22–23: “Varieties… content”: Kindly remove this sentence as reporting non significant findings is inappropriate from the scientific point of view.
11) Line 23: Kindly remove “Using panellists”.
12) Lines 23–24: “Kora… rates”: The sentence is badly written in standard English; accordingly, kindly reformulate it.
13) Lines 24–25: “Grand… while”: Kindly remove this sentence as reporting non significant findings is inappropriate from the scientific point of view.
14) Lines 25–26: “Asgori… color”: The sentence is badly written in standard English; accordingly, kindly reformulate it.
15) Line 27: Kindly remove the comma “,” after “that” and remove “On average means of varieties”.
16) Line 28: Kindly adjust the sentence as follow: “Kora had the highest…” and remove “with lower L*”.
17) Lines 28–29: “Kora… quality”: The sentence is badly written in standard English; accordingly, kindly reformulate it.
18) Lines 29–30: “Injera… rate”: Same recommendation as in the previous comment.
19) Line 31: The majority of keywords fit well but kindly remove “and CIEL * a* b values” and adjust the last keyword as follow: “color”.

**1. Introduction**

1) Lines 38–83: The Introduction part is badly written in standard English; accordingly, it should be reformulated in a better language style.
2) Lines 38–39: “Tef… Ethiopia”: The sentence is badly written in standard English; accordingly, kindly reformulate it.
3) Lines 38–39: Kindly adjust the in-text references form as follow: “[1–4]”.
4) Lines 39–40: “Tef… cultivars”: The sentence is badly written in standard English; accordingly, kindly reformulate it.
5) Lines 40–43: “Tef… [5,6]”: The sentence is somehow heavy and long; accordingly, kindly reformulate in order to make it more concise, clearer and more aiming.
6) Lines 44–46: “Tef… [7,8,9]”: The sentence is badly written in standard English; accordingly, kindly reformulate it. Moreover, kindly adjust the in-text references form as follow: “[7–9]”.
7) Lines 47–49: “In Ethiopia… [11]”: The sentence is badly written in standard English; accordingly, kindly reformulate it.
8) Lines 51–54: “Application… damage”: Same recommendation as in the previous comment.
9) Lines 57–58: “The application… enhancement”: This statement lacks reliable sources (references); accordingly, kindly provide them.
10) Lines 60–64: “Moreover… nutrients”: Same recommendation as in the previous comment.
11) Line 66: “Nitrogen… practices”: Same recommendation as in the previous two comments.
12) Lines 66–68: “But… [16]”: The sentence is badly written in standard English; accordingly, kindly reformulate it.
13) Line 69: Kindly adjust as follow: “Gu et al. [13]”.
14) Lines 69–75: “Gu… [13]”: These sentences are badly written in standard English; accordingly, kindly reformulate them.
15) Lines 76–79: “However… injera”: Same recommendation as in the previous comment.
16) Lines 80–81: “The reason… production”: Kindly remove these sentences.

**2. Materials and methods**

1) Lines 85–158: The Materials and methods part shows numerous needed adjustments in terms of sentences standard English formulation. Moreover, most methods especially related to laboratory analysis lack explanation, therefore they should be added.
2) Study area, lines 86–87: “The study… 2017”: The sentence is badly written in standard English; accordingly, kindly reformulate it.
3) Study area, lines 88–91: “The experimental… 832”: Same recommendation as in the previous comment. Moreover, kindly adjust as follow: “832 mm”.
4) Soil sample characterization, lines 93–95: Kindly explain briefly the adopted methods.
5) Injera preparations, line 115: Kindly adjust as follow: “were mixed”.
6) Injera preparations, lines 115–117: “Therefore… minutes”: The sentence is long and cumbersome; accordingly, kindly reformulate in order to make it more concise, clearer and more aiming.
7) Injera preparations, line 118: Kindly replace “for” by “at” and adjust as follow: “were added” and “28–30°C”.
8) Injera preparations, line 119: Kindly adjust the sentence as follow: “for 2–3 hours at 28–30°C and left until…”
9) Injera preparations, lines 119–120: Kindly remove “After… formed” and adjust the sentence as follow: “After that, around 500 ml of the batter were poured…”
10) Injera preparations, lines 122–123: “Then… obtained”: The sentence is badly written in standard English; accordingly, kindly reformulate it.
11) Image analysis, line 128: Kindly adjust as follow: “45°C”.
12) Image analysis, lines 128–129: “The room… injera”: The sentence is badly written in standard English; accordingly, kindly reformulate it.
13) Image analysis, lines 129–130: “A camera… used”: Kindly remove this sentence.
14) Image analysis, line 132: Kindly replace “with no” by “without”.
15) Image analysis, line 134: Kindly adjust as follow: “were measured”.
16) Image analysis, line 135: Kindly adjust as follow: “using the same software”.
17) Image analysis, lines 136–137: “Two… samples”: The sentence is badly written in standard English; accordingly, kindly reformulate it.
18) Laboratory analysis, line 143: Kindly explain briefly the method of determination of each element.
19) Laboratory analysis, lines 149–152: Same recommendation as in the previous comment.
20) Statistical analysis, line 156: Kindly remove “And”.
21) Statistical analysis, line 157: Kindly replace “by” by “using”.
22) Statistical analysis, line 157: Kindly replace “using” by “following”.
23) Statistical analysis, lines 156–158: “The relationship… 0.05”: The sentence is long and cumbersome; accordingly, kindly reformulate it.

**3. Results and discussion**

1) Lines 160–525: The Results and discussion part needs major adjustments mainly related to the scientific analysis of obtained findings. However, the statistical approach adopted is correctly performed. On the other hand, I suggest a major revision or re-writing this whole part as it shows uncountable linguistic mistakes and the need for a wide range of sentences reformulation.
2) Line 159: Kindly adjust this part’s title as follow: “Results and discussion”.
3) Soil physicochemical properties of the experimental site, lines 161–162: Kindly remove the “( )”.
4) Soil physicochemical properties of the experimental site, lines 162–163: “Soil… method”: Kindly move this sentence to the Materials and methods part.
5) Soil physicochemical properties of the experimental site, lines 161–172: I don’t see the need to discuss this table !! Also, it should be moved to the Materials and methods part as it has no place in the current part.
6) Nutritional Compositions of Tef Grain as Influenced by Nitrogen Fertilizer Rates and Varieties, Moisture content, lines 185–186: “The result… (Table 2)”: Kindly remove this sentence as analyzing non significant findings are inappropriate from the scientific point of view.
7) Nutritional Compositions of Tef Grain as Influenced by Nitrogen Fertilizer Rates and Varieties, Moisture content, lines 189–191: “And… rates”: Same recommendation as in the previous comment.
8) Nutritional Compositions of Tef Grain as Influenced by Nitrogen Fertilizer Rates and Varieties, Moisture content, line 192: Kindly mention that it was an increase and mention the percentage of increase.
9) Nutritional Compositions of Tef Grain as Influenced by Nitrogen Fertilizer Rates and Varieties, Ash content of Tef grain, line 193: Kindly adjust the title of this paragraph as follow: “Ash content of Tef grain”.
10) Nutritional Compositions of Tef Grain as Influenced by Nitrogen Fertilizer Rates and Varieties, Ash content of Tef grain, line 194: “Ash… minerals”: Kindly remove this sentence.
11) Nutritional Compositions of Tef Grain as Influenced by Nitrogen Fertilizer Rates and Varieties, Ash content of Tef grain, lines 194–196: “Both… [35]”: Kindly remove this sentence as analyzing non significant findings is inappropriate from the scientific point of view.
12) Nutritional Compositions of Tef Grain as Influenced by Nitrogen Fertilizer Rates and Varieties, Ash content of Tef grain, line 196: Kindly remove “On the other hand” and adjust the sentence as follow: “Bultosa [35] obtained…”
13) Nutritional Compositions of Tef Grain as Influenced by Nitrogen Fertilizer Rates and Varieties, Ash content of Tef grain, lines 199–200: “And… (Table 2)”: Kindly remove this sentence as analyzing non significant findings is inappropriate from the scientific point of view.
14) Nutritional Compositions of Tef Grain as Influenced by Nitrogen Fertilizer Rates and Varieties, Protein content of grain, line 201: Kindly adjust this paragraph’s title as follow: “Protein content of grain”.
15) Nutritional Compositions of Tef Grain as Influenced by Nitrogen Fertilizer Rates and Varieties, Protein content of grain, lines 202–203: “The result… (Table 1)”: The sentence is badly written in standard English; accordingly, kindly reformulate it.
16) Nutritional Compositions of Tef Grain as Influenced by Nitrogen Fertilizer Rates and Varieties, Protein content of grain, lines 204–205: Kindly remove “was” and adjust the sentence as follow: “ranged between 9.51 and 11.14% being comparable to the findings of Bultosa [35] (8.7–11%)”.
17) Nutritional Compositions of Tef Grain as Influenced by Nitrogen Fertilizer Rates and Varieties, Protein content of grain, line 205: Kindly adjust as follow: “is expected”.
18) Nutritional Compositions of Tef Grain as Influenced by Nitrogen Fertilizer Rates and Varieties, Protein content of grain, lines 205–206: “The increase… proteins”: This statement lacks reliable sources (references); accordingly, kindly provide them.
19) Nutritional Compositions of Tef Grain as Influenced by Nitrogen Fertilizer Rates and Varieties, Protein content of grain, lines 206–207: “When we… (Table)”: Kindly remove this sentence.
20) Nutritional Compositions of Tef Grain as Influenced by Nitrogen Fertilizer Rates and Varieties, Protein content of grain, line 207: Kindly adjust as follow: “This indicates… may have significant…”
21) Nutritional Compositions of Tef Grain as Influenced by Nitrogen Fertilizer Rates and Varieties, Crude fat content, lines 217–218: “the ranged… 3.0%”: The sentence is badly written in standard English; accordingly, kindly reformulate it.
22) Nutritional Compositions of Tef Grain as Influenced by Nitrogen Fertilizer Rates and Varieties, Crude fat content, line 218: Kindly adjust as follow: “accumulation’.
23) Nutritional Compositions of Tef Grain as Influenced by Nitrogen Fertilizer Rates and Varieties, Crude fat content, lines 218–219: Kindly remove “and have not shown significant differences”.
24) Nutritional Compositions of Tef Grain as Influenced by Nitrogen Fertilizer Rates and Varieties, Crude fat content, lines 218–221: “The fat… [36]: The sentence is badly written in standard English; accordingly, kindly reformulate it.
25) Nutritional Compositions of Tef Grain as Influenced by Nitrogen Fertilizer Rates and Varieties, Crude fat content, lines 221–223: “And… varieties”: Kindly remove this sentence.
26) Nutritional Compositions of Tef Grain as Influenced by Nitrogen Fertilizer Rates and Varieties, Crude fiber, lines 225–226: “The crude… varieties”: Kindly remove this sentence as analyzing non significant findings is inappropriate from the scientific point of view.
27) Nutritional Compositions of Tef Grain as Influenced by Nitrogen Fertilizer Rates and Varieties, Crude fiber, line 226: Kindly remove “And”.
28) Nutritional Compositions of Tef Grain as Influenced by Nitrogen Fertilizer Rates and Varieties, Crude fiber, line 227: Kindly adjust as follow: “with the report of Baye [37]”.
29) Nutritional Compositions of Tef Grain as Influenced by Nitrogen Fertilizer Rates and Varieties, Crude fiber, lines 227–229: “Both… varieties”: Kindly remove this sentence as analyzing non significant findings is inappropriate from the scientific point of view.
30) Nutritional Compositions of Tef Grain as Influenced by Nitrogen Fertilizer Rates and Varieties, Total carbohydrate and energy content, lines 231–232: “but… rates”: Same recommendation as in the previous comment.
31) Nutritional Compositions of Tef Grain as Influenced by Nitrogen Fertilizer Rates and Varieties, Total carbohydrate and energy content, line 233: Kindly adjust as follow: “had” and remove “is”.
32) Nutritional Compositions of Tef Grain as Influenced by Nitrogen Fertilizer Rates and Varieties, Total carbohydrate and energy content, line 234: Kindly adjust as follow: “increased” and remove “was”.
33) Nutritional Compositions of Tef Grain as Influenced by Nitrogen Fertilizer Rates and Varieties, Total carbohydrate and energy content, line 235: Kindly adjust as follow: “content”.
34) Nutritional Compositions of Tef Grain as Influenced by Nitrogen Fertilizer Rates and Varieties, Total carbohydrate and energy content, lines 235–237: “But… grains”: Kindly remove this sentence as analyzing non significant findings is inappropriate from the scientific point of view.
35) Effects of Nitrogen Fertilizer Rates and Varieties on the Anti-nutritional Factor of Tef Phytic acid content, lines 240–241: “The interaction… grain”: The sentence is badly written in standard English; accordingly, kindly reformulate it.
36) Effects of Nitrogen Fertilizer Rates and Varieties on the Anti-nutritional Factor of Tef Phytic acid content, line 243: Kindly adjust the sentence as follow: “According to Baye [37]…”
37) Effects of Nitrogen Fertilizer Rates and Varieties on the Anti-nutritional Factor of Tef Phytic acid content, lines 244–245: Kindly adjust as follow: “ranged between 682 and 1374… being higher than the present findings…”
38) Effects of Nitrogen Fertilizer Rates and Varieties on the Anti-nutritional Factor of Tef Phytic acid content, lines 243–246: “According… contents”: The sentence is long and cumbersome; accordingly, kindly reformulate in order to make it more concise, clearer and more aiming.
39) Effects of Nitrogen Fertilizer Rates and Varieties on the Anti-nutritional Factor of Tef Phytic acid content, lines 246–248: “On the other… grains”: Kindly remove this sentence as analyzing non significant findings is inappropriate from the scientific point of view.
40) Effects of Nitrogen Fertilizer Rates and Varieties on the Anti-nutritional Factor of Tef Phytic acid content, Tannin content of Tef grain, line 260: Kindly adjust this paragraph’s title as follow: “Tannin content of Tef grain”.
41) Effects of Nitrogen Fertilizer Rates and Varieties on the Anti-nutritional Factor of Tef Phytic acid content, Tannin content of Tef grain, line 261: Kindly remove “only”.
42) Effects of Nitrogen Fertilizer Rates and Varieties on the Anti-nutritional Factor of Tef Phytic acid content, Tannin content of Tef grain, line 262: Higher compared to what ? Kindly mention.
43) Effects of Nitrogen Fertilizer Rates and Varieties on the Anti-nutritional Factor of Tef Phytic acid content, Tannin content of Tef grain, line 264: Kindly adjust as follow: “to a report of Baye [37]”.
44) Effects of Nitrogen Fertilizer Rates and Varieties on the Anti-nutritional Factor of Tef Phytic acid content, Tannin content of Tef grain, lines 266–267: “Tannin… (Table 3)”: The sentence is badly written in standard English; accordingly, kindly reformulate it.
45) Effects of Nitrogen Fertilizer Rates on Mineral Contents of Tef Grain Iron (Fe), line 272: Kindly replace “to” by “and”.
46) Effects of Nitrogen Fertilizer Rates on Mineral Contents of Tef Grain Iron (Fe), line 273: Kindly remove “was found” and adjust as follow “with those of Baye [37]”.
47) Effects of Nitrogen Fertilizer Rates on Mineral Contents of Tef Grain Iron (Fe), lines 273–274: Kindly adjust as follow: “where the ranges of Fe…”
48) Effects of Nitrogen Fertilizer Rates on Mineral Contents of Tef Grain Iron (Fe), line 274: Kindly add the unit.
49) Effects of Nitrogen Fertilizer Rates on Mineral Contents of Tef Grain Iron (Fe), lines 273–276: “The present… varieties”: The sentence is long and badly written in standard English; accordingly, kindly reformulate in order to make it more concise, clearer and more aiming.
50) Effects of Nitrogen Fertilizer Rates on Mineral Contents of Tef Grain Iron (Fe), lines 276–277: “These… [39]”: The sentence is badly written in standard English; accordingly, kindly reformulate it.
51) Effects of Nitrogen Fertilizer Rates on Mineral Contents of Tef Grain Iron (Fe), line 277: Kindly remove “to this”.
52) Effects of Nitrogen Fertilizer Rates on Mineral Contents of Tef Grain Iron (Fe), lines 277–278: “In addition… contents”: This statement lack reliable sources (references); accordingly, kindly provide them.
53) Effects of Nitrogen Fertilizer Rates on Mineral Contents of Tef Grain Iron (Fe), Calcium (Ca), line 293: Kindly replace “have” by “had”.
54) Effects of Nitrogen Fertilizer Rates on Mineral Contents of Tef Grain Iron (Fe), Calcium (Ca), line 295: Kindly adjust as follow: “contents”.
55) Effects of Nitrogen Fertilizer Rates on Mineral Contents of Tef Grain Iron (Fe), Calcium (Ca), lines 297–299: “The ranges… [37]”: The sentence is badly written in standard English; accordingly, kindly reformulate it.
56) Effects of Nitrogen Fertilizer Rates on Mineral Contents of Tef Grain Iron (Fe), Calcium (Ca), lines 293–299: The scientific analysis of this section needs to be adjusted; accordingly, kindly reformulate it in a proper manner.
57) Effects of Nitrogen Fertilizer Rates on Mineral Contents of Tef Grain Iron (Fe), Calcium (Ca), line 300: Kindly replace “from” by “with”.
58) Effects of Nitrogen Fertilizer Rates on Mineral Contents of Tef Grain Iron (Fe), Calcium (Ca), lines 299–301: “Regardless… Asgori”: The sentence is badly written in standard English; accordingly, kindly reformulate it.
59) Effects of Nitrogen Fertilizer Rates on Mineral Contents of Tef Grain Iron (Fe), Calcium (Ca), line 302: Kindly replace “that” by “where” and adjust as follow: “had higher Ca compared to…”. Also, kindly mention of increase or decrease.
60) Effects of Nitrogen Fertilizer Rates on Mineral Contents of Tef Grain Iron (Fe), Calcium (Ca), lines 303–304: “From… (Table 4)”: Kindly remove this sentence as analyzing non significant findings is inappropriate from the scientific point of view.
61) Effects of Nitrogen Fertilizer Rates on Mineral Contents of Tef Grain Iron (Fe), Phosphorus content of Tef grain, lines 307–310: “As reported… (Table 4)”: The sentence is long, cumbersome and badly written in standard English; accordingly, kindly reformulate in order to make it more concise, clearer and more aiming.
62) Effects of Nitrogen Fertilizer Rates on Mineral Contents of Tef Grain Iron (Fe), Phosphorus content of Tef grain, line 311: Kindly adjust as follow: “fertilizers”.
63) Effects of Nitrogen Fertilizer Rates on Mineral Contents of Tef Grain Iron (Fe), Phosphorus content of Tef grain, line 313: Kindly remove “slightly”.
64) Effects of Nitrogen Fertilizer Rates and Tef Varieties on Sensory Quality of Injera, line 314: Kindly adjust the paragraph’s title as follow: “Effects of Nitrogen Fertilizer Rates and Tef Varieties on Sensory Quality of Injera”.
65) Effects of Nitrogen Fertilizer Rates and Tef Varieties on Sensory Quality of Injera, line 316: Kindly remove “( )”.
66) Effects of Nitrogen Fertilizer Rates and Tef Varieties on Sensory Quality of Injera, line 317: Kindly adjust as follow: “was baked”.
67) Effects of Nitrogen Fertilizer Rates and Tef Varieties on Sensory Quality of Injera, Sensory evaluation of Injera, line 319: Kindly adjust this paragraph’s title as follow: “Sensory evaluation of Injera”.
68) Effects of Nitrogen Fertilizer Rates and Tef Varieties on Sensory Quality of Injera, Sensory evaluation of Injera, Color, line 327: Kindly adjust as follow: “maximal”.
69) Effects of Nitrogen Fertilizer Rates and Tef Varieties on Sensory Quality of Injera, Sensory evaluation of Injera, Color, lines 326–329: “Average… use”: The sentence is long and cumbersome; accordingly, kindly reformulate in order to make it more concise, clearer and more aiming.
70) Effects of Nitrogen Fertilizer Rates and Tef Varieties on Sensory Quality of Injera, Sensory evaluation of Injera, Color, lines 331–334: “Injera… [42]”: These sentences are badly written in standard English; accordingly, kindly reformulate them.
71) Effects of Nitrogen Fertilizer Rates and Tef Varieties on Sensory Quality of Injera, Sensory evaluation of Injera, Flavor, lines 337–338: Kindly remove “but not have increasing or decreasing trends” and adjust as follow: “with Gu et al. [13] who reported…”
72) Effects of Nitrogen Fertilizer Rates and Tef Varieties on Sensory Quality of Injera, Sensory evaluation of Injera, Flavor, lines 340–342: “This may… evaluations”: The sentence is badly written in standard English; accordingly, kindly reformulate it.
73) Effects of Nitrogen Fertilizer Rates and Tef Varieties on Sensory Quality of Injera, Sensory evaluation of Injera, Flavor, lines 342–343: “Average… injera”: Kindly remove this sentence as analyzing non significant findings is inappropriate from the scientific point of view.
74) Effects of Nitrogen Fertilizer Rates and Tef Varieties on Sensory Quality of Injera, Sensory evaluation of Injera, Texture, lines 348–349: “Texture… softness”: The sentence is badly written in standard English; accordingly, kindly reformulate it.
75) Effects of Nitrogen Fertilizer Rates and Tef Varieties on Sensory Quality of Injera, Sensory evaluation of Injera, Texture, lines 349–350: “Injera… [42]”: Same recommendation as in the previous comment.
76) Effects of Nitrogen Fertilizer Rates and Tef Varieties on Sensory Quality of Injera, Sensory evaluation of Injera, Texture, line 356: Kindly adjust as follow: “with the present study”.
77) Effects of Nitrogen Fertilizer Rates and Tef Varieties on Sensory Quality of Injera, Sensory evaluation of Injera, Taste, lines 358–360: “From… dislike”: The sentence is badly written in standard English; accordingly, kindly reformulate it.
78) Effects of Nitrogen Fertilizer Rates and Tef Varieties on Sensory Quality of Injera, Sensory evaluation of Injera, Taste, lines 360–363: “The sensory… excellent”: The sentence is long, cumbersome and badly written in standard English; accordingly, kindly reformulate in order to make it more concise, clearer and more aiming.
79) Effects of Nitrogen Fertilizer Rates and Tef Varieties on Sensory Quality of Injera, Sensory evaluation of Injera, Taste, lines 363–365: “And… injera”: Same recommendation as in the previous comment.
80) Effects of Nitrogen Fertilizer Rates and Tef Varieties on Sensory Quality of Injera, Sensory evaluation of Injera, Taste, lines 366–369: “Mean… (Table 5)”: Same recommendation as in the previous two comments.
81) Effects of Nitrogen Fertilizer Rates and Tef Varieties on Sensory Quality of Injera, Sensory evaluation of Injera, Folding capacity (FC), lines 372–374: “However… slightly”: Same recommendation as in the previous comments.
82) Effects of Nitrogen Fertilizer Rates and Tef Varieties on Sensory Quality of Injera, Sensory evaluation of Injera, Eye size (ES) and eye distribution (ED) of injera, lines 382–385: “From… slightly”: Same recommendation as in the previous comments.
83) Effects of Nitrogen Fertilizer Rates and Tef Varieties on Sensory Quality of Injera, Sensory evaluation of Injera, Eye size (ES) and eye distribution (ED) of injera, lines 386–388: “This… injera”: Same recommendation as in the previous comments.
84) Effects of Nitrogen Fertilizer Rates and Tef Varieties on Sensory Quality of Injera, Sensory evaluation of Injera, Eye size (ES) and eye distribution (ED) of injera, line 397: Kindly adjust as follow: “affect”.
85) Effects of Nitrogen Fertilizer Rates and Tef Varieties on Sensory Quality of Injera, Sensory evaluation of Injera, Top and bottom surfaces (TBS) of injera, lines 401–402: Kindly adjust as follow: “were rated”.
86) Effects of Nitrogen Fertilizer Rates and Tef Varieties on Sensory Quality of Injera, Sensory evaluation of Injera, Top and bottom surfaces (TBS) of injera, lines 402–403: Kindly adjust the in-text reference form according to the journal’s guidelines.
87) Effects of Nitrogen Fertilizer Rates and Tef Varieties on Sensory Quality of Injera, Sensory evaluation of Injera, Top and bottom surfaces (TBS) of injera, lines 402–405: “The study… varieties”: The sentence is badly written in standard English; accordingly, kindly reformulate it.
88) Effects of Nitrogen Fertilizer Rates and Tef Varieties on Sensory Quality of Injera, Sensory evaluation of Injera, Overall acceptability of injera quality, lines 413–414: “Overall… product”: Same recommendation as in the previous comment.
89) Effects of Nitrogen Fertilizer Rates and Tef Varieties on Sensory Quality of Injera, Sensory evaluation of Injera, Overall acceptability of injera quality, line 419: Kindly remove “And”.
90) Effects of Nitrogen Fertilizer Rates and Tef Varieties on Sensory Quality of Injera, Sensory evaluation of Injera, Overall acceptability of injera quality, line 420: It is a significant difference !! You cannot say “minor” !!
91) Effects of Nitrogen Fertilizer Rates and Tef Varieties on Sensory Quality of Injera, Sensory evaluation of Injera, Overall acceptability of injera quality, lines 419–424: “Injera… present”: These sentences are badly written in standard English; accordingly, kindly reformulate them.
92) Effects of Nitrogen Fertilizer Rates and Tef Varieties on Sensory Quality of Injera, Sensory evaluation of Injera, Total number of holes and filtered eyes of injera, line 430: Kindly remove “( )”.
93) Effects of Nitrogen Fertilizer Rates and Tef Varieties on Sensory Quality of Injera, Sensory evaluation of Injera, Total number of holes and filtered eyes of injera, Number of holes of injera, lines 432–434: “A number… (Table 6)”: The sentence is badly written in standard English; accordingly, kindly reformulate it.
94) Effects of Nitrogen Fertilizer Rates and Tef Varieties on Sensory Quality of Injera, Sensory evaluation of Injera, Total number of holes and filtered eyes of injera, Number of holes of injera, line 434: Kindly adjust as follow: “may be due”.
95) Effects of Nitrogen Fertilizer Rates and Tef Varieties on Sensory Quality of Injera, Sensory evaluation of Injera, Total number of holes and filtered eyes of injera, Number of holes of injera, lines 435–438: “Thus… software”: The sentence is long and cumbersome; accordingly, kindly reformulate in order to make it more concise, clearer and more aiming.
96) Effects of Nitrogen Fertilizer Rates and Tef Varieties on Sensory Quality of Injera, Sensory evaluation of Injera, Total number of holes and filtered eyes of injera, Filtered eyes of injera, lines 449–457: “For… respectively”: These sentences are badly written in standard English; accordingly, kindly reformulate them.
97) Effects of Nitrogen Fertilizer Rates and Tef Varieties on Sensory Quality of Injera, Sensory evaluation of Injera, Total number of holes and filtered eyes of injera, Filtered eyes of injera, line 462: Kindly adjust as follow: “was obtained”.
98) Effects of Nitrogen Fertilizer Rates and Tef Varieties on Sensory Quality of Injera, Sensory evaluation of Injera, Total number of holes and filtered eyes of injera, Filtered eyes of injera, lines 463–467: “Based… investigations”: The sentence is long and badly written in standard English; accordingly, kindly reformulate in order to make it more concise, clearer and more aiming.
99) Effects of Nitrogen Fertilizer Rates and Tef Varieties on Sensory Quality of Injera, Colors (CIE L* a* b*) values of injera, Lightness (L) color of injera, lines 471–473: “The more… injera”: The sentence is badly written in standard English; accordingly, kindly reformulate it.
100) Effects of Nitrogen Fertilizer Rates and Tef Varieties on Sensory Quality of Injera, Colors (CIE L* a* b*) values of injera, Lightness (L) color of injera, lines 474–475: “All… [45]”: Same recommendation as in the previous comment.
101) Effects of Nitrogen Fertilizer Rates and Tef Varieties on Sensory Quality of Injera, Colors (CIE L* a* b*) values of injera, Lightness (L) color of injera, lines 476–483: “Interactions… (Table 6)”: Same recommendation as in the previous two comments.
102) Effects of Nitrogen Fertilizer Rates and Tef Varieties on Sensory Quality of Injera, Colors (CIE L* a* b*) values of injera, Redness (a*) color of injera, lines 485–489: “The analysis… values”: Same recommendation as in the previous comments.
103) Effects of Nitrogen Fertilizer Rates and Tef Varieties on Sensory Quality of Injera, Colors (CIE L* a* b*) values of injera, Redness (a*) color of injera, line 492: Kindly replace “from” by “for”.
104) Effects of Nitrogen Fertilizer Rates and Tef Varieties on Sensory Quality of Injera, Colors (CIE L* a* b*) values of injera, Redness (a*) color of injera, lines 494–497: “This… variety”: These sentences are badly written in standard English; accordingly, kindly reformulate them.
105) Effects of Nitrogen Fertilizer Rates and Tef Varieties on Sensory Quality of Injera, Colors (CIE L* a* b*) values of injera, Yellowness (b*) color of injera, lines 514–518: “This… respectively”: Same recommendation as in the previous comment.
106) Effects of Nitrogen Fertilizer Rates and Tef Varieties on Sensory Quality of Injera, Colors (CIE L* a* b*) values of injera, Yellowness (b*) color of injera, lines 520–521: “This… varieties”: Same recommendation as in the previous two comments.

**4. Conclusions**

1) Lines 527–544: Regarding the Conclusions part, it summarizes the study’s findings and suggests further research. Only minor adjustments are required related to sentences reformulation.
2) Lines 527–529: “The study… decreased”: The sentence is heavy; accordingly, kindly reformulate in order to make it clearer and more aiming.
3) Lines 530–531: “The subjective… effects”: Kindly remove this sentence as in the Conclusions part, we only point out the significant findings.
4) Lines 532–533: Kindly remove “but slightly decreased with N rates”.
5) Lines 537–540: “Grand… injera”: Kindly remove these sentences as in the Conclusions part, we only point out the significant findings.
6) Lines 540–541: “In general… differences”: The sentence is badly written in standard English; accordingly, kindly reformulate it.
7) Lines 541–544: “Based… dough”: The sentence is long, cumbersome and heavy; accordingly, kindly reformulate in order to make it more concise, clearer and more aiming.
