# Supplementary material for: Influence of nitrogen fertilizer rate and variety on tef [Eragrostis tef (Zucc.) Trotter] nutritional composition and sensory quality of a staple bread (Injera)
Source: PLoS One. 2024 Jan 2;19(1):e0295491. doi: 10.1371/journal.pone.0295491 (PMC10760880; doi:10.1371/journal.pone.0295491)
Supplement: Attachment — Submitted filename: Response to revivers letter.docx [file pone.0295491.s003.docx]

**Reviewer Recommendation and comments and Comments for manuscript number PONE-D-22-09743**

**Recommendation: Major Revision**

The survey reports an interesting topic that points out the influence of nitrogen fertilizer rate and variety on Tef [*Eragrostis tef (Zucc.) Trotter*] nutritional composition and sensory quality of a staple bread (injera). Despite that the study didn’t detected significant improvements with increasing N rates; however, scientifically, reporting these findings is very important and crucial. The manuscript presents a huge gap in its standard English; accordingly, I suggest passing it to a native English speaker for an extended editing. The Financial Disclosure and Ethics Statement should be adjusted according to the journal’s guidelines.

The Abstract part should be re-written due to an extensive English language issue. The majority of the keywords fit. The Introduction part is badly written in standard English; accordingly, it should be reformulated in a better language style. The Materials and methods part shows numerous needed adjustments in terms of sentences standard English formulation. Moreover, most methods especially related to laboratory analysis lack explanation, therefore they should be added. The Results and discussion part needs major adjustments mainly related to the scientific analysis of obtained findings. However, the statistical approach adopted is correctly performed. On the other hand, I suggest a major revision or re-writing this whole part as it shows uncountable linguistic mistakes and the need for a wide range of sentences reformulation. Regarding the Conclusions part, it summarizes the study’s findings and suggests further research. Only minor adjustments are required related to sentences reformulation.

Based on the above and below detailed explanation, recommendations and suggestions, I think that the manuscript shows a certain merit to be published in “PLoS One” once all suggestions and recommendations are fully taken into consideration and well addressed.

**Financial Disclosure**

1) Kindly remove the unnecessary information in the disclosure’s statement (“The first… Adidas Ababa, Ethiopia”).

Answer: all are adjusted and accepted

**Ethics statement**

1) Kindly remove the current statement written and replace it by “N/A”.

Answer: all are adjusted and accepted

**Abstract**

1) Lines 12–30: The Abstract part should be re-written due to an extensive English language issue. The majority of the keywords fit.

Answer: abstract was revised as much as the author can

2) Line 13: Kindly remove the comma “,” after “that”.

Answer: comma was removed,

3) Line 14: Kindly adjust as follow: “Nowadays”.

Answer: it was adjusted and replaced

4) Lines 14–15: “Nowadays… (N)”: The sentence is badly written in Standard English; accordingly, kindly reformulate it.

Answer: the author tries to adjust and reformulate the sentences

5) Line 17: Kindly adjust as follow: “of injera from three…”

Answer: It was accepted and adjusted as recommended

6) Lines 15–18: “Therefore… DAP”: The sentence is long; accordingly, kindly reformulate in order to make it more concise, clearer and more aiming.

Answer: the author reformulated the recommended sentences

7) Line 20: Kindly adjust as follow: “were determined by panellists”.

Answer: the phrases was adjusted as recommended one

8) Lines 20–21: Kindly adjust the sentence as follow: “Results showed that only protein…”

Answer: the author was the recommended sentences

9) Line 21: Kindly remove “was” and “is”.

Answer: the sentences was removed

10) Lines 22–23: “Varieties… content”: Kindly remove this sentence as reporting non significant findings is inappropriate from the scientific point of view.
11) Line 23: Kindly remove “Using panellists”.

Answer: the sentences was adjusted as recommended

12) Lines 23–24: “Kora… rates”: The sentence is badly written in standard English; accordingly, kindly reformulate it.

Answer: the sentences was reformulated

13) Lines 24–25: “Grand… while”: Kindly remove this sentence as reporting non significant findings is inappropriate from the scientific point of view.

Answer: the recommended sentences was removed and reformulated

14) Lines 25–26: “Asgori… color”: The sentence is badly written in standard English; accordingly, kindly reformulate it.

Answer: the sentences was reformulated

15) Line 27: Kindly remove the comma “,” after “that” and remove “On average means of varieties”.

Answer: recommended word and phrases were removed

16) Line 28: Kindly adjust the sentence as follow: “Kora had the highest…” and remove “with lower L*”.

Answer: the sentences was adjusted

17) Lines 28–29: “Kora… quality”: The sentence is badly written in standard English; accordingly, kindly reformulate it.

Answer: the sentences was corrected and reformulated

18) Lines 29–30: “Injera… rate”: Same recommendation as in the previous comment.

Answer: the sentences was adjusted

19) Line 31: The majority of keywords fit well but kindly remlove “and CIEL * a* b values” and adjust the last keyword as follow: “color”.

Answer: the “CIEL * a* b” is removed from the keywords

**1. Introduction**

1) Lines 38–83: The Introduction part is badly written in standard English; accordingly, it should be reformulated in a better language style.

Answer: recommended sentences are reformulated and corrected

2) Lines 38–39: “Tef… Ethiopia”: The sentence is badly written in standard English; accordingly, kindly reformulate it.

Answer: the recommended sentences are corrected and reformulated

3) Lines 38–39: Kindly adjust the in-text references form as follow: “[1–4]”.

Answer: text references are adjusted as recommended by the authors

4) Lines 39–40: “Tef… cultivars”: The sentence is badly written in standard English; accordingly, kindly reformulate it.

Answer: recommended sentence are corrected and formulated

5) Lines 40–43: “Tef… [5,6]”: The sentence is somehow heavy and long; accordingly, kindly reformulate in order to make it more concise, clearer and more aiming.

Answer: the long sentences are shortened and reformulated

6) Lines 44–46: “Tef… [7,8,9]”: The sentence is badly written in standard English; accordingly, kindly reformulate it. Moreover, kindly adjust the in-text references form as follow: “[7–9]”.

Answer: the recommended sentences are reformulated and the text-reference are	adjusted

7) Lines 47–49: “In Ethiopia… [11]”: The sentence is badly written in standard English; accordingly, kindly reformulate it.

Answer: sentences are reformulated and corrected

8) Lines 51–54: “Application… damage”: Same recommendation as in the previous comment.

Answer: the recommended comments are modified and adjusted

9) Lines 57–58: “The application… enhancement”: This statement lacks reliable sources (references); accordingly, kindly provide them.

Answer: the source of the reference are corrected and modified

10) Lines 60–64: “Moreover… nutrients”: Same recommendation as in the previous comment.

Answer: the given comment are adjusted as recommended

11) Line 66: “Nitrogen… practices”: Same recommendation as in the previous two comments.

Answer: the given comment are accepted and adjusted

12) Lines 66–68: “But… [16]”: The sentence is badly written in standard English; accordingly, kindly reformulate it.

Answer: the author tries to adjusted and corrected the given comments

13) Line 69: Kindly adjust as follow: “Gu et al. [13]”.

Answer: the given comment is accepted and adjusted

14) Lines 69–75: “Gu… [13]”: These sentences are badly written in standard English; accordingly, kindly reformulate them.

Answer: the grammatically and the English are reformulated as the given comments

15) Lines 76–79: “However… injera”: Same recommendation as in the previous comment.

Answer: the comments are accepted and reformulated

16) Lines 80–81: “The reason… production”: Kindly remove these sentences.

Answer: the comments are accepted and removed

**2. Materials and methods**

1) Lines 85–158: The Materials and methods part shows numerous needed adjustments in terms of sentences standard English formulation. Moreover, most methods especially related to laboratory analysis lack explanation, therefore they should be added.

Answer: for all experimental methods and laboratory analysis, the methods related to laboratory analysis are accepted and fully explained

2) Study area, lines 86–87: “The study… 2017”: The sentence is badly written in standard English; accordingly, kindly reformulate it.

Answer: the sentences are modified and reformulated

3) Study area, lines 88–91: “The experimental… 832”: Same recommendation as in the previous comment. Moreover, kindly adjust as follow: “832 mm”.

Answer: the comments are accepted and adjusted as recommended

4) Soil sample characterization, lines 93–95: Kindly explain briefly the adopted methods.

Answer: the comments are accepted and the methods are adopted

5) Injera preparations, line 115: Kindly adjust as follow: “were mixed”.

Answer: comment is accepted and modified

6) Injera preparations, lines 115–117: “Therefore… minutes”: The sentence is long and cumbersome; accordingly, kindly reformulate in order to make it more concise, clearer and more aiming.

Answer: the comments are accepted and reformulated

7) Injera preparations, line 118: Kindly replace “for” by “at” and adjust as follow: “were added” and “28–30°C”.

Answer: the comments are accepted and modified using the recommended terminologies

8) Injera preparations, line 119: Kindly adjust the sentence as follow: “for 2–3 hours at 28–30°C and left until…”

Answer: the comments are accepted and adjusted accordingly

9) Injera preparations, lines 119–120: Kindly remove “After… formed” and adjust the sentence as follow: “After that, around 500 ml of the batter were poured…”

Answer: comments are accepted and reformulated

10) Injera preparations, lines 122–123: “Then… obtained”: The sentence is badly written in standard English; accordingly, kindly reformulate it.

Answer: the comment is accepted and reformulated accordingly

11) Image analysis, line 128: Kindly adjust as follow: “45°C”.

Answer: it is accepted and adjusted

12) Image analysis, lines 128–129: “The room… injera”: The sentence is badly written in standard English; accordingly, kindly reformulate it.

Answer: the sentences is reformulated

13) Image analysis, lines 129–130: “A camera… used”: Kindly remove this sentence.

Answer: the comment is accepted and adjusted

14) Image analysis, line 132: Kindly replace “with no” by “without”.

Answer: it is accepted and replaced

15) Image analysis, line 134: Kindly adjust as follow: “were measured”.

Answer: it is adjusted

16) Image analysis, line 135: Kindly adjust as follow: “using the same software”.

Answer: it is adjusted

17) Image analysis, lines 136–137: “Two… samples”: The sentence is badly written in standard English; accordingly, kindly reformulate it.

Answer: the sentence is reformulated

18) Laboratory analysis, line 143: Kindly explain briefly the method of determination of each element.

Answer: comments are accepted and each method are explained accordingly

19) Laboratory analysis, lines 149–152: Same recommendation as in the previous comment.

Answer: comments are accepted and adjusted

20) Statistical analysis, line 156: Kindly remove “And”.

Answer: comment is adjusted

21) Statistical analysis, line 157: Kindly replace “by” by “using”.

Answer: It is adjusted

22) Statistical analysis, line 157: Kindly replace “using” by “following”.

Answer: it is adjusted

23) Statistical analysis, lines 156–158: “The relationship… 0.05”: The sentence is long and cumbersome; accordingly, kindly reformulate it.

Answer: the sentence is reformulated

**3. Results and discussion**

1) Lines 160–525: The Results and discussion part needs major adjustments mainly related to the scientific analysis of obtained findings. However, the statistical approach adopted is correctly performed. On the other hand, I suggest a major revision or re-writing this whole part as it shows uncountable linguistic mistakes and the need for a wide range of sentences reformulation.

Answer: the result and analysis parts are revised as much as possible and adjusted including its reformulations

2) Line 159: Kindly adjust this part’s title as follow: “Results and discussion”.

Answer: it accepted

3) Soil physicochemical properties of the experimental site, lines 161–162: Kindly remove the “( )”.

Answer: the comment is accepted and modified accordingly

4) Soil physicochemical properties of the experimental site, lines 162–163: “Soil… method”: Kindly move this sentence to the Materials and methods part.

Answer: for this comment is not move to material and methods, because it tells us the discussion part of the experimental soil sample of nitrogen content, organic carbon and organic matter as comparing with the dry land soil sample, being the experiment site is found near to the refit valley of the country, Ethiopia. Therefore, the author reformulated and the placed this discussion part as the pervious.

5) Soil physicochemical properties of the experimental site, lines 161–172: I don’t see the need to discuss this table!! Also, it should be moved to the Materials and methods part as it has no place in the current part.

Answer: Being the author wants to discuss the physicochemical characterization of the soil sample, and it was reformulated and clearly stated the implication of the result. Therefore, I think that it is proper place for the discussion of the soil sample.

6) Nutritional Compositions of Tef Grain as Influenced by Nitrogen Fertilizer Rates and Varieties, Moisture content, lines 185–186: “The result… (Table 2)”: Kindly remove this sentence as analyzing nonsignificant findings are inappropriate from the scientific point of view.

Answer: comments are accepted and reformulated after removing the nonsignificant findings

7) Nutritional Compositions of Tef Grain as Influenced by Nitrogen Fertilizer Rates and Varieties, Moisture content, lines 189–191: “And… rates”: Same recommendation as in the previous comment.

Answer: comments are accepted and modified

8) Nutritional Compositions of Tef Grain as Influenced by Nitrogen Fertilizer Rates and Varieties, Moisture content, line 192: Kindly mention that it was an increase and mention the percentage of increase.

Answer: comments are adjusted

9) Nutritional Compositions of Tef Grain as Influenced by Nitrogen Fertilizer Rates and Varieties, Ash content of Tef grain, line 193: Kindly adjust the title of this paragraph as follow: “Ash content of Tef grain”.

Answer: comment is accepted and adjusted

10) Nutritional Compositions of Tef Grain as Influenced by Nitrogen Fertilizer Rates and Varieties, Ash content of Tef grain, line 194: “Ash… minerals”: Kindly remove this sentence.

Answer: comment is accepted and sentences is adjusted

11) Nutritional Compositions of Tef Grain as Influenced by Nitrogen Fertilizer Rates and Varieties, Ash content of Tef grain, lines 194–196: “Both… [35]”: Kindly remove this sentence as analyzing nonsignificant findings is inappropriate from the scientific point of view.

Answer: comment is accepted and adjusted

12) Nutritional Compositions of Tef Grain as Influenced by Nitrogen Fertilizer Rates and Varieties, Ash content of Tef grain, line 196: Kindly remove “On the other hand” and adjust the sentence as follow: “Bultosa [35] obtained…”

Answer: comments are accepted and adjusted and reformulated

13) Nutritional Compositions of Tef Grain as Influenced by Nitrogen Fertilizer Rates and Varieties, Ash content of Tef grain, lines 199–200: “And… (Table 2)”: Kindly remove this sentence as analyzing nonsignificant findings is inappropriate from the scientific point of view.

Answer: comment is accepted and reformulated

14) Nutritional Compositions of Tef Grain as Influenced by Nitrogen Fertilizer Rates and Varieties, Protein content of grain, line 201: Kindly adjust this paragraph’s title as follow: “Protein content of grain”.

Answer: comment is accepted and adjusted as “protein content of tef grain”

15) Nutritional Compositions of Tef Grain as Influenced by Nitrogen Fertilizer Rates and Varieties, Protein content of grain, lines 202–203: “The result… (Table 1)”: The sentence is badly written in standard English; accordingly, kindly reformulate it.

Answer: comment is accepted and reformulated accordingly

16) Nutritional Compositions of Tef Grain as Influenced by Nitrogen Fertilizer Rates and Varieties, Protein content of grain, lines 204–205: Kindly remove “was” and adjust the sentence as follow: “ranged between 9.51 and 11.14% being comparable to the findings of Bultosa [35] (8.7–11%)”.

Answer: comments are accepted accordingly

17) Nutritional Compositions of Tef Grain as Influenced by Nitrogen Fertilizer Rates and Varieties, Protein content of grain, line 205: Kindly adjust as follow: “is expected”.

Answer: comment is adjusted accordingly

18) Nutritional Compositions of Tef Grain as Influenced by Nitrogen Fertilizer Rates and Varieties, Protein content of grain, lines 205–206: “The increase… proteins”: This statement lacks reliable sources (references); accordingly, kindly provide them.

Answer: the sentence is reformulated and adjusted

19) Nutritional Compositions of Tef Grain as Influenced by Nitrogen Fertilizer Rates and Varieties, Protein content of grain, lines 206–207: “When we… (Table)”: Kindly remove this sentence.

Answer: comment is accepted and adjusted

20) Nutritional Compositions of Tef Grain as Influenced by Nitrogen Fertilizer Rates and Varieties, Protein content of grain, line 207: Kindly adjust as follow: “This indicates… may have significant…”

Answer: comment is accepted and adjusted

21) Nutritional Compositions of Tef Grain as Influenced by Nitrogen Fertilizer Rates and Varieties, Crude fat content, lines 217–218: “the ranged… 3.0%”: The sentence is badly written in standard English; accordingly, kindly reformulate it.

Answer: the sentence is adjusted and reformulated

22) Nutritional Compositions of Tef Grain as Influenced by Nitrogen Fertilizer Rates and Varieties, Crude fat content, line 218: Kindly adjust as follow: “accumulation’.

Answer: comment is accepted and adjusted

23) Nutritional Compositions of Tef Grain as Influenced by Nitrogen Fertilizer Rates and Varieties, Crude fat content, lines 218–219: Kindly remove “and have not shown significant differences”.

Answer: comment is accepted and adjusted

24) Nutritional Compositions of Tef Grain as Influenced by Nitrogen Fertilizer Rates and Varieties, Crude fat content, lines 218–221: “The fat… [36]: The sentence is badly written in standard English; accordingly, kindly reformulate it.

Answer: the sentences are adjusted and reformulated

25) Nutritional Compositions of Tef Grain as Influenced by Nitrogen Fertilizer Rates and Varieties, Crude fat content, lines 221–223: “And… varieties”: Kindly remove this sentence.

Answer: comment is accepted and adjusted after removing

26) Nutritional Compositions of Tef Grain as Influenced by Nitrogen Fertilizer Rates and Varieties, Crude fiber, lines 225–226: “The crude… varieties”: Kindly remove this sentence as analyzing nonsignificant findings is inappropriate from the scientific point of view.

Answer: comment is accepted and reformulated

27) Nutritional Compositions of Tef Grain as Influenced by Nitrogen Fertilizer Rates and Varieties, Crude fiber, line 226: Kindly remove “And”.

Answer: comment is accepted and removed

28) Nutritional Compositions of Tef Grain as Influenced by Nitrogen Fertilizer Rates and Varieties, Crude fiber, line 227: Kindly adjust as follow: “with the report of Baye [37]”.

Answer: the sentence is adjusted accordingly

29) Nutritional Compositions of Tef Grain as Influenced by Nitrogen Fertilizer Rates and Varieties, Crude fiber, lines 227–229: “Both… varieties”: Kindly remove this sentence as analyzing nonsignificant findings is inappropriate from the scientific point of view.

Answer: comment is accepted and adjusted after removing

30) Nutritional Compositions of Tef Grain as Influenced by Nitrogen Fertilizer Rates and Varieties, Total carbohydrate and energy content, lines 231–232: “but… rates”: Same recommendation as in the previous comment.

Answer: comment is accepted and adjusted after removing

31) Nutritional Compositions of Tef Grain as Influenced by Nitrogen Fertilizer Rates and Varieties, Total carbohydrate and energy content, line 233: Kindly adjust as follow: “had” and remove “is”.

Answer: comment is accepted and adjusted after removing

32) Nutritional Compositions of Tef Grain as Influenced by Nitrogen Fertilizer Rates and Varieties, Total carbohydrate and energy content, line 234: Kindly adjust as follow: “increased” and remove “was”.

Answer: comment is accepted and adjusted after removing

33) Nutritional Compositions of Tef Grain as Influenced by Nitrogen Fertilizer Rates and Varieties, Total carbohydrate and energy content, line 235: Kindly adjust as follow: “content”.

Answer: comment is accepted and adjusted after removing

34) Nutritional Compositions of Tef Grain as Influenced by Nitrogen Fertilizer Rates and Varieties, Total carbohydrate and energy content, lines 235–237: “But… grains”: Kindly remove this sentence as analyzing nonsignificant findings is inappropriate from the scientific point of view.

Answer: comment is accepted and adjusted after formulated

35) Effects of Nitrogen Fertilizer Rates and Varieties on the Anti-nutritional Factor of Tef Phytic acid content, lines 240–241: “The interaction… grain”: The sentence is badly written in standard English; accordingly, kindly reformulate it.

Answer: comment is accepted and reformulated

36) Effects of Nitrogen Fertilizer Rates and Varieties on the Anti-nutritional Factor of Tef Phytic acid content, line 243: Kindly adjust the sentence as follow: “According to Baye [37]…”

Answer: comment is accepted and adjusted

37) Effects of Nitrogen Fertilizer Rates and Varieties on the Anti-nutritional Factor of Tef Phytic acid content, lines 244–245: Kindly adjust as follow: “ranged between 682 and 1374… being higher than the present findings…”

Answer: comment is accepted and adjusted

38) Effects of Nitrogen Fertilizer Rates and Varieties on the Anti-nutritional Factor of Tef Phytic acid content, lines 243–246: “According… contents”: The sentence is long and cumbersome; accordingly, kindly reformulate in order to make it more concise, clearer and more aiming.

Answer: comment is accepted and reformulated

39) Effects of Nitrogen Fertilizer Rates and Varieties on the Anti-nutritional Factor of Tef Phytic acid content, lines 246–248: “On the other… grains”: Kindly remove this sentence as analyzing nonsignificant findings is inappropriate from the scientific point of view.

Answer: comment is accepted and adjusted after removing

40) Effects of Nitrogen Fertilizer Rates and Varieties on the Anti-nutritional Factor of Tef Phytic acid content, Tannin content of Tef grain, line 260: Kindly adjust this paragraph’s title as follow: “Tannin content of Tef grain”.

Answer: comment is accepted and adjusted

41) Effects of Nitrogen Fertilizer Rates and Varieties on the Anti-nutritional Factor of Tef Phytic acid content, Tannin content of Tef grain, line 261: Kindly remove “only”.

Answer: comment is adjusted after removing

42) Effects of Nitrogen Fertilizer Rates and Varieties on the Anti-nutritional Factor of Tef Phytic acid content, Tannin content of Tef grain, line 262: Higher compared to what? Kindly mention.

Answer: comment is adjusted as suitable for phrases

43) Effects of Nitrogen Fertilizer Rates and Varieties on the Anti-nutritional Factor of Tef Phytic acid content, Tannin content of Tef grain, line 264: Kindly adjust as follow: “to a report of Baye [37]”.

Answer: comment is accepted and adjusted

44) Effects of Nitrogen Fertilizer Rates and Varieties on the Anti-nutritional Factor of Tef Phytic acid content, Tannin content of Tef grain, lines 266–267: “Tannin… (Table 3)”: The sentence is badly written in standard English; accordingly, kindly reformulate it.

Answer: the sentence is adjusted and reformulated

45) Effects of Nitrogen Fertilizer Rates on Mineral Contents of Tef Grain Iron (Fe), line 272: Kindly replace “to” by “and”.

Answer: comment is adjusted

46) Effects of Nitrogen Fertilizer Rates on Mineral Contents of Tef Grain Iron (Fe), line 273: Kindly remove “was found” and adjust as follow “with those of Baye [37]”.

Answer: comment is accepted and adjusted after removing

47) Effects of Nitrogen Fertilizer Rates on Mineral Contents of Tef Grain Iron (Fe), lines 273–274: Kindly adjust as follow: “where the ranges of Fe…”

Answer: comment is accepted

48) Effects of Nitrogen Fertilizer Rates on Mineral Contents of Tef Grain Iron (Fe), line 274: Kindly add the unit.

Answer: comment is corrected

49) Effects of Nitrogen Fertilizer Rates on Mineral Contents of Tef Grain Iron (Fe), lines 273–276: “The present… varieties”: The sentence is long and badly written in standard English; accordingly, kindly reformulate in order to make it more concise, clearer and more aiming.

Answer: the sentence is adjusted and reformulated

50) Effects of Nitrogen Fertilizer Rates on Mineral Contents of Tef Grain Iron (Fe), lines 276–277: “These… [39]”: The sentence is badly written in standard English; accordingly, kindly reformulate it.

Answer: the sentence is adjusted and reformulated

51) Effects of Nitrogen Fertilizer Rates on Mineral Contents of Tef Grain Iron (Fe), line 277: Kindly remove “to this”.

Answer: it is accepted and adjusted after removing

52) Effects of Nitrogen Fertilizer Rates on Mineral Contents of Tef Grain Iron (Fe), lines 277–278: “In addition… contents”: This statement lack reliable sources (references); accordingly, kindly provide them.

Answer: the text reference is provided and adjusted accordingly

53) Effects of Nitrogen Fertilizer Rates on Mineral Contents of Tef Grain Iron (Fe), Calcium (Ca), line 293: Kindly replace “have” by “had”.

Answer: comment is adjusted after removing

54) Effects of Nitrogen Fertilizer Rates on Mineral Contents of Tef Grain Iron (Fe), Calcium (Ca), line 295: Kindly adjust as follow: “contents”.

Answer: comment is adjusted

55) Effects of Nitrogen Fertilizer Rates on Mineral Contents of Tef Grain Iron (Fe), Calcium (Ca), lines 297–299: “The ranges… [37]”: The sentence is badly written in standard English; accordingly, kindly reformulate it.

Answer: the sentence is adjusted and reformulated

56) Effects of Nitrogen Fertilizer Rates on Mineral Contents of Tef Grain Iron (Fe), Calcium (Ca), lines 293–299: The scientific analysis of this section needs to be adjusted; accordingly, kindly reformulate it in a proper manner.

Answer: the sentence is adjusted and reformulated

57) Effects of Nitrogen Fertilizer Rates on Mineral Contents of Tef Grain Iron (Fe), Calcium (Ca), line 300: Kindly replace “from” by “with”.

Answer: it is replaced and adjusted accordingly

58) Effects of Nitrogen Fertilizer Rates on Mineral Contents of Tef Grain Iron (Fe), Calcium (Ca), lines 299–301: “Regardless… Asgori”: The sentence is badly written in standard English; accordingly, kindly reformulate it.

Answer: sentences are adjusted and reformulated

59) Effects of Nitrogen Fertilizer Rates on Mineral Contents of Tef Grain Iron (Fe), Calcium (Ca), line 302: Kindly replace “that” by “where” and adjust as follow: “had higher Ca compared to…”. Also, kindly mention of increase or decrease.

Answer: comment is accepted and adjusted

60) Effects of Nitrogen Fertilizer Rates on Mineral Contents of Tef Grain Iron (Fe), Calcium (Ca), lines 303–304: “From… (Table 4)”: Kindly remove this sentence as analyzing nonsignificant findings is inappropriate from the scientific point of view.

Answer: it is accepted and adjusted after removing

61) Effects of Nitrogen Fertilizer Rates on Mineral Contents of Tef Grain Iron (Fe), Phosphorus content of Tef grain, lines 307–310: “As reported… (Table 4)”: The sentence is long, cumbersome and badly written in standard English; accordingly, kindly reformulate in order to make it more concise, clearer and more aiming.

Answer: sentence is adjusted and reformulated

62) Effects of Nitrogen Fertilizer Rates on Mineral Contents of Tef Grain Iron (Fe), Phosphorus content of Tef grain, line 311: Kindly adjust as follow: “fertilizers”.

Answer: it is accepted and adjusted

63) Effects of Nitrogen Fertilizer Rates on Mineral Contents of Tef Grain Iron (Fe), Phosphorus content of Tef grain, line 313: Kindly remove “slightly”.

Answer: it is adjusted and removed

64) Effects of Nitrogen Fertilizer Rates and Tef Varieties on Sensory Quality of Injera, line 314: Kindly adjust the paragraph’s title as follow: “Effects of Nitrogen Fertilizer Rates and Tef Varieties on Sensory Quality of Injera”.

Answer: it is accepted and adjusted

65) Effects of Nitrogen Fertilizer Rates and Tef Varieties on Sensory Quality of Injera, line 316: Kindly remove “( )”.

Answer: it removed and adjusted

66) Effects of Nitrogen Fertilizer Rates and Tef Varieties on Sensory Quality of Injera, line 317: Kindly adjust as follow: “was baked”.

Answer: it is replaced and adjusted

67) Effects of Nitrogen Fertilizer Rates and Tef Varieties on Sensory Quality of Injera, Sensory evaluation of Injera, line 319: Kindly adjust this paragraph’s title as follow: “Sensory evaluation of Injera”.

Answer: it is accepted and adjusted accordingly

68) Effects of Nitrogen Fertilizer Rates and Tef Varieties on Sensory Quality of Injera, Sensory evaluation of Injera, Color, line 327: Kindly adjust as follow: “maximal”.

Answer: it is adjusted

69) Effects of Nitrogen Fertilizer Rates and Tef Varieties on Sensory Quality of Injera, Sensory evaluation of Injera, Color, lines 326–329: “Average… use”: The sentence is long and cumbersome; accordingly, kindly reformulate in order to make it more concise, clearer and more aiming.

Answer: sentence is adjusted and reformulated

70) Effects of Nitrogen Fertilizer Rates and Tef Varieties on Sensory Quality of Injera, Sensory evaluation of Injera, Color, lines 331–334: “Injera… [42]”: These sentences are badly written in standard English; accordingly, kindly reformulate them.

Answer: the sentence is adjusted and reformulated

71) Effects of Nitrogen Fertilizer Rates and Tef Varieties on Sensory Quality of Injera, Sensory evaluation of Injera, Flavor, lines 337–338: Kindly remove “but not have increasing or decreasing trends” and adjust as follow: “with Gu et al. [13] who reported…”

Answer: it is accepted and adjusted after removing

72) Effects of Nitrogen Fertilizer Rates and Tef Varieties on Sensory Quality of Injera, Sensory evaluation of Injera, Flavor, lines 340–342: “This may… evaluations”: The sentence is badly written in standard English; accordingly, kindly reformulate it.

Answer: sentence is adjusted and reformulated

73) Effects of Nitrogen Fertilizer Rates and Tef Varieties on Sensory Quality of Injera, Sensory evaluation of Injera, Flavor, lines 342–343: “Average… injera”: Kindly remove this sentence as analyzing non significant findings is inappropriate from the scientific point of view.

Answer: it is removed and adjusted

74) Effects of Nitrogen Fertilizer Rates and Tef Varieties on Sensory Quality of Injera, Sensory evaluation of Injera, Texture, lines 348–349: “Texture… softness”: The sentence is badly written in standard English; accordingly, kindly reformulate it.

Answer: sentence is adjusted and reformulated

75) Effects of Nitrogen Fertilizer Rates and Tef Varieties on Sensory Quality of Injera, Sensory evaluation of Injera, Texture, lines 349–350: “Injera… [42]”: Same recommendation as in the previous comment.

Answer: sentence is adjusted and reformulated

76) Effects of Nitrogen Fertilizer Rates and Tef Varieties on Sensory Quality of Injera, Sensory evaluation of Injera, Texture, line 356: Kindly adjust as follow: “with the present study”.

Answer: it is adjusted accordingly

77) Effects of Nitrogen Fertilizer Rates and Tef Varieties on Sensory Quality of Injera, Sensory evaluation of Injera, Taste, lines 358–360: “From… dislike”: The sentence is badly written in standard English; accordingly, kindly reformulate it.

Answer: sentence is adjusted and reformulated

78) Effects of Nitrogen Fertilizer Rates and Tef Varieties on Sensory Quality of Injera, Sensory evaluation of Injera, Taste, lines 360–363: “The sensory… excellent”: The sentence is long, cumbersome and badly written in standard English; accordingly, kindly reformulate in order to make it more concise, clearer and more aiming.

Answer: the sentence is modified and reformulated

79) Effects of Nitrogen Fertilizer Rates and Tef Varieties on Sensory Quality of Injera, Sensory evaluation of Injera, Taste, lines 363–365: “And… injera”: Same recommendation as in the previous comment.

Answer: the sentence is modified and reformulated

80) Effects of Nitrogen Fertilizer Rates and Tef Varieties on Sensory Quality of Injera, Sensory evaluation of Injera, Taste, lines 366–369: “Mean… (Table 5)”: Same recommendation as in the previous two comments.

Answer: the sentence is modified and reformulated

81) Effects of Nitrogen Fertilizer Rates and Tef Varieties on Sensory Quality of Injera, Sensory evaluation of Injera, Folding capacity (FC), lines 372–374: “However… slightly”: Same recommendation as in the previous comments.

Answer: sentence is modified and reformulated

82) Effects of Nitrogen Fertilizer Rates and Tef Varieties on Sensory Quality of Injera, Sensory evaluation of Injera, Eye size (ES) and eye distribution (ED) of injera, lines 382–385: “From… slightly”: Same recommendation as in the previous comments.

Answer: sentence is adjusted and modified with reformulations

83) Effects of Nitrogen Fertilizer Rates and Tef Varieties on Sensory Quality of Injera, Sensory evaluation of Injera, Eye size (ES) and eye distribution (ED) of injera, lines 386–388: “This… injera”: Same recommendation as in the previous comments.

Answer: the sentence is adjusted and modified with reformulations

84) Effects of Nitrogen Fertilizer Rates and Tef Varieties on Sensory Quality of Injera, Sensory evaluation of Injera, Eye size (ES) and eye distribution (ED) of injera, line 397: Kindly adjust as follow: “affect”.

Answer: it is accepted and adjusted

85) Effects of Nitrogen Fertilizer Rates and Tef Varieties on Sensory Quality of Injera, Sensory evaluation of Injera, Top and bottom surfaces (TBS) of injera, lines 401–402: Kindly adjust as follow: “were rated”.

Answer: it is accepted and adjusted

86) Effects of Nitrogen Fertilizer Rates and Tef Varieties on Sensory Quality of Injera, Sensory evaluation of Injera, Top and bottom surfaces (TBS) of injera, lines 402–403: Kindly adjust the in-text reference form according to the journal’s guidelines.

Answer: it accepted and adjusted accordingly

87) Effects of Nitrogen Fertilizer Rates and Tef Varieties on Sensory Quality of Injera, Sensory evaluation of Injera, Top and bottom surfaces (TBS) of injera, lines 402–405: “The study… varieties”: The sentence is badly written in standard English; accordingly, kindly reformulate it.

Answer: sentence is adjusted and modified with some reformulations

88) Effects of Nitrogen Fertilizer Rates and Tef Varieties on Sensory Quality of Injera, Sensory evaluation of Injera, Overall acceptability of injera quality, lines 413–414: “Overall… product”: Same recommendation as in the previous comment.

Answer: sentence is adjusted and modified with some reformulations

89) Effects of Nitrogen Fertilizer Rates and Tef Varieties on Sensory Quality of Injera, Sensory evaluation of Injera, Overall acceptability of injera quality, line 419: Kindly remove “And”.

Answer: it is accepted and adjusted

90) Effects of Nitrogen Fertilizer Rates and Tef Varieties on Sensory Quality of Injera, Sensory evaluation of Injera, Overall acceptability of injera quality, line 420: It is a significant difference!! You cannot say “minor” !!

Answer: it is accepted and adjusted

91) Effects of Nitrogen Fertilizer Rates and Tef Varieties on Sensory Quality of Injera, Sensory evaluation of Injera, Overall acceptability of injera quality, lines 419–424: “Injera… present”: These sentences are badly written in standard English; accordingly, kindly reformulate them.

Answer: sentence is reformulated and adjusted

92) Effects of Nitrogen Fertilizer Rates and Tef Varieties on Sensory Quality of Injera, Sensory evaluation of Injera, Total number of holes and filtered eyes of injera, line 430: Kindly remove “( )”.

Answer: it is accepted and adjusted after removed

93) Effects of Nitrogen Fertilizer Rates and Tef Varieties on Sensory Quality of Injera, Sensory evaluation of Injera, Total number of holes and filtered eyes of injera, Number of holes of injera, lines 432–434: “A number… (Table 6)”: The sentence is badly written in standard English; accordingly, kindly reformulate it.

Answer: sentence is modified and reformulated

94) Effects of Nitrogen Fertilizer Rates and Tef Varieties on Sensory Quality of Injera, Sensory evaluation of Injera, Total number of holes and filtered eyes of injera, Number of holes of injera, line 434: Kindly adjust as follow: “may be due”.

Answer: it is accepted and adjusted

95) Effects of Nitrogen Fertilizer Rates and Tef Varieties on Sensory Quality of Injera, Sensory evaluation of Injera, Total number of holes and filtered eyes of injera, Number of holes of injera, lines 435–438: “Thus… software”: The sentence is long and cumbersome; accordingly, kindly reformulate in order to make it more concise, clearer and more aiming.

Answer: sentence is adjusted ad modified with reformulations

96) Effects of Nitrogen Fertilizer Rates and Tef Varieties on Sensory Quality of Injera, Sensory evaluation of Injera, Total number of holes and filtered eyes of injera, Filtered eyes of injera, lines 449–457: “For… respectively”: These sentences are badly written in standard English; accordingly, kindly reformulate them.

Answer: sentence is reformulated

97) Effects of Nitrogen Fertilizer Rates and Tef Varieties on Sensory Quality of Injera, Sensory evaluation of Injera, Total number of holes and filtered eyes of injera, Filtered eyes of injera, line 462: Kindly adjust as follow: “was obtained”.

Answer: it it accepted and followed accordingly

98) Effects of Nitrogen Fertilizer Rates and Tef Varieties on Sensory Quality of Injera, Sensory evaluation of Injera, Total number of holes and filtered eyes of injera, Filtered eyes of injera, lines 463–467: “Based… investigations”: The sentence is long and badly written in standard English; accordingly, kindly reformulate in order to make it more concise, clearer and more aiming.

Answer: sentence is reformulated with some modifications

99) Effects of Nitrogen Fertilizer Rates and Tef Varieties on Sensory Quality of Injera, Colors (CIE L* a* b*) values of injera, Lightness (L) color of injera, lines 471–473: “The more… injera”: The sentence is badly written in standard English; accordingly, kindly reformulate it.

Answer: sentence is reformulated

100) Effects of Nitrogen Fertilizer Rates and Tef Varieties on Sensory Quality of Injera, Colors (CIE L* a* b*) values of injera, Lightness (L) color of injera, lines 474–475: “All… [45]”: Same recommendation as in the previous comment.

Answer: sentence is reformulated

101) Effects of Nitrogen Fertilizer Rates and Tef Varieties on Sensory Quality of Injera, Colors (CIE L* a* b*) values of injera, Lightness (L) color of injera, lines 476–483: “Interactions… (Table 6)”: Same recommendation as in the previous two comments.

Answer: sentence is modified and reformulated

102) Effects of Nitrogen Fertilizer Rates and Tef Varieties on Sensory Quality of Injera, Colors (CIE L* a* b*) values of injera, Redness (a*) color of injera, lines 485–489: “The analysis… values”: Same recommendation as in the previous comments.

Answer: sentence is modified and reformulated

103) Effects of Nitrogen Fertilizer Rates and Tef Varieties on Sensory Quality of Injera, Colors (CIE L* a* b*) values of injera, Redness (a*) color of injera, line 492: Kindly replace “from” by “for”.

Answer: it is accepted and replaced

104) Effects of Nitrogen Fertilizer Rates and Tef Varieties on Sensory Quality of Injera, Colors (CIE L* a* b*) values of injera, Redness (a*) color of injera, lines 494–497: “This… variety”: These sentences are badly written in standard English; accordingly, kindly reformulate them.

Answer: sentence is modified and reformulated

105) Effects of Nitrogen Fertilizer Rates and Tef Varieties on Sensory Quality of Injera, Colors (CIE L* a* b*) values of injera, Yellowness (b*) color of injera, lines 514–518: “This… respectively”: Same recommendation as in the previous comment.

Answer: sentence is modified and reformulated accordingly

106) Effects of Nitrogen Fertilizer Rates and Tef Varieties on Sensory Quality of Injera, Colors (CIE L* a* b*) values of injera, Yellowness (b*) color of injera, lines 520–521: “This… varieties”: Same recommendation as in the previous two comments.

Answer: sentence is modified with some reformulations

**4. Conclusions**

1) Lines 527–544: Regarding the Conclusions part, it summarizes the study’s findings and suggests further research. Only minor adjustments are required related to sentences reformulation.

Answer: it is accepted and re-edited accordingly

2) Lines 527–529: “The study… decreased”: The sentence is heavy; accordingly, kindly reformulate in order to make it clearer and more aiming.

Answer: sentence is modified and reformulated

3) Lines 530–531: “The subjective… effects”: Kindly remove this sentence as in the Conclusions part, we only point out the significant findings.

Answer: it is accepted and adjusted accordingly

4) Lines 532–533: Kindly remove “but slightly decreased with N rates”.

Answer: it is accepted and adjusted

5) Lines 537–540: “Grand… injera”: Kindly remove these sentences as in the Conclusions part, we only point out the significant findings.

Answer: it is accepted and adjusted

6) Lines 540–541: “In general… differences”: The sentence is badly written in standard English; accordingly, kindly reformulate it.

Answer; sentence is modified and reformulated

7) Lines 541–544: “Based… dough”: The sentence is long, cumbersome and heavy; accordingly, kindly reformulate in order to make it more concise, clearer and more aiming.

Answer: sentence is modified and reformulated

Reviewers' comments:

Reviewer's Responses to Questions

**Comments to the Author**
1. Is the manuscript technically sound, and do the data support the conclusions?

The manuscript must describe a technically sound piece of scientific research with data that supports the conclusions. Experiments must have been conducted rigorously, with appropriate controls, replication, and sample sizes. The conclusions must be drawn appropriately based on the data presented.

Reviewer #1: Yes

Reviewer #2: Yes

Reviewer #3: Partly

2. Has the statistical analysis been performed appropriately and rigorously?

Reviewer #1: Yes

Reviewer #2: Yes

Reviewer #3: No

3. Have the authors made all data underlying the findings in their manuscript fully available?

The [PLOS Data policy](http://www.plosone.org/static/policies.action#sharing) requires authors to make all data underlying the findings described in their manuscript fully available without restriction, with rare exception (please refer to the Data Availability Statement in the manuscript PDF file). The data should be provided as part of the manuscript or its supporting information, or deposited to a public repository. For example, in addition to summary statistics, the data points behind means, medians and variance measures should be available. If there are restrictions on publicly sharing data—e.g. participant privacy or use of data from a third party—those must be specified.

Reviewer #1: Yes

Reviewer #2: Yes

Reviewer #3: Yes

4. Is the manuscript presented in an intelligible fashion and written in standard English?

PLOS ONE does not copyedit accepted manuscripts, so the language in submitted articles must be clear, correct, and unambiguous. Any typographical or grammatical errors should be corrected at revision, so please note any specific errors here.

Reviewer #1: No

Reviewer #2: No

Reviewer #3: No

5. Review Comments to the Author

Please use the space provided to explain your answers to the questions above. You may also include additional comments for the author, including concerns about dual publication, research ethics, or publication ethics. (Please upload your review as an attachment if it exceeds 20,000 characters)

Reviewer #1: The survey reports an interesting topic that points out the influence of nitrogen fertilizer rate and variety on Tef [Eragrostis tef (Zucc.) Trotter] nutritional composition and sensory quality of a staple bread (injera). Despite that the study didn’t detected significant improvements with increasing N rates; however, scientifically, reporting these findings is very important and crucial. The manuscript presents a huge gap in its standard English; accordingly, I suggest passing it to a native English speaker for an extended editing. The Financial Disclosure and Ethics Statement should be adjusted according to the journal’s guidelines.
The Abstract part should be re-written due to an extensive English language issue. The majority of the keywords fit. The Introduction part is badly written in standard English; accordingly, it should be reformulated in a better language style. The Materials and methods part shows numerous needed adjustments in terms of sentences standard English formulation. Moreover, most methods especially related to laboratory analysis lack explanation, therefore they should be added. The Results and discussion part needs major adjustments mainly related to the scientific analysis of obtained findings. However, the statistical approach adopted is correctly performed. On the other hand, I suggest a major revision or re-writing this whole part as it shows uncountable linguistic mistakes and the need for a wide range of sentences reformulation. Regarding the Conclusions part, it summarizes the study’s findings and suggests further research. Only minor adjustments are required related to sentences reformulation.
Based on the above and below detailed explanation, recommendations and suggestions, I think that the manuscript shows a certain merit to be published in “PLoS One” once all suggestions and recommendations are fully taken into consideration and well addressed. Detailed comments, suggestions and recommendations are found within the attached file.

Reviewer #2: Abstract

Line 15, 18: Avoid using acronyms in summary.

**Answer: acronyms in abstract are removed and fully described**

Keywords

Line 31: avoid repeating the same ones in the title, (CIEL*a*b) Space of Color? could you specify something more general in relation to the CIEL*a*b, it is not clear what CIEL?

**Answer: CIEL*a*b stands for color: L= lightness, a= redness and b= yellowness colors of injera. And the comment is accepted and adjusted**

Introduction

Line 38-39: It is not necessary to cite so many references at this point, just keep the most current one.

**Answer: All references are appropriate fit with this and recently published,**

Line 49: productivity of tef is 1.664 t/ha

**Answer: it is accepted and fully described as tons per hectare**

Line 50 to 54: Improve the wording and focus on the importance of nitrogen in cereals.

**Answer: it is accepted and adjusted**

Line 68-69: it is narrated as a discussion, not as an introduction to the work.

-There is a need to improve the connections between paragraphs and to write as an introduction to the topic, unclear and poorly structured.

**Answer: it is accepted and modified, but it was used as indications of the effects of excess N on organoleptic and biological proteins in the previous studies.**

Material and Methods

Line 119: @ typing mistake?

Line 128: 45 (Grades) typing mistake

Line 143: first time in extenso acronyms

**Answer: there were accepted and adjusted accordingly**

Result and discussion

Objective: “this study mainly focused on the Influence of Nitrogen Fertilizer Rate and Variety Tef Nutritional Composition and Sensory Quality of a Staple bread (Injera)”

Line 160 to 172: does not respond to the objectives as described does not correspond to the results.

**Answer: Line 160 to 172 described the soil physiochemical characterization of the experimental site, in which the author wanted to express the soil N profiles of the experimental sample before applying the recommended N fertilizer. However, it pointed out that the effect of N fertilizer on the recommend objectives of the studies as comparing the expense of the N , organic matter and organic carbon of the previous experimental site.**

Line 177 to 181: Table 1, could be included as supplementary information in materials and methods.

**Answer:** **for this comment is not move to material and methods, because it tells us the discussion part of the experimental soil sample of nitrogen content, organic carbon and organic matter as comparing with the dry land soil sample, being the experiment site is found near to the refit valley of the country, Ethiopia. Therefore, the author reformulated and the placed this discussion part as the pervious**.

- Focus on the most relevant results and responding to the objective set. If they were not significant, the variables that were not significant for the treatments used and the varieties evaluated can be listed in a single paragraph.

Conclusion

- Not responding to the objective concretely, it is a summary of the results obtained, not a conclusion based on the results.

**Answer: all the entire question raised out are addressed properly and answered and accepted as commented and taken measurements as modifications accordingly.**

Reviewer #3: The manuscript “Influence of Nitrogen Fertilizer Rate and Variety on Tef [Eragrostis tef (Zucc.) Trotter] Nutritional Composition and Sensory Quality of a Staple bread (Injera)” submitted by Dagnaw et al. in PONE described a large dataset and a good work conducted by team. However, this manuscript contains some flaws. Before reaching further decision the address and revision in manuscript is necessary:

1. Line no 13: imbalanced instead of unbalanced

- **It is accepted and adjusted**

2. Line no 15: the use of nitrogenous fertilizers

- **It is accepted and adjusted**

3. Line 19: ‘Crop attributes’ instead of ‘Recommended parameters’

- **It is accepted and adjusted**

4. Line 38-39: the number of reference need to reduce

- References used as it is.

5. Line 49: Mg ha-1 instead of t ha-1

- **It is used for the unit of production tonnes per hectare (t ha-1)**

6. Line 57: imbalanced instead of unbalanced

- **It is accepted and adjusted**

7. Line 68: no need of italic

- **It is accepted and adjusted**

8. Line 69: full form

- **It is accepted and adjusted**

9. Line 82-83: lowercase

- It is accepted and adjusted

10. Line 94: Olsen's method

- **It is adjusted and corrected**

11. Line 95: soil: water ratio

- **It is corrected and adjusted**

12. Line 100: triple super phosphate; lower case

- **It is accepted and adjusted**

13. Line 100: multiple sign ‘×’ not X

- **It is adjusted**

14. Line 108: superscript

- **It corrected**

15. Line 119: use degree sign;

- **It is corrected**

16. Line 119: iron and other use lowercase

- **It is corrected accordingly**

17. Line 162-63: part of materials and methods

- **This was not moved into materials and method because the author wanted to discuss the soil nitrogen status of the experimental site before planting applying n fertilizer), therefore, it necessary to put in the result and discussion part**.

18. Line 182-83: Tef grain as influenced by nitrogen fertilizer rates and varieties

- It is accepted and corrected

19. Line 209: 9.88a ± 0.033 ; follow for all tables as applicable

- **It is accepted and adjusted accordingly**

20. Table no. 3 and 4 can be converted to Figures as the whole manuscript showing similar type of Tables.

- **However almost all tables are similar in design, but we have tried to convert table 3 and 4 into figures, but it is that much descried as the table provides the required information, thus we put both tables previous.**

21. Include some multivariate analyses for concluding valid recommendation among the nutritional and sensory indicator of Tef.

6. PLOS authors have the option to publish the peer review history of their article (what does this mean?). If published, this will include your full peer review and any attached files.

If you choose “no”, your identity will remain anonymous but your review may still be made public.

- **YES**

Do you want your identity to be public for this peer review? For information about this choice, including consent withdrawal, please see our Privacy Policy.

Reviewer #1: No

Reviewer #2: Yes: Cinthia Carolina Cazal Martinez

Reviewer #3: Yes: DR NIRMALENDU BASAK
