# Supplementary material for: Influence of nitrogen fertilizer rate and variety on tef [Eragrostis tef (Zucc.) Trotter] nutritional composition and sensory quality of a staple bread (Injera)
Source: PLoS One. 2024 Jan 2;19(1):e0295491. doi: 10.1371/journal.pone.0295491 (PMC10760880; doi:10.1371/journal.pone.0295491)
Supplement: Attachment — Submitted filename: Response to reviewers (Hayelom Berhe Dagnaw).docx [file pone.0295491.s005.docx]

**Answers for all comments accordingly**

For all comments given from the reviewers; comments are accepted and adjusted accordingly in the main bodies of the article.

1. **Abstract**
1. Line 14: Kindly adjust as follow: “are considered”.

- **Answer**: the comment is accepted and replaced.

2. Line 24: Kindly adjust as follow: “but they decreased”.

- **Answer**: the comment is accepted and replaced.

3. Lines 24–25: “Asgori… injera”: The sentence is badly written in Standard English; accordingly, kindly reformulate it.

- **Answer**: the sentences is paraphrase and reformulated

4. Line 27: Kindly remove “had lower injera quality”.

- **Answer**: the comment is accepted and adjusted.

2. **Introduction**
1. Line 39: “Tef… Ethiopia”: The sentence is badly written in standard English; accordingly, kindly reformulate it.

- **Answer**: comment is accepted and sentences are reformulated.

2. Lines 39–40: “Tef… wheat”: Same recommendation as in the previous comment.

- **Answer:** comment is accepted and the sentences are reformulated.

3. Lines 41–44: “Tef… [5,6]”: Same recommendation as in the previous two comments.

- **Answer:** the comment is accepted and the sentences are reformulated

4. Lines 44–45: “Tef… [7–9]”: Same recommendation as in the previous comments.

- **Answer:** the comment is accepted and the sentences are reformulated

5. Lines 46–47: “Off… fertility”: Same recommendation as in the previous comment.

- **Answer:** the comment is accepted and the sentences are reformulated

6. Line 48: Kindly adjust as follow: “compared to”.

- **Answer:** the comment is accepted and replaced

7. Lines 50–53: “However… damage”: These sentences are badly written in standard English; accordingly, kindly reformulate them.

- **Answer:** the comment is accepted and the sentences are reformulated

8. Lines 66–67: “Study… proteins”: Same recommendation as in the previous comment.

- **Answer:** the comment is accepted and the sentences are reformulated

9. Line 73: Kindly remove “However”.

- **Answer:** the comment is accepted and it is removed and adjusted.

10. Line 76: Kindly replace “However” by “On the other hand”

- **Answer:** the comment is accepted and replaced

3. **Materials and methods**
1. Soil sample characterization, line 90: Kindly remove “And” from the beginning of the sentence.

- **Answer:** The comment is accepted and adjusted.

2. Soil sample characterization, line 95: Kindly remove “by” before and after “using” and remove “And” from the beginning of the sentence.

- **Answer:** the comment is accepted and adjusted.

3. Soil sample characterization, line 98: Kindly remove “And” from the beginning of the sentence.

- **Answer:** the comment is accepted and the sentences are reformulated

4. Soil sample characterization, line 99: Kindly adjust as follow: “were added” and “allowed to stand”.

- **Answer:** the comment is accepted and the words are reformulated.

5. Soil sample characterization, line 100: Kindly remove “for”.

- **Answer:** the comment is accepted and adjusted

6. Soil sample characterization, line 103: Kindly remove “And” from the beginning of the sentence.

- **Answer:** the comment is accepted and removed and adjusted

7. Soil sample characterization, lines 88–104: Kindly mention the full specification of the used instrumentation.

- **Answer:** the comment is accepted and all the instrument specifications are fully corrected accordingly

8. Experimental design, line 111: Kindly replace “is” by “was”.

- **Answer:** the comment is accepted and replaced as recommended.

9. Grain flour preparation, line 118: Kindly remove “first”.

- **Answer:** the comment is accepted and is removed

10. Injera preparations, line 123: Kindly adjust as follow: “were mixed”.

- **Answer:** the comment is accepted and the word is adjusted,

11. Injera preparations, lines 125–126: Kindly adjust as follow: “℃”.

- **Answer:** the comment is accepted and the unit is adjusted

12. Injera preparations, line 127: Kindly adjust as follow: “were poured”.

- **Answer:** the comment is accepted and adjusted

13. Image analysis, line 135: Kindly adjust as follow: “To obtain”.

- **Answer:** the comment is accepted and adjusted

14. Image analysis, line 136: Kindly adjust as follow: “45°C”.

- **Answer**: the comment is accepted and the unit is adjusted

15. Image analysis, lines 136–137: “Before… walls”: The sentence is badly written in standard English; accordingly, kindly reformulate it.

- **Answer:** the comment is accepted and the sentences are reformulated

16. Image analysis, lines 140–143: “Colors… on it”: Same recommendation as in the previous comment.

- **Answer:** the comment is accepted and the sentences are reformulated

17. Laboratory analysis, proximate analysis, lines 154–155: “Moisture… [25]”: Same recommendation as in the previous two comments.

- **Answer:** the comment is accepted and the sentences are reformulated

18. Laboratory analysis, proximate analysis, lines 155–159: “A crucible… (W3)”: Same recommendation as in the previous comments.

- **Answer:** the comment is accepted and the sentences are reformulated

19. Laboratory analysis, Proximate analysis, lines 161–162: “The ash… 923.03”: Same recommendation as in the previous comments.

- **Answer:** the comment is accepted and the sentences are reformulated

20. Laboratory analysis, proximate analysis, line 164: Kindly adjust as follow: “were weighed”.

- **Answer:** the comment is accepted and adjusted.

21. Laboratory analysis, proximate analysis, lines 164–166: “Finally… charred”: The sentence is badly written in Standard English; accordingly, kindly reformulate it.

- **Answer:** the comment is accepted and the sentences are reformulated

22. Laboratory analysis, proximate analysis, lines 171–175: “Air… 1 hour”: Same recommendation as in the previous comment.

- **Answer:** the comment is accepted and the sentences are reformulated

23. Laboratory analysis, Proximate analysis, lines 181–183: “On the other… solvent”: Same recommendation as in the previous two comments.

- **Answer:** the comment is accepted and the sentences are reformulated

24. Laboratory analysis, proximate analysis, line 185: Kindly adjust as follow: “103℃”.

- **Answer**: the comment is accepted and adjusted

25. Laboratory analysis, Determination of mineral content, lines 194–201: “Iron… prepared”: These sentences are badly written in standard English; accordingly, kindly reformulate them.

- **Answer:** the comment is accepted and the sentences are reformulated

26. Laboratory analysis, Determination of mineral content, lines 202–203: Kindly adjust as follow: “were prepared”.

- **Answer:** the comment is accepted and adjusted

27. Laboratory analysis, Determination of mineral content, line 209: Kindly adjust as follow: “were measured”.

- **Answer:** the comment is accepted and adjusted.

28. Laboratory analysis, Determination of mineral content, lines 211–214: “After… completed”: The sentence is badly written in standard English; accordingly, kindly reformulate it.

- **Answer:** the comment is accepted and the sentences are reformulated

29. Laboratory analysis, Determination of mineral content, line 215: Kindly adjust as follow: “were added”.

- **Answer:** the comment is accepted and adjusted

30. Laboratory analysis, Determination of mineral content, lines 216–223: “The solution… water”: These sentences are badly written in Standard English; accordingly, kindly reformulate them.

- **Answer:** the comment is accepted and the sentences are reformulated

31. Laboratory analysis, Determination of mineral content, lines 223 and 225: Kindly adjust as follow: “were prepared”.

- **Answer:** the comment is accepted and the sentences are reformulated

32. Laboratory analysis, Determination of phytic acid content, line 229: Kindly adjust as follow: “Determination”.

- **Answer:** the comment is accepted and the sentences are reformulated

33. Laboratory analysis, Determination of phytic acid content, lines 230–231: “The amount… [27]”: The sentence is badly written in Standard English; accordingly, kindly reformulate it.

- **Answer:** the comment is accepted and the sentences are reformulated

34. Laboratory analysis, Determination of phytic acid content, line 233: Kindly adjust as follow: “were added”.

- **Answer:** the comment is accepted and added and adjusted

35. Laboratory analysis, Determination of phytic acid content, lines 236–240: “A series… tubes”: These sentences are badly written in Standard English; accordingly, kindly reformulate them.

- **Answer:** the comment is accepted and the sentences are reformulated

36. Laboratory analysis, Determination of phytic acid content, lines 242–243: “Using… (1)”: Same recommendation as in the previous comment.

- **Answer:** the comment is accepted and the sentences are reformulated

37. Laboratory analysis, Determination of phytic acid content, line 244: Kindly adjust the formula presentation following the journal’s guidelines.

- **Answer:** the comment is accepted and the formula is adjusted according to the standard

38. Laboratory analysis, Determination of tannin content, line 245: Kindly adjust as follow: “Determination”.

- **Answer:** the comment is accepted and adjusted accordingly

39. Laboratory analysis, Determination of tannin content, line 249: Kindly adjust as follow: “were added”.

- **Answer:** the comment is accepted and the sentences are reformulated

40. Laboratory analysis, Determination of tannin content, lines 250–251: “The samples… temperature”: The sentence is badly written in Standard English; accordingly, kindly reformulate it.

- **Answer:** the comment is accepted and the sentences are reformulated

41. Laboratory analysis, Determination of tannin content, lines 254–256: “Working… methanol”: Same recommendation as in the previous comment.

- **Answer:** the comment is accepted and the sentences are reformulated accordingly

42. Laboratory analysis, Determination of tannin content, lines 256–259: “Five… 500 nm”: Same recommendation as in the previous two comments.

- **Answer:** the comment is accepted and the sentences are reformulated

43. Laboratory analysis, Determination of tannin content, line 262: Kindly adjust the formula presentation following the journal’s guidelines.

- **Answer**: the comment is accepted and the formula is adjusted according to the guidelines

44. Laboratory analysis, Determination of tannin content, line 263: Kindly adjust as follow: “were determined”.

- **Answer**: the comment is accepted and adjusted and replaced

45. Statistical analysis, lines 267–269: “The data… used”: The sentence is badly written in standard English; accordingly, kindly reformulate it.

- **Answer**: the comment is accepted and the sentences are reformulated

4. **Results and discussion**
1. Soil physicochemical properties of the experimental site, lines 274–280: “The result… minerals”: These sentences are badly written in Standard English; accordingly, kindly reformulate them.

- **Answer:** the comment is accepted and the sentences are reformulated

2. Tef Grain as Influenced by Nitrogen Fertilizer Rates and Varieties, Moisture content, line 293: Kindly remove “G.”.

- **Answer:** the comment is accepted and canceled

3. Tef Grain as Influenced by Nitrogen Fertilizer Rates and Varieties, Moisture content, line 296: Kindly replace “have” by “had” and adjust as follow: “as compared to”.

- **Answer:** the comment is accepted and adjusted

4. Tef Grain as Influenced by Nitrogen Fertilizer Rates and Varieties, Moisture content, line 297: Kindly adjust as follow: “(table 2)”.

- **Answer:** the comment is accepted and adjusted

5. Tef Grain as Influenced by Nitrogen Fertilizer Rates and Varieties, Ash content of tef grain, lines 299–301: “The analysis… variety”: These sentences are badly written in Standard English; accordingly, kindly reformulate them.

- **Answer**: the comment is accepted and the sentences are reformulated

6. Tef Grain as Influenced by Nitrogen Fertilizer Rates and Varieties, Protein content of tef grain, line 305: Kindly adjust as follow: “(Table 1)”.

- **Answer:** the correction are accepted and adjusted

7. Tef Grain as Influenced by Nitrogen Fertilizer Rates and Varieties, Protein content of tef grain, line 307: Kindly adjust as follow: “from that reported” and remove “G.”.

- **Answer:** the comment is accepted and adjusted accordingly

8. Tef Grain as Influenced by Nitrogen Fertilizer Rates and Varieties, Protein content of tef grain, lines 307–309: “Being… expected”: The sentence is badly written in standard English; accordingly, kindly reformulate it.

- **Answer:** the comment is accepted and the sentences are reformulated

9. Tef Grain as Influenced by Nitrogen Fertilizer Rates and Varieties, Protein content of tef grain, line 309: Kindly adjust as follow: “significant”.

- **Answer:** the comment is accepted and replaced accordingly

10. Tef Grain as Influenced by Nitrogen Fertilizer Rates and Varieties, Crude fat content, line 319: Kindly adjust as follow: “(Table 2)”.

- **Answer:** the comment is accepted and adjusted

11. Tef Grain as Influenced by Nitrogen Fertilizer Rates and Varieties, Crude fat content, line 320: Kindly remove “G.”.

- **Answer:** the comment is accepted and canceled accordingly

12. Tef Grain as Influenced by Nitrogen Fertilizer Rates and Varieties, Crude fat content, lines 320–323: “The fat… [36]”: These sentences are badly written in standard English; accordingly, kindly reformulate them.

- **Answer:** the comment is accepted and the sentences are reformulated

13. Tef Grain as Influenced by Nitrogen Fertilizer Rates and Varieties, Total Carbohydrate and energy content, line 328: Kindly adjust as follow: “(Table 2)”.

- **Answer**: the comment is accepted and adjusted

14. Tef Grain as Influenced by Nitrogen Fertilizer Rates and Varieties, Total Carbohydrate and energy content, lines 329–330: “On the other… increased”: The sentence is badly written in Standard English; accordingly, kindly reformulate it.

- **Answer:** the comment is accepted and the sentences are reformulated

15. Effects of Nitrogen Fertilizer Rates and Varieties on the Anti-nutritional Factor of Tef, Phytic acid content, line 337: Kindly adjust as follow: “(Table 3)”.

- **Answer**: the comment is accepted and adjusted

16. Effects of Nitrogen Fertilizer Rates and Varieties on the Anti-nutritional Factor of Tef, Phytic acid content, and line 339: Kindly adjust as follow: “was found”.

- **Answer**: the comment is accepted and replaced

17. Effects of Nitrogen Fertilizer Rates and Varieties on the Anti-nutritional Factor of Tef, Phytic acid content, lines 340–342: “The reason… contents”: The sentence is badly written in Standard English; accordingly, kindly reformulate it.

- **Answer:** the comment is accepted and the sentences are reformulated

18. Effects of Nitrogen Fertilizer Rates and Varieties on the Anti-nutritional Factor of Tef, Tannin content of tef grain, line 358: Kindly adjust as follow: “to reports”.

- **Answer:** the comment is accepted and the sentences are reformulated

19. Effects of Nitrogen Fertilizer Rates and Varieties on the Anti-nutritional Factor of Tef, Tannin content of tef grain, lines 359–360: “Tannin… table 3”: The sentence is badly written in standard English; accordingly, kindly reformulate it.

- **Answer**: the comment is accepted and the sentences are reformulated

20. Effects of Nitrogen Fertilizer Rates on Mineral Contents of Tef Grain, Iron (Fe), line 366: Kindly adjust as follow: “results are”.

- **Answer:** the comment is accepted and replaced accordingly

21. Effects of Nitrogen Fertilizer Rates on Mineral Contents of Tef Grain, Iron (Fe), line 367: Kindly adjust as follow: “were in the range of”.

- **Answer:** the comment is accepted and corrected

22. Effects of Nitrogen Fertilizer Rates on Mineral Contents of Tef Grain, Iron (Fe), line 370: “High… [39]”: The sentence is badly written in Standard English; accordingly, kindly reformulate it.

- **Answer:** the comment is accepted and the sentences are reformulated

23. Effects of Nitrogen Fertilizer Rates on Mineral Contents of Tef Grain, Iron (Fe), line 372: Kindly adjust as follow: “(Table 4)”.

- **Answer:** the comment is accepted and the sentences are reformulated

24. Effects of Nitrogen Fertilizer Rates on Mineral Contents of Tef Grain, Calcium (Ca), and line 385: Kindly adjust as follow: “(Table 4)” and “as compared to”.

- **Answer:** the comment is accepted and corrected accordingly

25. Effects of Nitrogen Fertilizer Rates on Mineral Contents of Tef Grain, Calcium (Ca), lines 386–389: “The maximum… respectively”: The sentence is badly written in Standard English; accordingly, kindly reformulate it.

- **Answer:** the comment is accepted and the sentences are reformulated

26. Effects of Nitrogen Fertilizer Rates on Mineral Contents of Tef Grain, Calcium (Ca), line 393: Kindly adjust as follow: “maximum Ca content”.

- **Answer:** the comment is accepted and adjusted

27. 386–389: “The maximum… respectively”: The sentence is badly written in standard English; accordingly, kindly reformulate it.

- **Answer:** the comment is accepted and the sentences are reformulated

28. Effects of Nitrogen Fertilizer Rates on Mineral Contents of Tef Grain, Calcium (Ca), line 394: Kindly adjust as follow: “observed with”.

- **Answer:** the comment is accepted and adjusted

29. Effects of Nitrogen Fertilizer Rates on Mineral Contents of Tef Grain, Calcium (Ca), lines 393–396: “On the other… varieties”: The sentence is badly written in Standard English; accordingly, kindly reformulate it.

- **Answer**: the comment is accepted and the sentences are reformulated

30. Effects of Nitrogen Fertilizer Rates on Mineral Contents of Tef Grain, Phosphorus content of tef grain, lines 401–402: “But… content”: The sentence is badly written in Standard English; accordingly, kindly reformulate it.

- **Answer**: the comment is accepted and the sentences are reformulated

31. Effects of Nitrogen Fertilizer Rates on Mineral Contents of Tef Grain, Phosphorus content of tef grain, line 404: Kindly adjust as follow: “in tef”.

- **Answer**: the comment is accepted and corrected

32. Effects of Nitrogen Fertilizer Rates on Mineral Contents of Tef Grain, Phosphorus content of tef grain, line 405: Kindly replace “have” by “had”.

- **Answer:** the comment is accepted and replaced

33. Effects of Nitrogen Fertilizer Rates and Tef Varieties on Sensory Quality of Injera, line 409: Kindly adjust as follow: “24 hours after”.

- **Answer**: the comment is accepted and adjusted accordingly

34. Sensory evaluation of injera, Color, lines 418–420: “Observing… rates”: The sentence is badly written in Standard English; accordingly, kindly reformulate it.

- **Answer**: the comment is accepted and the sentences are reformulated

35. Sensory evaluation of injera, Color, lines 421–425: “However… [42]”: Same recommendation as in the previous comment.

- **Answer**: the comment is accepted and the sentences are reformulated

36. Sensory evaluation of injera, Flavor, lines 427–429: “Only… product”: Same recommendation as in the previous two comments.

- **Answer**: the comment is accepted and the sentences are reformulated

37. Sensory evaluation of injera, Flavor, line 429: Kindly replace “have” by “had”

- **Answer:** the comment is accepted and replaced

38. Sensory evaluation of injera, Flavor, lines 431–432: “However… perceptions”: The sentence is badly written in Standard English; accordingly, kindly reformulate it.

- **Answer**: the comment is accepted and the sentences are reformulated

39. Sensory evaluation of injera, Flavor, line 435: Kindly remove “and rated as like-slightly”.

- **Answer:** the comment is accepted and adjusted

40. Sensory evaluation of injera, Texture, lines 437–438: “Injera… [42]”: The sentence is badly written in Standard English; accordingly, kindly reformulate it.

- **Answer:** the comment is accepted and the sentences are reformulated

41. Sensory evaluation of injera, Taste, lines 445–447: Same recommendation as in the previous comment.

- **Answer:** the comment is accepted and the sentences are reformulated

42. Sensory evaluation of injera, Taste, lines 448–455: “However… acceptance”: Same recommendation as in the previous two comments.

- **Answer**: the comment is accepted and the sentences are reformulated

43. Sensory evaluation of injera, Folding capacity (FC), line 457: Kindly adjust as follow: “(Table 5)”.

- **Answer:** the comment is accepted and the sentences are reformulated

44. Sensory evaluation of injera, Folding capacity (FC), line 459: Kindly replace “are” by “were”.

- **Answer:** the comment is accepted and the sentences are reformulated

45. Sensory evaluation of injera, Eye size (ES) and eye distribution (ED) of injera, lines 463–467: “Injera… injera”: These sentences are badly written in standard English; accordingly, kindly reformulate them.

- **Answer:** the comment is accepted and the sentences are reformulated

46. Sensory evaluation of injera, Eye size (ES) and eye distribution (ED) of injera, line 469: Kindly adjust as follow: “(Table 5)”.

- **Answer:** the comment is accepted and the sentences are reformulated

47. Sensory evaluation of injera, Eye size (ES) and eye distribution (ED) of injera, lines 478–480: “The ED… (7.33)”: The sentence is badly written in standard English; accordingly, kindly reformulate it.

- **Answer**: the comment is accepted and the sentences are reformulated

48. Sensory evaluation of injera, Eye size (ES) and eye distribution (ED) of injera, lines 483–485: “Therefore… injera”: Same recommendation as in the previous comment.

- **Answer:** the comment is accepted and the sentences are reformulated

49. Sensory evaluation of injera, Top and bottom surfaces (TBS) of injera, line 487: Kindly adjust as follow: “(p > 0.05)”.

- **Answer**: the comment is accepted and the sentences are reformulated

50. Sensory evaluation of injera, Top and bottom surfaces (TBS) of injera, lines 489–492: “Yetnberk… varieties”: These sentences are badly written in standard English; accordingly, kindly reformulate them.

- **Answer**: the comment is accepted and the sentences are reformulated

51. Sensory evaluation of injera, Overall acceptability of injera quality (OAIQ), line Sensory evaluation of injera, Overall acceptability of injera quality (OAIQ), lines 504–511: “Average… injera”: Same recommendation as in the previous comment.

- **Answer:** the comment is accepted and the sentences are reformulated

52. Sensory evaluation of injera, Total number of holes and filtered eyes of injera, lines 513–515: Same recommendation as in the previous two comments.

- **Answer**: the comment is accepted and the sentences are reformulated

53. Sensory evaluation of injera, Total number of holes and filtered eyes of injera, Number of holes of injera, lines 517–518: “The total… Table 6”: Same recommendation as in the previous comments.

- **Answer:** the comment is accepted and the sentences are reformulated

54. Sensory evaluation of injera, Total number of holes and filtered eyes of injera, Number of holes of injera, lines 520–525: “As comparing… affected”: Same recommendation as in the previous comments.

- **Answer**: the comment is accepted and the sentences are reformulated

55. Sensory evaluation of injera, Total number of holes and filtered eyes of injera, Number of holes of injera, line 527: Kindly adjust as follow: “implies that the application”.

- **Answer:** the comment is accepted and the sentences are reformulated

56. Sensory evaluation of injera, Total number of holes and filtered eyes of injera, Filtered eyes of injera, lines 532–533: “The filtered…injera”: These sentences are badly written in standard English; accordingly, kindly reformulate them.

- **Answer**: the comment is accepted and the sentences are reformulated

57. Sensory evaluation of injera, Total number of holes and filtered eyes of injera, Filtered eyes of injera, line 539: Kindly replace “to” by “and”.

- **Answer**: the comment is accepted and corrected

58. Sensory evaluation of injera, Total number of holes and filtered eyes of injera, Filtered eyes of injera, lines 547–549: “However… investigation”: The sentence is badly written in standard English; accordingly, kindly reformulate it.

- **Answer**: the comment is accepted and the sentences are reformulated

59. Sensory evaluation of injera, Colors (CIE L *a*b*) values of injera, Lightness (L) color of injera, line 553: Kindly adjust as follow: “determined the quality of injera”.

- **Answer:** the comment is accepted and the sentences are reformulated

60. Sensory evaluation of injera, Colors (CIE L *a*b*) values of injera, Lightness (L) color of injera, lines 553–563: “Injera… respectively”: These sentences are badly written in standard English; accordingly, kindly reformulate them.

- **Answer**: the comment is accepted and the sentences are reformulated

61. Sensory evaluation of injera, Colors (CIE L *a*b*) values of injera, Redness (a*) color of injera, line 565: Kindly adjust as follow: ‘significantly”.

- **Answer:** the comment is accepted and adjusted accordingly

62. Sensory evaluation of injera, Colors (CIE L *a*b*) values of injera, Redness (a*) color of injera, line 566: Kindly adjust as follow: “(Table 6)”.

- **Answer**: the comment is accepted and adjusted

63. Sensory evaluation of injera, Colors (CIE L *a*b*) values of injera, Redness (a*) color of injera, lines 566–567: “Nitrogen… varieties”: The sentence is badly written in standard English; accordingly, kindly reformulate it.

- **Answer**: the comment is accepted and the sentences are reformulated

64. Sensory evaluation of injera, Colors (CIE L *a*b*) values of injera, Redness (a*) color of injera, lines 570–576: “From grand… injera”: Same recommendation as in the previous comment.

- **Answer:** the comment is accepted and the sentences are reformulated

65. Sensory evaluation of injera, Colors (CIE L *a*b*) values of injera, Yellowness (b*) color of injera, line 594: Kindly adjust as follow: “(Table 6)”.

- **Answer:** the comment is accepted and the sentences are reformulated

66. Sensory evaluation of injera, Colors (CIE L *a*b*) values of injera, Yellowness (b*) color of injera, lines 594–601: “The ranges… rates”: These sentences are badly written in standard English; accordingly, kindly reformulate them.

- **Answer:** the comment is accepted and the sentences are reformulated

67. Sensory evaluation of injera, Colors (CIE L *a*b*) values of injera, Yellowness (b*) color of injera, line 602: Kindly replace “have” by “had”.

- **Answer:** the comment is accepted and adjusted

68. Sensory evaluation of injera, Colors (CIE L *a*b*) values of injera, Yellowness (b*) color of injera, lines 602–604: Kindly adjust as follow: “as compared to”.

- **Answer:** the comment is accepted and adjusted

5. **Conclusions**
1. Lines 608–609: “From the current… rates”: Kindly avoid the first voice form of the sentence and adopt the impersonal form instead.

- **Answer:** the comment is accepted and corrected accordingly

2. Line 610: Kindly remove “was” before “increased” and “decreased”.

- **Answer:** the comment is accepted and corrected accordingly

3. Lines 614–616: “Regardless… values”: The sentence is badly written in Standard English; accordingly, kindly reformulate it.

- **Answer:** the sentences are reformulated

4. Lines 616–617: “This may… society”: Same recommendation as in the previous comment.

- **Answer:** the comment is accepted and the sentences are reformulated

5. Line 618: Kindly adjust as follow: “as compared to”.

- **Answer:** the comment is accepted and replaced

6. Lines 618–624: “Analysis… dough”: These sentences are badly written in Standard English; accordingly, kindly reformulate them.

- **Answer:** the comment is accepted and the sentences are reformulated

Reviewer #2: Line 45, 73, 126, 330: review typographical or grammatical errors. Verify grammatical and typing errors throughout the document

- **Answer:** the comment is accepted and the sentences are reformulated accordingly
