# Supplementary material for: Influence of nitrogen fertilizer rate and variety on tef [Eragrostis tef (Zucc.) Trotter] nutritional composition and sensory quality of a staple bread (Injera)
Source: PLoS One. 2024 Jan 2;19(1):e0295491. doi: 10.1371/journal.pone.0295491 (PMC10760880; doi:10.1371/journal.pone.0295491)
Supplement: Attachment — Submitted filename: Response to reviewers.docx [file pone.0295491.s006.docx]

**Response to reviewers**

1. Please can you upload the latest version of your revised manuscript as the main article file, ensuring that does not contain any tracked changes or highlighting.

- Actually the manuscript was uploaded April 13, and it was reviewed. Here I uploaded the latest version of the manuscripts as the main article (without any track changes).

2. Please change the blue text in your manuscript with black text.

- I changed all the blue texts in the manuscript to black texts.
