# Supplementary material for: Influence of nitrogen fertilizer rate and variety on tef [Eragrostis tef (Zucc.) Trotter] nutritional composition and sensory quality of a staple bread (Injera)
Source: PLoS One. 2024 Jan 2;19(1):e0295491. doi: 10.1371/journal.pone.0295491 (PMC10760880; doi:10.1371/journal.pone.0295491)
Supplement: Attachment — Submitted filename: Response to reviewrs.docx [file pone.0295491.s007.docx]

**Response to the reviewers**

Line 96: Change “< 2-mm” to “< 2 mm”. Change “ml” to “mL”. Follow this style throughout the whole MS including Table values, captions, and footnotes.

- **Response**: We thank the reviewer for his comment. In the current version of the article, I have changed all the recommenced comments in the whole manuscript.

Line 122: Change “minutes” to “min”. Follow this style throughout the whole MS including Table values, captions, and footnotes.

- **Response**: We thank the reviewer for his comment. In the current version of the article, that I changed all the recommended comments with their respect of the whole manuscript including table values captions and footnotes.

Line 123: Change letter zero “48 hours for 28-30oC” to the symbol of degree “48 h for 28-30 °C”. Follow this style throughout the whole MS including Table values, captions, and footnotes.

- **Response**: In the current version of the article, we discuss and modified all recommended comments in the current revised version of the manuscript.

Line 124: Change “2 - 3 hrs” to “2 - 3 h”. Follow this style throughout the whole MS including Table values, captions, and footnotes.

- **Response**: In the current of this revised version of the manuscript; I tried to respond and answer all the recommended comments throughout the whole manuscript.

Line 224: Change “300ppm” to “300 ppm”. Follow this style throughout the whole MS including Table values, captions, and footnotes.

- **Response**: In the current version of the revised manuscript I made all corrections regarding the given comments, the unit and their magnitude.

Line 231: Change “μg / g” to “μg/g”. Follow this style throughout the whole MS including Table values, captions, and footnotes.

- **Response**: For this comment regarding the unit in the article, I made corrections in the revised manuscript in all body of the manuscript regarding to this specific comments.

Line 231: Change “Slpoe ×W × 3” to “Slope × W × 3”. Follow this style throughout the whole MS including Table values, captions, and footnotes.

- **Response**: We thank the reviewer for the comment. In the current version of the article, I made the corrections regarding the equation in the revised manuscript throughout the whole manuscript including table values, captions and footnotes.

Line 318: Change “P <0.001” to “P < 0.001”.

- **Response**: We thank the reviewer for the comment. In the current version of the article, I changed the Stastical values of the “P” value to the standard format throughout the whole discuss the differences between targeting the different diseases.

Line 320: Change “(table 3)” to “(Table 3)”. Many errors, address these errors.

- **Response**: We thank the reviewer for his comment. In the current version of the article, I changed the “Table format” throughout the whole body of the manuscript.

Line 323: Change “mg 100 g-1” to “mg 100 g^-1^”. Many errors, address these errors.

- **Response**: We thank the reviewer for his comment. In the current version of the article, I checked and made corrections regarding the superscript of the units throughout the whole body of the manuscript.

Line 359: Change “able 4” to “Table 4”.

- **Response**: We thank the reviewer for his comment. In the current version of the article, I made correction for the recommended comment regarding the standard Table format throughout the whole body of the manuscript.
